# Enablers and barriers to implementing a digital logistics system: A qualitative evaluation of OpenLMIS for Nigeria’s vaccine supply chain

**DOI:** 10.1371/journal.pgph.0005581

**Published:** 2026-09-15

**Authors:** Ifeoluwa Adebola Noiki, Lola Aladesanmi, Suruchi Gupta, Patricia Mechael, Erica Layer, Smisha Agarwal, Dustin Gibson

**Affiliations:** 1 BroadImpact, Abuja, Nigeria; 2 Department of International Health, Johns Hopkins Bloomberg School of Public Health, Baltimore, Maryland, United States of America; 3 Centre for Global Digital Health Innovation, Baltimore, Maryland, United States of America; 4 HealthEnabled, Capetown, South Africa; PLOS: Public Library of Science, UNITED STATES OF AMERICA

## Abstract

Logistics management information systems (LMIS) strengthen health commodity distribution and decision-making. Nigeria introduced OpenLMIS, an electronic LMIS, in August 2021 to track COVID-19 vaccines, and a routine immunisation (RI) module was deployed nationwide in 2023. This evaluation examined enablers and barriers to effective implementation and sustained use. A qualitative design combined desk reviews of LMIS documents, 42 key informants interviews, and 16 structured observational assessments with Cold Chain Officers. Data collection occurred in three states, the Federal Capital Territory, Lagos, and Niger, and nine Local Government Areas (LGA; three per state), which were purposively selected to capture diverse operational contexts. OpenLMIS has been rolled out across all 835 Nigerian cold stores at the zonal, state, and LGA levels. Adoption was driven by strong government stewardship (through policy prioritisation, allocation of personnel, and facilitation of site access), particularly the National Primary Health Care Development Agency (NPHCDA), and consistent financial and technical support from partners, including Gavi, the Vaccine Alliance, the Clinton Health Access Initiative (CHAI), and UNICEF. Persisting challenges include high staff turnover disrupting continuity; limited user training, often online-only, and insufficient where connectivity is poor; unreliable power and internet are hindering timely data entry and access; and inadequate, unsustained funding for data bundles, which shifts costs to users and undermines utilisation and data quality. OpenLMIS is advancing digitisation and visibility of Nigeria’s immunisation supply chain for informed decision-making. However, qualitative evidence from national stakeholders and the 3 sampled, states indicate that to maximise its benefits, stakeholders should address individual-level disincentives (e.g., turnover, training gaps), invest in reliable power and connectivity, and ensure sustainable financing for data and system operations alongside continued improvement in functionality.

## 1. Introduction

Logistics Management Information Systems (LMIS) are foundational for effective supply chain decisions within public health, managing critical data points such as stock levels, consumption rates, instances of losses, and detailed shipment information. Beyond mere record-keeping, their core functions extend to sophisticated forecasting, precise inventory and distribution planning, comprehensive reporting, efficient order fulfillment, and continuous performance monitoring [1]. Electronic LMIS (eLMIS) significantly enhances these capabilities, primarily by offering more timely data, improved data visibility across the supply chain, and facilitating seamless integration with Electronic Immunisation Registries (EIRs) [2,3]. Despite these advantages, many low- and middle-income countries (LMICs) continue to rely on paper-based systems, which often result in data quality issues and delays. While eLMIS facilitates real-time inventory tracking, streamlined workflows, and data-informed decision-making through digitalisation, their impact in many LMICs is frequently hindered by fragmented systems and unreliable infrastructure [4]. These systemic inefficiencies often manifest as frequent stockouts of life-saving vaccines and excessive wastage through the expiration of potent antigens, directly compromising immunisation coverage. Consequently, these logistics failures represent missed opportunities for disease prevention, leading to an increased burden of vaccine-preventable diseases and a failure to realize the full potential of public health interventions [5].

Prior to the implementation of OpenLMIS –an open-source, cloud-based eLMIS – Nigeria utilised various systems, including Microsoft Navision [5], which was discontinued due to financial and technical challenges, and an Open Data Kit (ODK)-based LMIS [6] that served as an interim solution. In 2021, the National Primary Health Care Development Agency (NPHCDA) and its partners strategically introduced the Open Logistics Management Information System (OpenLMIS) in Nigeria. This deliberate, government-led transition adoption was aligned with the Gavi Target Software Standards (TSS), underscoring a commitment to global best practices in health supply chain management. The primary objectives of this strategic move were to significantly enhance the efficiency of vaccine tracking, provide real-time visibility across the entire supply chain, and harmonize Nigeria’s existing LMIS infrastructure with established international standards [7]. The OpenLMIS implementation in Nigeria is a multi-tiered, cloud-based architecture designed to provide end-to-end visibility across the national vaccine supply chain. The system follows a strategic “push” model for stock management, where the National Strategic Cold Store (NSCS) acts as the central hub, distributing vaccines to 6 zonal, 37 state, 22 satellite, and 774 LGA-level stores based on population estimates and historical demand data. While the process begins with paper-based tools (VM1A/B forms) at the health facility level, data is consolidated at the LGA level and entered into the OpenLMIS web interface using tablets and laptops. Implementation responsibilities are shared between the NPHCDA, which provides government leadership and site access, and the Clinton Health Access Initiative (CHAI), which manages platform customization, deployment, and technical monitoring/support. To ensure data integrity, the system utilizes a layered verification process where digital records are cross-checked against physical stock counts during weekly reporting cycles. The OpenLMIS efficacy was first demonstrated through a successful pilot for COVID-19 vaccine management, conducted at both national and state levels in August 2021.

The positive outcomes of this initial implementation paved the way for the development and widespread deployment of a routine immunisation (RI) module in January 2023, extending its capabilities to cover a broader range of vaccine-preventable diseases across the country [8].

This paper presents an analysis of the adoption of OpenLMIS in Nigeria, examining the key enablers and barriers to its effective implementation and sustained use. Through a qualitative assessment involving desk reviews, key informant interviews, and structured observational assessments, we explore the user experience to identify key factors influencing the system’s success. The findings from this evaluation offer critical insights for strengthening digital supply chain management for vaccines in Nigeria and similar settings. While formal implementation science frameworks are valuable, this study was guided by a project-defined Theory of Change (ToC) to directly assess whether the underlying programmatic assumptions regarding system Inputs, capacity development, and digital literacy translated into the intended output of an effectively functioning system.

## 2. Methods

### 2.1. Study design

This was a qualitative study that comprised a desk review of relevant Logistics Management Information System (LMIS) documents in Nigeria (see S1 Annexe), project implementation updates/reports, key informant interviews (KIIs) with stakeholders, and observational assessments of OpenLMIS in practice.

The overarching objective of the study was to explore the utilisation, perceived benefits, and encountered Challenges of OpenLMIS within the context of routine immunisation programs and COVID-19 vaccination delivery in Nigeria. The study was designed to elicit insights into user experiences and gather information Regarding system functionality to inform future enhancements. The inclusion of a quantitative observational scoring tool within this qualitative study allowed for the objective triangulation of self-reported digital literacy with actual system navigation proficiency.

### 2.2. Ethics

Ethical approval for this study was granted by the National Health Research Ethics Committee (NHREC) in Nigeria, with approval number NHREC/01/01/2007–27/03/2024 and by Johns Hopkins Bloomberg School of Public Health (IRB00029552). Prior to participation in any interviews or observations, all individuals provided Informed consent, documented in written form. It was explicitly communicated and thoroughly emphasised that their involvement in the study was entirely voluntary, ensuring no coercion or undue influence. Participant recruitment spanned the period from 25^th^ June 2024–12^th^ July 2024.

### 2.3. Sampling and Sample Size

The study was conducted across three purposively selected states: the Federal Capital Territory (FCT), Niger, and Lagos. These locations reflect a level of geographical diversity, variations in existing infrastructure, and differing levels of OpenLMIS utilisation (low, medium and high), thereby ensuring a comprehensive understanding of the system’s performance in varied contexts. Utilisation classification was defined by the percentage of unique users, out of the 835 total cold stores, who logged in to perform stock management tasks within OpenLMIS on a weekly basis. Specifically, states were categorized as having Low (40–59%), Medium (60–79%), or High (80–100%) utilization. The selected sites represent distinct socio-economic and logistical environments: Lagos State is a densely populated coastal commercial hub in the Southwest, Niger State is a vast, predominantly rural landmass in the North-central region with significant Infrastructural reaches, and the Federal Capital Territory (FCT) serves as the administrative centre, hosting national-level stakeholders and high-density urban infrastructure. Within each of these states, the research included visits to the zonal cold store, the state cold store, and three distinct LGA-level cold stores.

A total of 42 in-depth key informant interviews (KII) were conducted with a range of stakeholders (program managers and cold chain officers) at the national, zonal, state, and LGA levels, capturing perspectives from across the entire supply chain hierarchy, except at the health facility level, where OpenLMIS is not currently deployed.

Complementing these KIIs, 16 systematic observational assessments of the Cold Chain Officers were carried out out at Zonal, State and LGA cold stores. These observations were critical for gaining practical insights into the platform’s actual usage, identifying workflow challenges, and directly assessing users’ proficiency in navigating and operating OpenLMIS in real-world settings.

Participants were identified and recruited through purposive sampling in collaboration with national and state-level immunisation program managers. Respondents were selected based on their direct involvement with the OpenLMIS platform and included Cold Chain Officers, Immunisation Program Managers, and LMIS focal persons across all tiers of the immunisation supply chain (National, Zonal, State and LGA). This approach ensured a range of perspectives from individuals in various roles, enhancing the relevance and applicability of the study’s findings. To minimize recruitment bias, participants were selected strictly based on their active role in operating or managing OpenLMIS, rather than performance metrics or referrals from immediate supervisors.

### 2.4. Theory of Change and Tools Development

Our study was guided by a Theory of Change (ToC, see S2 Annexe) that outlines how OpenLMIS is expected to strengthen vaccine logistics and, ultimately, immunisation outcomes. It operates on the logic that targeted development and capacity building will lead to improved data visibility, which ultimately strengthens the national health system. The components of the ToC include:

a. **Activities**: These are divided into System Development (customizing the platform and ensuring interoperability), System Inputs (managing daily data and monitoring), and Capacity Development (workforce training and technical support).
b. **Outputs**: Where successful activities result in an effectively functioning system with high data availability across all levels (LGA to National) and a workforce proficient in digital tools.
c. **Intermediate Outcomes**: These include increased political and donor buy-in, data-driven decision-making, and the sustained use of OpenLMIS for immunisation planning.
d. **Long-term Outcomes**: Where a mature digital health environment and enhanced service delivery is achieved, measured specifically by a reduction in vaccine stock-outs and decreased wastage.

This ToC informed the development of our study objectives and the design of our data collection tools, semi-structured interview guides and an observational tool.

These instruments were designed to test the ToC’s underlying assumptions by capturing stakeholder experiences and user digital literacy assessment. Interview questions examined system adoption and use, data flows and quality, decision-making processes, and perceived changes in stock visibility and vaccine delivery. The ToC also defined thematic domains for the interview guides and analytic framework, including enablers, barriers, user experience, functionality, and integration.

### 2.5. Data Collection Process

Experienced and trained research assistants (58% male; 42% female) conducted interviews using the semi-structured interview guides (See S3 Annexe and S4 Annexe). We conducted KIIs with national and sub-national program managers, cold-chain officers, and implementing partners. Data collection continued until thematic saturation was reached across three purposively selected states (Federal Capital Territory, Niger, and Lagos) and nine LGAs. Most interviews were conducted face-to-face, in English, and lasted 45–60 minutes.

Thematic saturation was continuously assessed during the data collection process and was determined when incoming data ceased to yield novel themes or operational categories. For Program Managers (n = 16) core themes regarding the system’s purpose and impact were established by the fourth interview and fully saturated by the seventh. Subsequent state-level interviews merely added regional context to already identified challenges. For Cold Chain Officers (n = 24): Saturation was reached between the fourth and fifth interviews. The remaining interviews across different states simply validated the initial findings on operational realities and technological barriers.

Interviews were audio-recorded for verification and quality checks. Observational assessments used a structured checklist (See S5 Annexe) to assess users’ ability to perform core OpenLMIS tasks, including: creating physical inventories, issuing orders, confirming shipments, making adjustments, transferring stock between programs, generate stock-balance/expiry/distribution reports, and manage cold chain equipment.

Interviewers also documented user feedback, challenges, and contextual factors observed during platform use. Prior to fieldwork, courtesy visits introduced the study to sites and addressed potential administrative barriers. Interviewers had no prior engagement with participants.

### 2.6. Data Analysis and Coding Process

Codes were developed deductively and inductively. Thematic categories focused on enablers, barriers, and user experience, system functionalities, and suggestions for improvement. All interviews and field notes were transcribed, then subjected to a stepwise thematic analysis process: 1) comprehensive reading for context; 2) open coding to label salient segments; 3) axial coding to cluster related codes and map relationships; and 4) selective coding to integrate core categories into themes aligned with research questions [9]. Coding was performed manually by two independent coders. To ensure intercoder reliability, 15% of the transcripts were independently double-coded. Any discrepancies in code application or theme development were resolved through consensus discussions among the research team. Observational assessments were included in coding for user-related factors, challenges and recommendations for the use of the OpenLMIS. Also, items on the checklist used for the observational assessment were assigned scores, and the overall performance score for each individual was calculated as a percentage, using the total number of applicable tasks as the denominator to account for sites where certain system features were not yet deployed. All observations were double-rated by two independent researchers, with any discrepancies resolved through consensus. Credibility of findings was strengthened through triangulation of KIIs, observations, and documents (identifying points of divergence and complementarity to further contextualise findings); enumerator debriefings (consensus meetings) to minimise bias and validate emerging interpretations; and stakeholder validation with select respondents to confirm the accuracy and relevance of emergent findings. Dependability was ensured by maintaining a detailed audit trail of coding decisions and analytical memos. Confirmability was supported by grounding the findings extensively in direct participant quotations.

### 2.7. Reflexivity Statement

The research team comprised individuals with backgrounds in public health and digital health implementations. Recognizing that our prior experiences with digital supply chain systems could introduce bias, we actively engaged in reflexivity throughout the study. Enumerators, who had no prior relationships with the participants, conducted the interviews to reduce social desirability bias. During data analysis, the team held regular debriefing sessions to acknowledge and set aside preconceived assumptions, ensuring that the resulting themes were strictly driven by the participants’ lived experiences and observed realities.

## 3. Results

### 3.1. Demographic Characteristics of the sample

We conducted a total of 42 KIIs and 16 observational surveys (see Table 1). KIIs were 63% (n = 26) male and 37% (n = 16) female (See Table 1). By role, 43% (n = 18) were cold chain officers and 57% (n = 24) were program managers overseeing immunisation supply chain functions. The distribution of KIIs across administrative levels was as follows: 38% (n = 16) were at the LGA level, 31% (n = 13) at the State level, 10% (n = 4) at the Zonal level, and 21% (n = 9) at the National level. The distribution of KIIs by administrative level, stakeholder role, and organization for the three states are presented in Table 2.

**Table 1:** Demographic characteristics of key informant interview (n = 42) and observational assessment participants (n = 16).

	Key Informant Interviews	Observational Assessment
**Demographic**	**n (%)**	**n (%)**
**Gender**		
Male	26 (63)	11 (69)
Female	16 (37)	5 (31)
**Stakeholder Role**		
Program Manager	24 (57)	2 (13)
Cold Chain Officer	18 (43)	14 (88)
**Administrative level**		
National	9 (21)	2 (13)
Zonal	4 (10)	2 (13)
State	13 (31)	4 (25)
Local Government Area (LGA)	16 (38)	8 (50)

**Table 2:** Distribution of key informant interview participants by administrative level, stakeholder role, and organization.

National/State	Administrative level	Stakeholder role	Organization	# of interviews
National (n = 9)	National	Program manager	NPHCDA	7
			UNICEF	1
			CHAI	1
FCT (n = 7)	State	Program manager	SPHCDA	1
			UNICEF	1
		Cold chain officer	SPHCDA	2
	LGA	Cold chain officer	SPHCDA	3
Lagos (n = 12)	Zonal	Cold chain officer	SPHCDA	2
	State	Program manager	SPHCDA	2
			UNICEF	1
		Cold chain officer	SPHCDA	1
	LGA	Program manager	SPHCDA	4
		Cold chain officer	SPHCDA	2
Niger (n = 14)	Zonal	Cold chain officer	SPHCDA	2
	State	Program manager	SPHCDA	2
			UNICEF	1
		Cold chain officer	SPHCDA	2
	LGA	Program manager	SPHCDA	3
		Cold chain officer	SPHCDA	4

Abbreviations: **CHAI**, Clinton Health Access Initiative; **FCT**, Federal Capital Territory; **LGA**, local government area; **NPHCDA**, National Primary Health Care Development Agency; **SPHCDA**, State Primary Health Care Development Agency;

### 3.2. Perceived benefits of OpenLMIS

OpenLMIS has been rolled out across all 835 Nigerian cold stores, encompassing Zonal, State, and LGA levels. The integration of OpenLMIS at higher levels of the vaccine supply chain has resulted in enhanced efficiency, a shift in logistics personnel roles, and more proactive supply Chain Management. This evolution underscores the escalating importance of digital tools and data-driven strategies in logistics, promoting enhanced accountability, transparency, and necessitating continuous skill development for a workforce more adept at leveraging technology for improved public health outcomes.

*“The use of OpenLMIS has led to streamlined processes and reduced paperwork, allowing staff to focus more on vaccination utilization. It has also enhanced accountability, better tracking of stock and transparency. Some staff roles have also shifted to include more data management and analysis tasks”- State Program manager, SPHCDA*

The efficiency brought about by OpenLMIS allows for a more proactive approach to the supply chain management. By providing real-time data and insights, the system enables stakeholders to identify and mitigate potential issues before they escalate into larger crises. This foresight ensures a more reliable and responsive system for critical vaccine distribution, minimising stockouts, optimising cold chain integrity, and ultimately improving public health outcomes by ensuring vaccines reach their intended recipients effectively and consistently.

*“OpenLMIS enhances the management of vaccine supply chains by providing real-time data on vaccine stock levels, distribution, and usage. This improved data visibility allows us workers and our senior management to make informed decisions about vaccine utilisation across all levels within the state.” – State Program manager, SPHCDA*

### 3.3. Perceived Enablers and Barriers to OpenLMIS adoption and use

The study identified several perceived factors that influenced the adoption and use of OpenLMIS in Nigeria.

These factors, grouped into organizational/environmental, user-related, and technological themes, encompass the different enablers and barriers that shape the environment for the implementation and sustainability of the OpenLMIS across Nigeria (See Table 3).

**Table 3:** Summary of perceived enablers and barriers to OpenLMIS adoption by theme.

Theme	Factor	Examples
Organizational/ Environmental	Government and partner support **I**	Strong leadership from NPHCDA and consistent support from Gavi, CHAI, and UNICEF partners
	Financial constraints **(B)**	Lack of sustainable funding for internet data forces users to pay out-out-pocket
	Inadequate training **(B)**	Often inconsistent, insufficient, or delivered virtually with poor connectivity
	Insufficient local user-support **(B)**	Error corrections requires national remediation leading to delays and disruptions
	Infrastructure and equipment gaps **(B)**	Unreliable internet connectivity and power supply with no offline mode availability
User-related	Financial disincentives **(B)**	Staff often fund internet data from their own salaries thus reducing motivation to use
	Human resource management **(B)**	High staff turnover, often due to political appointments, disrupts knowledge continuity
	Increased workload **(B)**	At the LGA, requirements for dual data entry (paper and OpenLMIS) seen as burdensome as inefficient
	Digital literacy and platform proficiency**(E)**	High proficiency in using platform by cold chain officers
Technological	Highly valued platform features **(E)**	Users praised features like automated reminders for vaccine expiration and data validation checks
	Platform features needing improvement **(B)**	Users reported slow system speed, a cumbersome user interface, and system errors like generation of duplicate IDs
	Requested platform features **(B)**	Key features requested include offline client, barcode scanning (in bulk) and interoperability with other systems

Abbreviations: **(B)**, Barrier; **(E)**, Enabler; **CHAI**, Clinton Health Access Initiative; **LGA**, local government area; **NPHCDA**, National Primary Health Care Development Agency;

#### 3.3.1 Organizational/ Environmental factors.

***Government commitment and partner support:*** The initial rollout of OpenLMIS was driven by strong political will from the NPHCDA and its National Logistics Working Group (NLWG), which demonstrated commitment through strategic policy directives, the allocation of human resources at cold stores, and facilitating site access. However, this commitment did not extend to sustained financial ownership for operational costs, such as internet data provisioning, which remained a significant barrier.

Operational costs were covered by key implementing partners such as CHAI, Gavi, and UNICEF. This support was critical for both the initial rollout and the sustained utilization of the OpenLMIS system across Nigeria.

*“Technical support from the partners and NPHCDA has been very crucial”- State Program manager, SPHCDA*

Gavi, CHAI, and UNICEF provided multifaceted support. This encompassed the initial onboarding training and periodic refresher training sessions, which were facilitated by CHAI and the NPHCDA. Furthermore, UNICEF offered ongoing technical support, primarily delivered through convenient and accessible channels such as WhatsApp and Zoom. This support helped users resolve operational issues and maximize the full functionality of the system, facilitating usage. A notable observation was that higher levels of engagement with OpenLMIS were consistently observed in states and LGAs that demonstrated strong leadership and maintained consistent follow-up protocols.

### Financial constraints

Financial constraints were identified as a critical challenge, primarily due to the lack of sustainable funding for internet data, which poses ongoing difficulties for OpenLMIS implementation and usage. While external support from CHAI and UNICEF provided temporary relief, the absence of a long-term financial plan leaves the system vulnerable to disruptions.

*“When the roll-out came; there was no support for data provision and OpenLMIS is data intensive; CHAI however provided support for this at the LGA level and also provided training and re-training. CHAI also provides virtual support and training. However, the major issues are now as regards the provision of internet services.” - State Program manager, SPHCDA*

This absence of dedicated funding also affects the perceived value of OpenLMIS, as resource allocation from government offices to support its implementation has been inadequate. There is a perceived need for increased government ownership of the OpenLMIS implementation process to ensure its sustainability and maximise its benefits. This includes establishing consistent budget lines for internet connectivity, maintenance, and ongoing training to prevent service interruptions and ensure’ the system’s longevity.

### Inadequate training

The effectiveness of OpenLMIS capacity-building activities provided by CHAI were hampered by inconsistencies in both the frequency and quality of sessions. Training programs were often irregular and, at times, demonstrably insufficient, underscoring a pressing need for more consistent and comprehensive educational initiatives.

*“Capacity building was a real issue for adoption; it took a lot of training and retraining to understand how the system works.”- State Program mana*ger, SPHCDA

This was particularly critical for new users or individuals who had missed previous sessions, as their lack of initial exposure significantly impeded their ability to fully leverage the system. This challenge was acutely observed in states with low OpenLMIS utilisation, where participants frequently cited incomplete training or recent employment as primary reasons for their limited proficiency across various system functionalities.

The initial rollout for COVID-19 vaccination, for instance, relied heavily on virtual training modules. However, these were largely met with resistance and disinterest from some stakeholders, primarily due to existing digital literacy gaps and significant participation issues stemming from unreliable internet access, especially prevalent at the state and LGA levels.

### Inadequate local user-support

Another significant impediment to the system’s effectiveness is the lack of localized technical support, particularly for correcting errors that arise from user input or system malfunctions. Currently, many system errors, such as duplicate transaction IDs or discrepancies between system data and downloaded reports, require intervention from national-level personnel. This centralised troubleshooting method causes substantial delays, interrupts user workflows, and reduces overall efficiency.

*“If I make a mistake, I can’t correct it and that mistake is what will be seen on the OpenLMIS. The OpenLMIS desk officer in Abuja should try from time to time to be able to make corrections since we can’t delete our mistakes by 0urselves.” – Zonal Cold Chain Officer, SPHCDA.*

The absence of readily available local technical assistance means that issues that could be resolved at a lower administrative level are instead escalated, negatively affecting system performance and user morale. This Reliance on a central authority for even minor technical issues creates a bottleneck, leading to frustration among users and a slower resolution time for critical problems.

### Infrastructural and equipment gaps

Infrastructural limitations were identified as a critical impediment to the effective implementation and sustained use of OpenLMIS. A primary concern is the unreliable internet connectivity prevalent across many regions of Nigeria. This issue is exacerbated by the current non-utilization of the OpenLMIS offline client (yet to be configured by the system administrators at the time of this study), which renders users entirely dependent on consistent internet access—a resource that is frequently unavailable or unstable, particularly in remote areas and at lower levels of the supply chain. The resulting persistent network downtime disrupts operational workflows, leading to delays in task completion and subsequently discouraging consistent system adoption. Beyond connectivity, other critical infrastructural deficiencies compound these challenges, notably an inconsistent power supply. In regions afflicted by unreliable electricity, users encounter substantial difficulties in maintaining the charge of their computing devices, further curtailing their capacity to engage with the system consistently.

Furthermore, frequent concerns were raised regarding the availability, maintenance, and security of computing equipment. Reports of damaged or stolen devices directly underscore how these issues impact the accessibility and sustained use of OpenLMIS in specific locations. These widespread infrastructural challenges collectively contribute to a less than optimal environment for the full realization of OpenLMIS’s potential.

#### 3.3.2 User-related factors.

***Financial Disincentives:*** A primary barrier to user motivation stems from prohibitive data costs. Personnel, especially at the LGA level, often fund internet services out-of-pocket due to a lack of dedicated budgetary allocations.

*“Out-of-pocket funding of data has been a huge challenge; with delays in paying salaries, which is not unique to just the* *supply chain but to service delivery in general.” - National Program manager, NPHCDA*

This issue is exacerbated by broader financial constraints, including delayed salaries and limited operational budgets, which collectively reduce staff commitment and motivation. The absence of financial incentives, such as performance-based bonuses or allowances for data usage, further demotivates staff from consistently engaging with the OpenLMIS system.

### Human Resource Management

Frequent turnover of personnel, largely driven by political leadership/governance and influence in human resource decisions, significantly disrupts the continuity and institutionalization of OpenLMIS knowledge.

*“Change in governance often affects the appointment of the cold chain officers… creating a huge gap in knowledge and competency.”- National Program manager, NPHCDA*

Newly appointed staff members often lack the necessary skills and receive insufficient or no training, leaving them unprepared to effectively use the system. This lack of adequate human resources, both in terms of quantity and quality, poses a significant barrier to the sustainable adoption and effective utilization of OpenLMIS. The constant need to onboard and train new staff diverts resources and time, hindering progress and potentially leading to errors or underutilization o’ the system’s capabilities. Furthermore, the absence of a stable and skilled workforce undermines efforts to build internal capacity and create a resilient system that can adapt to evolving needs and challenges.

### Increased Workload

At the LGA level, the requirement to maintain parallel paper-based and electronic records creates a substantial increase in workload. This dual data entry is perceived as burdensome, as the cold chain officers need to collate paper-based records from several health facility stores and enter them manually into the LGA store records and then into OpenLMIS. In Nigeria, the requirement for this parallel paper-based and electronic record-keeping at the LGA level is driven by a combination of infrastructure limitations (limited power supply and internet connectivity), system fragmentation (presence of multiple non-interoperable systems), and data verification protocols when receiving and distributing stock to lower levels who do not use the OpenLMIS.

*“It has added to our problem because we don’t have supporting hands. We enter the ledger and the OpenLMIS at the same time, so OpenLMIS adds to our workload”- LGA Cold chain officer, SPHCDA.*

This duplication leads to inefficiencies and, in some cases, data entry errors, particularly when handling large volumes of data or multiple tasks. The time-consuming nature of this process diverts staff from other critical duties, potentially impacting overall supply chain efficiency and accuracy. In contrast, at higher levels of the Supply chain (State, Zonal, and National), OpenLMIS has been perceived to positively impact workflows by significantly reducing reliance on manual paperwork. This digitalization has streamlined logistics processes, allowing staff to reallocate their focus to more critical tasks such as vaccine data analysis and utilization for informed decision-making.

### Digital literacy and platform proficiency

Findings from observational assessments indicate a generally high level of OpenLMIS knowledge, proficiency, and digital tool literacy among cold chain officers. Observational performance was calculated as a composite percentage. For each participant, successfully completed tasks were scored as 1 and incomplete/failed tasks as 0.

The overall score of 85.4% represents the average proportion of applicable tasks successfully completed across all 16 observed users. (See Table 4).

**Table 4:** Observational assessment of cold chain officer’s ability to complete OpenLMIS tasks.

OpenLMIS task	n (%)
Create physical inventory	15 (94)
Issue an order	15 (94)
Confirm shipment	13 (81)
Make stock adjustments	16 (100)
Transfer stock from one program to another	14 (88)
Generate stock balance report	16 (100)
Generate report showing stock expiry status	11 (69)
Generate stock distribution report	12 (75)
Manage cold chain equipment	11 (69)
**Total**	**85.4%**

#### 3.3.3 Challenges with Specific OpenLMIS Tasks/Functions.

Despite the overall high proficiency, certain functionalities within OpenLMIS consistently presented difficulties for users, suggesting areas ripe for targeted improvement. In creating physical inventory, users frequently described this process as cumbersome, particularly when handling bulk submissions of inventory data. They suggested improvements such as allowing individual product updates, which would provide more flexibility, and the ability to hide out-of-stock or expired batches directly within the OpenLMIS interface to reduce visual clutter and potential for error.

*“I should be able to update products individually” - State Cold Chain Officer, SPHCDA.*

*“I’d prefer to be able to hide every batch that is zero, to make the interface less cumbersome” – National Program manager, NPHCDA*

For issuing orders, users reported challenges related to accurate product listing and, notably, the lack of system functionality to withdraw or correct previously issued products. This rigidity led to recommendations for system enhancements, including features like auto-summation of vaccine quantities to prevent manual calculation errors and better management of product expirations to ensure only viable stock is issued.

*“We need to upgrade the system in such a way that, when we issue out to a facility and discover they don’t use It we should be able to withdraw it and issue it out to another facility that need it–urgently.” – LGA Cold Chain Officer, SPHCDA.*

For managing Cold Chain Equipment (CCE), users reported that this functionality was a significant challenge, largely attributed to insufficient training provided to users and the inherent complexity of the system’s interface for this specific task. Users expressed a strong need for more comprehensive training modules dedicated to CCE management and fundamental system improvements to simplify the process.

For confirming shipments, some users advocated for improved external communication protocols (e.g., automated notifications when shipments are dispatched) and enhanced record-keeping capabilities within OpenLMIS to facilitate easier reconciliation and verification of incoming deliveries.

*“If I have 200 antigens and want to make 210, when I click on the antigen there should be a notification that will show–my stock.” - LGA Cold Chain Officer, SPHCDA.*

*“It will be better if we can get notifications for low stock levels or upcoming expirations via SMS or email to serve as the reminder” - LGA Program manager, SPHCDA*

Conversely, “generating stock reports” (encompassing balance, expiry, and distribution “reports), “rectifying stock balance,” and “transferring stock items” were generally found to be relatively easy for users to perform.

Exceptions occurred primarily in cases where prior training was clearly lacking, underscoring the importance of adequate instruction across all functionalities.

#### 3.3.4 Technological factors.

***Highly-valued platform features:*** The most appreciated functions of OpenLMIS included reminders and data validation capabilities. Reminder and alert functions were highly valued by users as it significantly aided in monitoring vaccine expiration dates, preventing stockouts by prompting reorders, and ensuring accurate physical inventory counts through timely notifications for reconciliation. However, a recurring point of feedback was the inconsistency of these alerts, with some users reporting delayed or missed notifications. In regards to data validation’ the system’s ability to detect and highlight data entry errors, such as incorrect vaccine counts, incorrect unit of measure, or discrepancies between expected and actual inventory levels, was also a frequently praised feature. This functionality helped maintain data integrity and reduce manual reconciliation efforts.

### Platform features needing improvement

According to users, OpenLMIS needs significant improvement across several key areas to enhance its effectiveness and user satisfaction. A primary concern among users is the overall system performance, specifically the reported slow system speed hinders efficient operations. This is likely linked to poor Internet connectivity and requests for offline capabilities were requested.

*“If we can use it offline, so we can use it even where there is no network connection…because without data, you cannot open it, and it’s really frustrating at times when loading”- Cold chain officer*

Furthermore, browser compatibility issues frequently arise, leading to an inconsistent user experience.

The user interface (UI) is often described as cumbersome and overly complex, making navigation and data entry challenging. A recurring pain point is the difficulty users encounter when attempting to update stock records, a critical function for inventory management.

Users encounter various system errors that compromise data integrity, such as the generation of duplicate transaction IDs, which can lead to confusion and inaccuracies. Discrepancies between the data displayed within the system and information presented in the downloaded reports have also been noted, undermining trust in the system’s accuracy. Challenges persist in extracting comprehensive summary reports, limiting the ability to gain quick insights into logistics operations. Accessing historical transaction receipts for auditing and reconciliation purposes has proven difficult, as has the efficient archiving of data for long-term trend analysis and strategic planning. Lastly, the integration and recording of Cold Chain Equipment (CCE) data present significant difficulties for users. The under-utilization of this crucial feature is often attributed to inadequate training provided to users on its functionalities and a general lack of user-friendliness within the CCE module, deterring its effective adoption and use.

### Requested platform features

Several OpenLMIS functions (yet to be configured into Nigeria’s OpenLMIS) were identified as crucial for improving its effectiveness, including: barcode scanning (preferably at the carton level for high volumes) to enhance stock-taking efficiency and expedite data entry; temperature monitoring to ensure vaccine integrity; offline client to decrease dependence on internet connectivity (as only the web-based version is currently in use); and interoperability to connect logistics data with service delivery data, such as vaccine utilization reports from the National Health Management Information System (NHMIS-DHIS2) and the Electronic Management of the Immunisation Data (EMID) platform, reducing reliance on manual triangulation via platforms like Thrive360.

## 4. Discussion

This qualitative study of Nigeria’s national OpenLMIS rollout provides critical insights into the complex interplay of factors shaping the adoption and use of digital health technologies in an LMIC setting.

The deployment of OpenLMIS across all 835 cold stores demonstrates a significant operational achievement made toward strengthening Nigeria’s vaccine supply chain. However, our findings reveal that this implementation is moderated by persistent challenges related to workforce capacity, financial ownership, and foundational infrastructure, which threaten the system’s long-term sustainability and reflect broader themes in the global dialysis health literature [10].

A primary learning from this evaluation is that strong government ownership is a critical enabler for success – which is in line with the 1^st^ key assumption on the ToC; “ Government buy-in exists holistically across relevant agencies and Ministries”.

The leadership of the NPHCDA, and supported by partners like Gavi and CHAI, was perceived to be one of the key drivers of the national rollout. This lesson is similar to findings from a multi-country evaluation, which found that full government ownership was essential for the long-term success of digital health tools [11].

In Rwanda, the government’s decision to build its immunisation registry on the national DHIS2 platform developed local expertise and ensured sustainability. In contrast, there was an over-reliance on external partners for a customised system in Tanzania, which led to its discontinuation in many facilities once external support was phased out [11]. The need for local empowerment is further underscored by our own findings, which revealed that the reliance on national-level personnel for troubleshooting and error resolution created significant delays and user frustration, which may impact system efficiency. However, it is important to distinguish between strong programmatic ownership demonstrated by the NPHCDA’s leadership and the still-maturing state of its financial ownership. Our findings show that while the government has strong stewardship over policy and implementation, the long-term operational costs, such as internet data for users,are not yet secured through dedicated domestic budget lines; thereby creating a sustainability paradox where the system is a national priority, yet its daily functioning remains vulnerable to out-of-pocket payments by frontline workers. This threat of donor dependency is further exemplified by experience in Ethiopia, where a successful mobile stock management tool was abandoned after project funding ended and with no exit or transition strategy in place [12]. This situation reflects a common stage in the digital health maturity model, where political will often outpaces the slower process of integrating a system’s recurrent costs into the national budget.

Beyond sustainability, the effectiveness of the system is consistently challenged by foundational human resource and infrastructure gaps. Issues of high staff turnover, inconsistent user training, and the burdensome requirement of dual data entry are not only operational hurdles, but systemic weaknesses.

These challenges reflect a well-documented pattern in LMICs, where fragmented health information systems and weak human resource planning strain the health workforce and can lead to burnout, ultimately undermining the utility of digital tools [13]. Similarly, the obstacles of unreliable internet and power remain pervasive barriers to digital health adoption across the continent, limiting the potential of otherwise well- designed systems [14]. These findings validate the ToC’s output; specifically, the achievement of improved digital tool literacy, as evidenced by the 85.4% observational task completion rate, enabled by technical assistance and training. However, several fundamental ToC assumptions were directly challenged by the operational reality. For example, assumption 3 (stable and adequate health workforce) was undermined by high staff turnover driven by political appointments. Also, assumption 5 (uninterrupted power and connectivity) was consistently unmet, rendering the system vulnerable to infrastructure gaps.

This study has several limitations. First, interviews and observational assessments were conducted in three purposively selected states. While selected to represent diverse operational contexts, these sites may not be representative of the experiences across all 36 states in Nigeria, limiting the generalizability of our findings.

Second, our data collection relied on key informant interviews which may be subject to social desirability bias. We sought to mitigate this through triangulation with observational data and a review of system documents. Furthermore, our evaluation was qualitative in nature and did not include a secondary analysis of quantitative data from the OpenLMIS system itself, which could have provided objective metrics on the system utilization and reporting.

Lastly, this study focused exclusively on the use of OpenLMIS for the routine immunisation program; the findings may not be applicable to its use for other health commodities or in different program areas.

In conclusion, the national scale-up of OpenLMIS represents a significant achievement in Nigeria’s progress toward a digitally enabled vaccine supply chain. The adoption and use of the system, driven by strong government ownership and partner collaboration, provides a model for other large-scale digital health implementations. However, as evidenced by our findings at the national level and within the three sampled states, translating this system deployment into long-term, sustainable success requires targeted interventions. Stakeholders must address persistent challenges, like high staff turnover, inconsistent user proficiency, and infrastructure gaps. Realizing the full potential of OpenLMIS will require a continued investment in both the technology and the people, and systems that support it.

## Supporting information

S1 Annexe List of Documents Reviewed. (DOCX)

S2 Annexe Theory of Change: OpenLMIS implementation and use in Nigeria. (DOCX)

S3 Annexe Key Informant Interview Guide: Implementers and Program Managers. (DOCX)

S4 Annexe Key Informant Interview Guide: Cold Chain Officers. (DOCX)

S5 Annexe OpenLMIS Observation Checklist. (DOCX)

S1 Checklist Inclusivity in global research. (DOCX)

S1 Data Qualitative interviews. (PDF)

S2 Data Observational assessment. (PDF)
