# Supplementary material for: Enablers and barriers to implementing a digital logistics system: A qualitative evaluation of OpenLMIS for Nigeria’s vaccine supply chain
Source: PLOS Glob Public Health. 2026 Sep 15;6(9):e0005581. doi: 10.1371/journal.pgph.0005581 (PMC13577329; doi:10.1371/journal.pgph.0005581)
Supplement: Supplementary file 1 [file pgph.0005581.s007.pdf]

Participant No. 001
Location: FCT

Respondent's Gender: **Female** ☐

A. OpenLMIS and its role within the EPI Programme and beyond

1. What was the rationale for the rollout process of OpenLMIS for COVID-19 vaccination and subsequently Routine Immunization (RI) implementation?

It's efficient to have a system to help monitor vaccine utilization. It saves one the cost for what is required. Before the introduction of the OpenLMIS, there were previous tools being used, from solely paper-based to using SMS technology. The introduction of the OpenLMIS helps us to track transactions, providing dashboaord visualizations to help with planning, and shipments to the lower levels. The OpenLMIS is being used similarly for COVID and RI; For COVID its just a few antigens; for RI is more antigens but the system is being navigated the same way.

2. Describe the role OpenLMIS currently plays within the EPI program.

When registering vaccines; it's either for RI or SIAs; the system helps with registering the quantities for the different programs. The OpenLMIS is actively being used for RI and Campaigns. The OpenLMIS helps to show the different quantities accordingly for either RI or Campaign; this helps to determine and plan, comparing the available stock to what is required from the national level.

Every Wednesday, the VSM dashboard is looked into, where updates on events from the previous week are provided. Looking at the stock levels; at zonal levels first. The legends help to see areas that need vaccines the most and this helps for prioritization.

- The OpenLMIS also shows the stock on hand and how long it's estimated to last (in number of weeks), and it informs the allocation meetings and stock on hand. It helps to also see antigens at critical levels, which then informs if to call for shipments or track those already on PO or get more funds to place more vaccines on PO

3. How has OpenLMIS impacted your decision-making and program management?

-

4. To what extent is the OpenLMIS interoperable with other information systems (DHIS2)?

-

5. Is the OpenLMIS already being used in other health areas (i.e. for other health commodities)

- Not yet, however, additional modules are also being looked into for example; RMNCH, and Nutrition are currently being considered.

B. Barriers and facilitators to adoption, data quality, and use of OpenLMIS

6. What are the main barriers to the adoption, use, and data quality within OpenLMIS?

- At the beginnning we had almost >90% of utilization at LGA level - It began to drop after changes in personnel done by the state government (political influence);

- Internet availability is also another factor that has reduced utilization of the OpenLMIS.
- Areas where there have been declines in usage of the OpenLMIS; we have tried to retrain new CCOs and solicit for data funds to be provided on time; also considering the fact that payment of salaries at the LGA level is always difficult.
- There have been few instances of stolen/missing or non-functional tablets as well as behavioral issues.
- Commitment is a major factor that has contributed to variations in utilization. We follow up through the state CCOs; also part of the mandatory agenda item during the LCCO meetings is on the OpenLMIS and its usage.
- The level of commitment has been a major factor for states with higher performances; we have also made use of platforms to reward and award high-performing states and their personnel.

7. What factors have facilitated the successful implementation and use of OpenLMIS?

C. Perceptions on the link between OpenLMIS data use and immunization coverage

8. Have you noted any links between OpenLMIS data use and immunization coverage, completion, and timeliness?
   - If we are able to understand the areas that need vaccines and we push to those places; it will improve coverage because they have more vaccines. In the situation where we don't know the need, it will be hard to meet the needs.
   - We also try to triangulate OpenLMIS with the DHIS2 for utilization; the operation room also has interactions (with the VSLs, State CCOs and others) to discuss vaccine levels and issues around utilization and logistics.
   -
   -

9. Has the use of the OpenLMIS led to any unexpected effects on vaccine delivery or staff roles? Describe these changes.

D. Learnings and recommendations from OpenLMIS utilization for COVID-19 vaccination

10. What unique lessons were learned (positive and negative) from using OpenLMIS for COVID-19 vaccination, and what recommendations do you have based on these lessons that can be applied to future large-scale campaigns and outbreak response in Nigeria or other countries?

    - Data quality is important, wrong information can lead to misallocations. It is something we are always keen on or following up on. Even with the OpenLMIS at the beginning, we had to still use google sheets side-by-side, but it's been used less and the OpenLMIS is a bit more comfortable to use.
    - Poor internet contributes to slow loading of the OpenLMIS. It's hard to remember if there were any instances when the platform was down.
    - There are additional features needed (which have also been requested to Gavi); such as data archiving, CCE Bin tracking, vendor management, weekly stock snapshot, vaccine vial retrieval management, interoperability with TRVST for vaccine verification and

traceability, and mobile application.

- The app is good and is more user-friendly than previous tools that have been used.

Participant No. 002
Location: FCT
Respondent's Gender: **Male**

A. OpenLMIS and its role within the EPI Programme and beyond

1. What was the rationale for the rollout process of OpenLMIS for COVID-19 vaccination and subsequently Routine Immunization (RI) implementation?

- For some years, we had used NAVISION from national to state levels for about 2 years; but was discontinued because of the several steps to complete transactions and it non-user friendliness. After which we engaged Feild Intelligence to customize Navision but still didn't last because of the cost of maintenance; this ended in April 2020. We started scoping for an eLMIS using the guidance provided by Gavi and GF to ensure global standards are met; after which Gavi engaged CHAI to manage the scoping of the new eLMIS; CHAI was selected being part of the NLWG and also because they are home-based.
- We started rolling out the COVID module during the pandemic; and there were challenges because it was a new tool and also because the supply chain activities during COVID was quite hectic. By and large it was good start and helped us to have better utilisation for RI and SIA.
- In terms of operations the system is being used the same way.

2. Describe the role OpenLMIS currently plays within the EPI program.

-

3. How has OpenLMIS impacted your decision-making and program management?

- I manage the data for the country; and it is my duty to make sure that all locations are followed up with. It's a day-to-day responsibility and I have to report particularly on Wednesday and share all stock issues across the supply chain from National to LGAs
- On a weekly basis, issues are flagged to the NLWG and where there are vaccines close to expiry; we advise for redistribution; this is similarly done for stores where there's overstock.
- For example there's overstock of 2ml syringes and we have had to go out through the NLWG to see other programs that make use of 2ml syringes and distribute accordingly.

4. To what extent is the OpenLMIS interoperable with other information systems (DHIS2)?

- Gavi has provided the grant for the timeline to be interoperable with DHIS2 by September and also temperature monitoring devices at the facility level (who will be using a different app entirely hence the need for interoperability).

5. Is the OpenLMIS already being used in other health areas (i.e. for other health commodities)

- As a country the OpenLMIS is only used in the immunization program; there are plans to however expand the OpenLMIS for other commodities. The head of drug and food services has attested to the fact that the tool being used is expensive and not sustainable. The country is looking for an affordable system. There have been discussions and meetings on going to explore the possibility of expanding the OpenLMIS.

B. Barriers and facilitators to adoption, data quality, and use of OpenLMIS

6. What are the main barriers to the adoption, use, and data quality within OpenLMIS?
   - At the high level; there are many conflicting activties especially in the Logistics department where the human resources are not sufficient.
   - Internet connectivity has been a huge challenge at all levels (National to LGA0.
   - During COVID it was a virtual training and even during RI implementation. The physical training has not given the desired results and is aggravated by including newer features.
   - The infrastructure for reporting (especially at the state and lower levels) is not very adequate; with the need for systems; the tablets provided are also not strong enough; there have been issues of stolen devices as well which have not been replaced.
   - Funding of data from OOP has been a huge challenge with delays in salaries being paid which is not unique to just the supply chain but to service delivery in general.
   - Political interference - with the change of governement, it affects the appointment of the CCOs, creating a huge gap in knowledge and competency; as seen in Sokoto, Kano, and Kebbi.
   - No major system issues have been identified. As a group (NLWG); we have been trying to support states through Liason officers to solve personnel issues generally.
   - States are supported by partners but the partners are not evenly distributed in providing support. The country dynamics also contribute in the balance of gender roles (more males in the north and more females in the south); There's higher usage on the male side than female; based on support provided generally speaking. If resources are equally distributed we should not see any differences based on gender. There's no serious gender analysis done, so we can't conclude.

7. What factors have facilitated the successful implementation and use of OpenLMIS?

   - The support from CHAI is in grant form (i.e. from Gavi) and they have supported the states with data plans; there is also some discussion on the support from UNICEF for data bundle funding but there are issues with dissemination of the funds, with respect to the choice of vendors/service providers. UNICEF also supports to ensure data quality on the OpenLMIS through the VSLs. UNICEF has also assisted some states for training and re-training on the OpenLMIS. There's a current discussion on training Kebbi CCOs; Katsina had been trained as well Kano, Jigawa and some others. UNICEF has assisted with refresher training in some states whilst others are in dire need. After the 1st rollout training, there hasn't been any training in the States. It's needed especially for the LGA CCOs.
   - The resources are not enough; aside from what the donor provides there are no funds from the government as of yet. However, there are ongoing discussions on at the ministerial level.

C. Perceptions on the link between OpenLMIS data use and immunization coverage

8. Have you noted any links between OpenLMIS data use and immunization coverage,

completion, and timeliness?

- Even before the OpenLMIS, using the stop-gap measure (ODK); the OpenLMIS has increased utilization over time. Ultimately when vaccines are available, it's expected that coverage increases. However, we have not been able to do this triangulation and also, last-mile delivery has not been achieved in its totality; but the OpenLMIS has helped to increase visibility to help us know where our vaccines are and would lead to increased coverage..

9. Has the use of the OpenLMIS led to any unexpected effects on vaccine delivery or staff roles? Describe these changes.
   - The introduction of the OpenLMIS introduced more roles and responsibilities in addition to already existing responsibilities.
   - The OpenLMIS has helped to spool reports and then helps us to investigate and call those responsible in a timely manner.

D. Learnings and recommendations from OpenLMIS utilization for COVID-19 vaccination

10. What unique lessons were learned (positive and negative) from using OpenLMIS for COVID-19 vaccination, and what recommendations do you have based on these lessons that can be applied to future large-scale campaigns and outbreak response in Nigeria or other countries?
    - It has created larger visibility to prevent under stock and over stock scenarios. You can track expiry or near expiry issues by batch numbers, etc.
    - It has helped to also manage vaccines.
    - Data archiving, BIN tracking, are some of the features that have been asked already amongst others.
    - Infrastructure at the lower levels needs to be improved. Refresher training at lower and national levels need to be looked into. National-level staff may need to also have exposure to other country patterns of usage to gain and understand best-practices for the implementation of supply chain systems.

Participant No. 003
Location: FCT
Respondent's Gender: **Male**

A. OpenLMIS and its role within the EPI Programme and beyond

1. What was the rationale for the rollout process of OpenLMIS for COVID-19 vaccination and subsequently Routine Immunization (RI) implementation?

   - Nigeria did not have a web-based supply chain software; it started with SMT but was not internet based and had issues with using excel sheets; and then we had NICS/MICS; some of the target software were not adequate. OpenLMIS was introduced to have good supply chain software for Nigeria and thereby improve supply chain in Nigeria. There's no difference in how it's been used in COVID and RI other than in the nomenclature. Using during COVID helped to quickly understand its use for RI

2. Describe the role OpenLMIS currently plays within the EPI program.

- We use it on a daily basis, for recording arrivals and distributions. It covers for all transactions made from the national store. There's the COVID, RI, and Non-polio and SIA modules; and then the commodities module. Although they are just 3 being used currently.
- OpenLMIS is relied upon for all activities, it is used for decision-making; checking for the balance; forecasting processes at national and state levels.
- We liaise with the EOC for campaign planning; but we make use of the OpenLMIS for all data needs from the logistics perspective; with which the EOC then uses to plan.

3. How has OpenLMIS impacted your decision-making and program management?

   -

4. To what extent is the OpenLMIS interoperable with other information systems (DHIS2)?

   - For now, it's still been worked on; the app is being developed. We get our stock balances from the OpenLMIS and consumption from the DHIS2; its not currently interoperable

5. Is the OpenLMIS already being used in other health areas (i.e. for other health commodities)
   - The OpenLMIS is not being used for other commodities just yet. Although, the module exists.

B. Barriers and facilitators to adoption, data quality, and use of OpenLMIS

6. What are the main barriers to the adoption, use, and data quality within OpenLMIS?
   - Network issues, sometimes from the backend; maintenance interferes with activities
   - When creating a user, it takes a lot time to get it done.
   - The physical inventory issue is another; which is also cumbersome to do on the OpenLMIS with the several modules that need to be gone through.
   - Human resource - huge turnovers
   - Internet connectivity - lack of data for internet connection
   - Other modules need to be created for CCE, temperature monitoring, etc
   - Lack of interest; more capacity building is needed.
   - Contact support needs to be improved on as well.
   - There are some glitches in the system where a batch's expiry date returns wrong.
   - At the lower levels (LGA), these are where most connectivity issues are, due to the area of connectivity.
   - CHAI is helping with capacity building.
   - There are no gender-related differences that have been noticed.
   - There are fewer challenges now compared to when we were just starting. The system is now more user-friendly.
   - Kano has really improved over time; Nasarrawa, Ebonyi and Lagos have been consistent; fewer LGAs are now problematic.
   - There's a constant follow-up that has helped with improving the use of the OpenLMIS.

7. What factors have facilitated the successful implementation and use of OpenLMIS?
   - UNICEF at a point was providing data and continue to provide other forms of support;

with CHAI and Gavi spearheading the support.
- Training was done about a year ago; more recent trainings are on-the-job training. However, CHAI provides weekly support calls to followup on the use of the system.
- Additional resources should be provided to fund data acquisition, especially at the lower levels; and then expansion of the system to be more robust and interoperable with DHIS2.
- OpenLMIS should be a one-stop-shop to improve traceability, resources should be geared towards having scanning of products for registration.
- Funds should be geared towards creating a call center; where all the Admins are available in a single place to address issues across the locations.

C. Perceptions on the link between OpenLMIS data use and immunization coverage

8. Have you noted any links between OpenLMIS data use and immunization coverage, completion, and timeliness?
   - Coverages have to do with last mile delivery, we have not seen it entirely and that is more on the service delivery side of things.

9. Has the use of the OpenLMIS led to any unexpected effects on vaccine delivery or staff roles? Describe these changes.
   - It hasn’t led to any unexpected changes; the system is still been tested.

D. Learnings and recommendations from OpenLMIS utilization for COVID-19 vaccination

10. What unique lessons were learned (positive and negative) from using OpenLMIS for COVID-19 vaccination, and what recommendations do you have based on these lessons that can be applied to future large-scale campaigns and outbreak response in Nigeria or other countries?
    - The OpenLMIS has shown the importance of being proactive at any point in time. It helps improve the supply chain's visibility and has led to increased accountability.
    - There were issues during the COVID-era which helped to transition to RI more comfortably.
    - The modules are okay. Temperature monitoring and CCE maintenance should be improved on. In Turkey, all the CCE (stores and vehicles) are linked to one system and it’s easy to see centrally; this needs to be replicated in Nigeria, however resource-intensive. When using SMT, all the equipments were linked together and was just a excel tool.

Participant No. 004
Location: FCT
Respondent’s Gender: <u>Male</u>

A. OpenLMIS and its role within the EPI Programme and beyond

1. What was the rationale for the rollout process of OpenLMIS for COVID-19 vaccination and subsequently Routine Immunization (RI) implementation?

   - It became apparent to roll out for improved visibility. During COVID, it was urgent and was less technical than RI implementation. The approval was first given for COVID-19

before the development of the RI modules within the OpenLMIS.

2. Describe the role OpenLMIS currently plays within the EPI program.

    - The major role has been the visibility of the stock status across the supply chain, from national to LGA level.
    - We are able to see stock levels and it helps to make decisions on the distribution of vaccines.
    - There are several modules in the OpenLMIS that help for RI and Campaigns alike; we use the OpenLMIS to monitor levels and move stocks between programs for efficiency.

3. How has OpenLMIS impacted your decision-making and program management?
    - The OpenLMIS helps to understand whats available at the state and helps to see those states that are at critical need and then helps prioritize distribution.

4. To what extent is the OpenLMIS interoperable with other information systems (DHIS2)?
    - Currently, there's work ongoing to have it interoperable; current triangulation is done manually using Tri 360.

5. Is the OpenLMIS already being used in other health areas (i.e. for other health commodities)
    - There's some progress on that but it's currently not being used, although the modules already exist.

B. Barriers and facilitators to adoption, data quality, and use of OpenLMIS

6. What are the main barriers to the adoption, use, and data quality within OpenLMIS?
    - Lack of data (internet connectivity) at the lower levels (State and LGA levels).
    - New staff that need to be trained.
    - Nasarawa, Yobe, Bayelsa, are some of the states that have been consistent.
    - I have not really looked into any gender disparities.
    - There are no much challenges personally.

7. What factors have facilitated the successful implementation and use of OpenLMIS?

    - CHAI and UNICEF; although CHAI has played a major role in training support.
    - There have been online centers to train and solve issues as the need arises.

C. Perceptions on the link between OpenLMIS data use and immunization coverage

8. Have you noted any links between OpenLMIS data use and immunization coverage, completion, and timeliness?
    - Through the OpenLMIS, there has been improved visibility to make sure that there's timely replenishment for activities to continue.

9. Has the use of the OpenLMIS led to any unexpected effects on vaccine delivery or staff roles? Describe these changes.
    -

D. **Learnings and recommendations from OpenLMIS utilization for COVID-19 vaccination**

10. What unique lessons were learned (positive and negative) from using OpenLMIS for COVID-19 vaccination, and what recommendations do you have based on these lessons that can be applied to future large-scale campaigns and outbreak response in Nigeria or other countries?
    - The OpenLMIS helps with visibility and flagging inconsistencies, which wasn't always the case.
    - The more features are added, the more it gets complicated; the idea is to make it simpler and adding more features at this time might cause more harm than good.
    - If the VVM status can be giving alerts and also those that are close to expiry; it would help to improve the decision-making.

Participant No. 005
Location: FCT
Respondent's Gender: **Male**

A. **OpenLMIS and its role within the EPI Programme and beyond**

1. What was the rationale for the rollout process of OpenLMIS for COVID-19 vaccination and subsequently Routine Immunization (RI) implementation?

    -

2. Describe the role OpenLMIS currently plays within the EPI program.

    - Still undergoing training, however the OpenLMIS is used for the distribution of vaccines to the last mile. It's been fine and working well and others have been assigned, specifically; Mr. Liman and Mr. George.

3. How has OpenLMIS impacted your decision-making and program management?
    - The OpenLMIS data is being used every Wednesday for meetings to discuss stock issues. The OpenLMIS is the major tool that's being used.

4. To what extent is the OpenLMIS interoperable with other information systems (DHIS2)?
    -

5. Is the OpenLMIS already being used in other health areas (i.e. for other health commodities)
    - .

B. **Barriers and facilitators to adoption, data quality, and use of OpenLMIS**

6. What are the main barriers to the adoption, use, and data quality within OpenLMIS?
    - Network and manpower have been major issues that have affected adoption. More people need to be trained to be able to use the tool properly. internal trainings are being planned to ensure everyone within the Operations unit is capable of using the OpenLMIS.

7. What factors have facilitated the successful implementation and use of OpenLMIS?

- .

C. Perceptions on the link between OpenLMIS data use and immunization coverage

8. Have you noted any links between OpenLMIS data use and immunization coverage, completion, and timeliness?
   - .

9. Has the use of the OpenLMIS led to any unexpected effects on vaccine delivery or staff roles? Describe these changes.
   - The OpenLMIS is working well and is being used well; previously we were using NICS/MICS.

D. Learnings and recommendations from OpenLMIS utilization for COVID-19 vaccination

10. What unique lessons were learned (positive and negative) from using OpenLMIS for COVID-19 vaccination, and what recommendations do you have based on these lessons that can be applied to future large-scale campaigns and outbreak response in Nigeria or other countries?
    - Nil

Participant No. 006
Location: FCT
Respondent's Gender: **Male**

A. OpenLMIS and its role within the EPI Programme and beyond

1. What was the rationale for the rollout process of OpenLMIS for COVID-19 vaccination and subsequently Routine Immunization (RI) implementation?

   -

2. Describe the role OpenLMIS currently plays within the EPI program.

   - The OpenLMIS will go a long way to help with last-mile delivery.

3. How has OpenLMIS impacted your decision-making and program management?

   -

4. To what extent is the OpenLMIS interoperable with other information systems (DHIS2)?

   -

5. Is the OpenLMIS already being used in other health areas (i.e. for other health commodities)
   -

B. Barriers and facilitators to adoption, data quality, and use of OpenLMIS

6. What are the main barriers to the adoption, use, and data quality within OpenLMIS?
   - At the national; we need the documentation of the OpenLMIS to correspond with other store management tools.
   - The current training is about OpenLMIS and the focus is currently to improve zeal in using the system.
   - I believe that the training will help to improve my role, especially in vaccine arrivals.
   - There's no formal training currently and what is currently going on is being done at a convenience

7. What factors have facilitated the successful implementation and use of OpenLMIS?
   - .

C. Perceptions on the link between OpenLMIS data use and immunization coverage

8. Have you noted any links between OpenLMIS data use and immunization coverage, completion, and timeliness?
   - .

9. Has the use of the OpenLMIS led to any unexpected effects on vaccine delivery or staff roles? Describe these changes.
   - .

D. Learnings and recommendations from OpenLMIS utilization for COVID-19 vaccination

10. What unique lessons were learned (positive and negative) from using OpenLMIS for COVID-19 vaccination, and what recommendations do you have based on these lessons that can be applied to future large-scale campaigns and outbreak response in Nigeria or other countries?
    - .

Participant No. 007 / 008
Location: FCT
Respondent's Gender: **Male☐/ Male☐**

A. OpenLMIS and its role within the EPI Programme and beyond

1. What was the rationale for the rollout process of OpenLMIS for COVID-19 vaccination and subsequently Routine Immunization (RI) implementation?

   - To track utilization of vaccines from the supply to the last-mile, by batch and expiry. Initially, we have been using the NICS/MICS which was comfortable. With the introduction of the OpenLMIS, it's been enjoyed. The OpenLMIS is almost similar to the previous tool. Although, with the OpenLMIS one has to enter the batch number which creates an issue with the
   - I've not fully used the OpenLMIS, I've not been taking part in OpenLMIS issues. It's the

same implementation since COVID-19 and now with RI.

2. Describe the role OpenLMIS currently plays within the EPI program.

   - Through the issuance and tracking of utilization of vaccines from the national to the last-mile.
   - Every month, there's a dashboard and allocation data from the OpenLMIS; Vaccines are issued based on the data provided from lower levels.
   - OpenLMIS is used for both RI and SIA and to monitor it across the entire supply chain.

3. How has OpenLMIS impacted your decision-making and program management?

   - The use of the OpenLMIS helps to see the utilization of states and helps with distribution decisions.
   - This data is used weekly.

4. To what extent is the OpenLMIS interoperable with other information systems (DHIS2)?

   -

5. Is the OpenLMIS already being used in other health areas (i.e. for other health commodities)
   -

B. Barriers and facilitators to adoption, data quality, and use of OpenLMIS

6. What are the main barriers to the adoption, use, and data quality within OpenLMIS?
   - Internet connectivity and security challenges are major issues that have hindered the use of the OpenLMIS; about 1,000 naira was been provided at some point but
   - Nasarawa, Taraba, Adamawa, Jigawa are some of the high reporting states and can be attributed to their passion on the management level; they look at their data on a weekly basis. Even the ES is always asking for feedback on the use of the OpenLMIS.
   - There are no gender differences attributed to performance in the use of the OpenLMIS
   - CHAI and UNICEF have been the support organizations on the use of the OpenLMIS
   - There have been trainings at the NSCS, and the plan is to have this on a weekly basis to have someone from CHAI asides the in-house personnel
   - The resources are considered enough asides the issues of data for internet connectivity.

7. What factors have facilitated the successful implementation and use of OpenLMIS?
   - .

C. Perceptions on the link between OpenLMIS data use and immunization coverage

8. Have you noted any links between OpenLMIS data use and immunization coverage, completion, and timeliness?
   - Yes; when we have more interoperability between systems we believe there'd be increase in reaching the last mile delivery.

9. Has the use of the OpenLMIS led to any unexpected effects on vaccine delivery or staff

roles? Describe these changes.

- The OpenLMIS has improved visibility and also response time; such that once there is a critical level we are able to immediately check and troubleshoot.
- Recently we retrieved bOPV from a state because it was overstocked and it helped to inform redistribution to other states that need the vaccines.

D. Learnings and recommendations from OpenLMIS utilization for COVID-19 vaccination

10. What unique lessons were learned (positive and negative) from using OpenLMIS for COVID-19 vaccination, and what recommendations do you have based on these lessons that can be applied to future large-scale campaigns and outbreak response in Nigeria or other countries?

- The OpenLMIS has helped facilitate the use of data for decision-making (distribution, re-distribution, etc)
- One of the features that we need is to have the OpenLMIS generate packing list/batch numbers automatically; instead of having to enter it manually on the system.

Participant No. 009
Location: FCT
Respondent's Gender: **Male**

A. OpenLMIS and its role within the EPI Programme and beyond

1. What was the rationale for the rollout process of OpenLMIS for COVID-19 vaccination and subsequently Routine Immunization (RI) implementation?

-

2. Describe the role OpenLMIS currently plays within the EPI program.

- OpenLMIS has been very impactful but is still a work-in progress; it provides stock reports in real-time across the supply chain (National to LGA level) as well as the VVM status, expiry dates, quantities available etc; and these information is used for planning at the national allocation meetings. It's a welcome development.
- I visit the OpenLMIS at least 2ce a day; to see where the vaccines are and the status/situation of the vaccines, also in terms of the quantities available; and then I use the data to advise the national strategic cold store on what to do concerning the findings we see within the OpenLMIS.
- There are reports that we get from colleagues at the SLWG and at the ZLWG level showing the physical count stock and this is compared to what is on the OpenLMIS to ensure the reliability and accuracy of the data on the OpenLMIS. Also, the data from the OpenLMIS helps us to respond to critical stock issue, including addressing expiry of vaccines; VVM stages, etc
- The OpenLMIS is going to reduce some workload and reduce the need to jump through several tools as it was in the past.

3. How has OpenLMIS impacted your decision-making and program management?

-

4. To what extent is the OpenLMIS interoperable with other information systems (DHIS2)?
    - It's still a work in progress, however, governance has declared interest in ensuring interoperability. There have been several meetings as well to address this.

5. Is the OpenLMIS already being used in other health areas (i.e. for other health commodities)
    - There are plans to incorporate other commodities; but isn't currently used for other commodities. The ED has instructed through the Dir. Logistics to have a more robust system to accommodate other health commodities.
    - The OpenLMIS is also used for CCE management/functionality as well, although other tools are been used for CCE and not solely the OpenLMIS.

B. Barriers and facilitators to adoption, data quality, and use of OpenLMIS

6. What are the main barriers to the adoption, use, and data quality within OpenLMIS?
    - Most of the barriers have been addressed except the following:
    - Mobile data at state and LGA level lacks support. UNICEF is also looking into supporting more for this.
    - Attitudinally, there's some laxity in the utilization of the tool at the LGA level; some of the data on the OpenLMIS although it's meant to be real-time, some LGAs have their latest data dated 2-3 months prior.
    - The attitude at the LGA level is a primary concern and needs to be addressed, and then the issue of the data for internet connectivity would be secondary. There are states that even with the provision of infrastructure; seldom use the OpenLMIS. Even with close supervision, they provide excuses. At some levels, although very few, they make use of the tablets for other purposes other than the OpenLMIS.
    - The proportion of people that have not used the OpenLMIS is information that can be provided by the NPHCDA.
    - We haven't seen any gender differences
    - In the North, we have more males than females employed for the work and the opposite is the case in the south
    - Everyone works as a team and that has been helpful.

7. What factors have facilitated the successful implementation and use of OpenLMIS?
    - Constant tracking by the NLWG, and displaying the level of performance on scorecards has helped to improve utilization.
    - Triangulating the OpenLMIS data with the physical stock count on a routine basis and following up with the different personnel, helps to enforce the utilization of the platform.
    - It might be difficult to really decipher what the main barrier is until data (for internet connectivity) is provided constantly and consistently as well as ensuring the technical know-how. Although, there's a platform that provides support once issues are raised.
    - After the introduction of the OpenLMIS, CHAI team was providing data for some months and UNICEF also advocated for the provision of data, although through a vendor, to accommodate UNICEF policies (which do not allow for direct supply of funds for airtime/data but through vendors).
    - Training started at the national level first and then all SCCO were brought into Abuja as

well as some State Logistics Officers (SLOs) and SIOs; at that level, we asked the state to select one person per location as the focal person.
- Training was done on utilization; stock management (recieving and distribution of vaccines, adjustments

C. Perceptions on the link between OpenLMIS data use and immunization coverage

8. Have you noted any links between OpenLMIS data use and immunization coverage, completion, and timeliness?
   - Yes, there are a lot of links; OpenLMIS gives us when states have vaccines or not; clearly, where there's no sufficient vaccine, then there'd be less children being vaccinated. Colleagues are also tracking the system to confirm

9. Has the use of the OpenLMIS led to any unexpected effects on vaccine delivery or staff roles? Describe these changes.
   - Yes, the OpenLMIS has helped out; the laxity was worse before the advent of the OpenLMIS; stock-on hand was hard to see and get the actual figures. The SLWG work together to ensure to hold the responsible persons accountable.

D. Learnings and recommendations from OpenLMIS utilization for COVID-19 vaccination

10. What unique lessons were learned (positive and negative) from using OpenLMIS for COVID-19 vaccination, and what recommendations do you have based on these lessons that can be applied to future large-scale campaigns and outbreak response in Nigeria or other countries?
    - Wasn't involved in the COVID implementation; as a pilot the retrieval and redistribution process during COVID helped with the RI implementation.
    - There were challenges in times past but have been resolved.
    - OpenLMIS report is real-time but does not archive previous reports for the previous weeks. It's impossible to get data of what happened in previous weeks.
    - OpenLMIS tells when an individual uses the platform last, but when the stocks are not updated over weeks or months; the last updated stock (although obsolete) still shows up. It will be better that the data point is deleted as it does not present a true picture of the current realities; or it should say "**Not Updated**" to prevent the download or analysis of data that is not a true picture of the current realities.

Participant No. 010
Location: FCT
Respondent's Gender: <u>Male</u>

A. OpenLMIS and its role within the EPI Programme and beyond

1. What was the rationale for the rollout process of OpenLMIS for COVID-19 vaccination and subsequently Routine Immunization (RI) implementation?

   - There's no difference between how the system is used for COVID and RI
   - Nigeria in the past has gone through different eLMIS; in 2015, CHAI rolled out a VSPM Excel-based system to see stock levels at the National and State levels; and then Microsoft NAVISION which was complex for the users.

- NPHCDA worked with Field Intelligence to developed NISC-MIS was also developed in 2018 and in 2019/2020, it was discontinued because of high licensing fees which led back to the use of ODK as a stop-gap reporting tool through SMS; which only helped to report stock levels on the weekends.
- To avoid the trend of changing eLMIS tools and paying lots of money; the COVID roll out was an opportunity for Gavi to roll out a tool that's off the shelf and open-source.
- The rollout for COVID was for a learning curve and also bearing in mind that there had been a Gavi audit that clearly showed that in Nigeria, the lack of an eLMIS led to less accountability and less visibility.
- It was a successful roll-out which led to Gavi releasing an RFP for partners to bid for the RI roll-out of the OpenLMIS.

2. Describe the role OpenLMIS currently plays within the EPI program.

    - Giving visibility to the stock levels across the stores
    - There are over 435 cold stores across the 5 of 6 levels of the supply chain; National, Zonal, State, Satellite and LGA stores.
    - It provides the data for decision making across the levels and invariably at the last-mile.
    - It informs the distribution of vaccines with the allocation reports
    - Provides the information for vaccine management, and procurement to the National team for decision-making
    - The OpenLMIS is presented at the NLWG meetings every Wednesday
    - On Fridays the vaccine stock performance monitoring dashboard from the OpenLMIS is presented to the team.
    - Every member of the NLWG is assigned to a zone for follow-up and to deal with mismatches at the states.
    - Every issue around stock expirt/near expiry; mismatches between vaccine and diluents are flagged and the liaisons follow up on these issues.
    - On stock availability, the NLWG takes decisions wrt procurements; for example, this week theres a low-stock of Rota and HepB; which has informed a request for cost-estimates on those quantities of vaccines.

3. How has OpenLMIS impacted your decision-making and program management?

    -

4. To what extent is the OpenLMIS interoperable with other information systems (DHIS2)?

    - OpenLMIS can interoperate with any other system; the government is currently building an interoperability layer for this to link the OpenLMIS to the DHIS2 and the existing EMID app. There's a need for a stock management tool at the facility level for last-mile visibility and the requirement document for the eLMIS at the facility level has been approved by the NPHCDA and the eLMIS is being developed by eHealth and HISP. CHAI will then ensure it's interoperability to link all levels of the supply chain to the last-mile.

5. Is the OpenLMIS already being used in other health areas (i.e. for other health commodities)
    - Other health areas currently use different tools; Malaria and some other areas (FP) are using the NHLMIS; the reason is around the fragmentation on how these commodities are been purchased and distributed. The government will have to make that decision if

other commodities are to be used on the OpenLMIS.

B. Barriers and facilitators to adoption, data quality, and use of OpenLMIS

6. What are the main barriers to the adoption, use, and data quality within OpenLMIS?

- Infrastructure was a major barrier to adoption; there was a lack of mobile tablets and computers; Gavi then procured 1,000 devices (laptops/tablets) and were distributed to the State and LGAs;
- Mobile data was also another barrier and the quality of data services; to mitigate this; each officer had to identify the best network to use.
- The provision of monies for data purchases is a current barrier to the use of the OpenLMIS and is also a gap that Gavi has tried to mitigate and the government is looking into assisting as well.
- The states will have to also step in for sustainability as some states already have.
- The capacity across the supply chain is different, not necessarily on the path of the individual; the OpenLMIS is the first to be used at the LGA level but this has been mitigated by the bi-weekly calls to discuss issues and topics relating to the OpenLMIS; there are also on-the-job support and provision of video materials to improve their knowledge on the use of the tool.
- The OpenLMIS operations room is run by the members of the ICT team and the logistics department at the NPHCDA. The operations room have channels on WhatsApp and on the tool itself, through tickets which CHAI supports resolving. The calls are made bi-weekly/fortnightly; at the moment the help desk is having those calls on Tuesdays and Wednesdays every week till the end of the year, when there'd be a handover to the government entirely.
- There haven't been any deliberate gender assessment, so it's hard to speak on it.

7. What factors have facilitated the successful implementation and use of OpenLMIS?

- For data quality, We adopted a 5-pronged strategy - 1. Triangulation of data on openLMIS with physical counts at the facilities every week. 2. Quantities of mismatches to give indication of poor practices of distribution 3. Utilization rate of the OpenLMIS ; 4. The scorecard is also used to compare with other stores; where the highest store depicts the highest performer (best quality data)
- Virtual calls have helped for adoption and is done every month; in conjunction with the weekly help desk calls.
- There's also the hands-on training that is conducted.
- Spot checks have also helped.
- Providing data
- Training is done on a monthly basis; but isn't a retraining of the workforce, rather is an avenue for CCO to get retrained every month
- The weekly help-desk provides training for 2 days in a week; after the first training (i.e after the roll-out for COVID) there have been changes in personnel and the national sends people to train them.
- There are also targeted trainings in Kebbi and Kaduna, where there has been poor utilization of the tool.
- The NLWG has put in their workplan with funding request from Gavi to fund another nation-wide physical training.
- As regards the resources needed; States need to provide mobile data for personnel.
- The server currently is being paid by Gavi; the country needs to provide more resources

into that. The re-training resources have been budgeted and costed and is awaiting approval.

C. Perceptions on the link between OpenLMIS data use and immunization coverage

8. Have you noted any links between OpenLMIS data use and immunization coverage, completion, and timeliness?
   - Coverage is dependent on a lot of factors one of which is the availability of vaccines; What OpenLMIS does is to inform you on the stock-levels to help you provide enough vaccines for effective coverage.
   - The level of stock-outs is also something that OpenLMIS provides in real-time unlike the DHIS2 which is provided post-reporting timeline.
   - Coverages are better, but cannot be totally attributed to the openLMIS alone.

9. Has the use of the OpenLMIS led to any unexpected effects on vaccine delivery or staff roles? Describe these changes.
   - The practice of calling the lower levels to get stock levels before distribution has stopped;
   - The pattern of reporting and use of data has changed dramatically as a result of the use of the OpenLMIS.

D. Learnings and recommendations from OpenLMIS utilization for COVID-19 vaccination

10. What unique lessons were learned (positive and negative) from using OpenLMIS for COVID-19 vaccination, and what recommendations do you have based on these lessons that can be applied to future large-scale campaigns and outbreak response in Nigeria or other countries?
   - From the COVID vaccination, because of the pandemic and the need for a quick roll-out; the initial training was done virtually. An eLMIS tool is not one that requires a virtual roll-out as post the roll-out, the utilization was poor, until when physical training was done.
   - The need for infrastructure at the state stores. It was an eye-opener to find out that they don't have the needed infrastructure despite the resources sent to that level. There's a list of lessons learned across the different phases that can be shared.
   - The NLWG has listed and costed newer features; one of which is the mobile application that would enable offline capturing and on mobile phones as opposed to using only browsers on computers or tablets.
   - Vendor management to manage distribution, and compare schedules and quantities distributed versus what is received by the CCO. Also the introduction of other commodities.

Participant No. 011
Location: FCT
Respondent's Gender: **Female**

A. OpenLMIS and its role within the EPI Programme and beyond

1. **What was the rationale for the rollout process of OpenLMIS for COVID-19 vaccination and subsequently Routine Immunization (RI) implementation?**

- We have had some LMIS in the past, NICS/MIS but there was no visibility at the LGAs; the OpenLMIS is one of a kind as it provides more visibility. OpenLMIS is also helping to minimize stockout and overstock events. The OpenLMIS is a good management tool for vaccines and devices; it has all the programs; starting with the COVID module and now RI and Polio SIA; unlike other LMIS. It's linked with DHIS and is now accessible.
- There are no differences between its use for Covid and RI programs.

2. **Describe the role OpenLMIS currently plays within the EPI program.**

- We usually have SERICC meetings where I present data from the OpenLMIS; the tool helps to inform decisions. During our RI monthly meetings, the CCO also present findings from the OpenLMIS (including wastage data). We use the OpenLMIS as needed; we distribute once in a week for distribution. When there's no distribution, we also do physical inventory every week and can be any part of the week; we also do monthly stock meetings.
- We rely on the OpenLMIS for planning and monitoring of activities.

3. **How has OpenLMIS impacted your decision-making and program management?**

- It gives me visibility and helps to make informed decisions. Addressing stock levels, etc.

4. **What factors have facilitated the successful implementation and use of OpenLMIS?**
- The factor that facilitated the successful implementation is just dedication to make sure that things are done well by us and the support from CHAI, NPHCDA and UNICEF. Strategies on the training and retraining of personnels by me and UNICEF. Concerning funding, CHAI, NPHCDA, GAVI and UNICEF do provide fundings. There is no support from government and I think the government should render financial support.

-

B. Barriers and facilitators to adoption, data quality, and use of OpenLMIS

6. What are the main barriers to the adoption, use, and data quality within OpenLMIS?
- When the roll-out came; there was no support for data provision and the OpenLMIS is data intensive; CHAI however provided support for this at the LGA level and also

provided training and re-training. CHAI also provides virtual support and training. However, the major issues are now as regards the provision of internet services. CHAI has indicated that the government is to take over.

- The OpenLMIS is also not so easy to use. There's no feature to undo mistakes that were made especially when someone makes errors.
- There's been no limitation from the gender perspective.

7. What factors have facilitated the successful implementation and use of OpenLMIS?

- We are very dedicated to the work and self-motivation has been a great factor; we have support from CHAI and NPHCDA.
- Training and re-training has been the major strategy to improving the use of the OpenLMIS.
- NPHCDA, Gavi, CHAI are the main organizations providing support, as well as UNICEF through the VSLs.
- I understand what the partners say when they said that states should take ownership; there's no support from the government and having people using their own funds for data is not ideal nort motivating; each LGA has tablets and laptops at the state level.

C. Perceptions on the link between OpenLMIS data use and immunization coverage

8. Have you noted any links between OpenLMIS data use and immunization coverage, completion, and timeliness?

- There's a link with DHIS2, and we can see the information on the DHIS2. Once your coverage is low, it means you are not meeting targets. For every antigen, there's an allowed wastage rate.
- Our monthly RI meeting speaks to the reporting completeness.
- NPHCDA has not slept on immunization reporting; however, the OpenLMIS has helped with improved visibility.

9. Has the use of the OpenLMIS led to any unexpected effects on vaccine delivery or staff roles? Describe these changes.

- We receive vaccines quarterly, but sometimes it's rationed. The openLMIS helped to prompt emergency deliveries when needed. It also helped to push vaccines to LGAs as well.

- The OpenLMIS increased our workload, and the inability to undo certain entries leads to increased data entry work. However, it also has helped to see errors and rectify them timely.

D. Learnings and recommendations from OpenLMIS utilization for COVID-19 vaccination

10. What unique lessons were learned (positive and negative) from using OpenLMIS for COVID-19 vaccination, and what recommendations do you have based on these lessons that can be applied to future large-scale campaigns and outbreak response in Nigeria or other countries?

- It was not very easy to use at first, but after spending time it becomes easier.
- They also keep adding some features to improve the system that have made it more user-friendly.
- They should think about making room to easily rectify errors and undo mistakes made.
- Initially, the COVID module was done for over a year first; and the training was done virtually with constant support. At the state level, it was easily done and at the LGA level it was gradually implemented.

Participant No. 012
Location: FCT
Respondent's Gender: Female

A. The stock management system and flow of data from paper-based to OpenLMIS

1. Describe your role and the stock management system you currently use for vaccine stocks

- The stock management we currently use is both paper-based and electronic based; which is the Nigerian OpenLMIS with which we receive vaccines, we monitor the expiration date, VVM, and we also issue out the vaccines using the OpenLMIS. Then their utilization is also reported using the DHIS from the Area council back to the state level.

2. Describe the flow of data from health facilities to higher levels of the supply management system.

- The flow of data now is using the VM1 A and B at the Health facility level, they use it to collect both vaccine utilization, doses received, doses opened, and balance at the end of the month, they also use immunization summary sheet to collate the immunization conducted, then the two data is sent to the area council where the Rido, M&E and LIO capture the information on DHIS.
- But when we talk about vaccine stock, they use VM 1 A and B and the stock is sent back

to the area council level where they use VM 3 to collect it and they also send it back using the DHIS.

- But when you talk of OpenLMIS, it only ends at the area council (i.e. LGA), it does not extend to the health facility level, so only the area council receive vaccine from us using OpenLMIS, they distribute to the health facilities using OpenLMIS, but the health facilities do not operate OpenLMIS.
- There is tendency that discrepancies can occur if there is close vial wastage in the case of expiration or change of VVM or breakage, but we usually fish them out during physical stock count which we do weekly. So if there is any disparity, those gaps are closed before we report weekly.

B. OpenLMIS user experience, benefits, enablers and barriers

3. What are the main benefits of using OpenLMIS, compared to the paper-based system?

- The major benefit I see is data at hand. As soon as you need it, you can access it because its web-based, so as long as you are with your system or with your phone, you can access it. (you can get the exact condition of the stock where ever you are, not necessarily having to be in the store. This advantage is over the paper-based that needs you to be in the store before you can confirm your stocks)
- The OpenLMIS has improved our work in terms of making data available and handy. From the OpenLMIS you can view the vaccine availability in the area council, even when the officers are not ready to respond timely (it's just that it takes time to be scrolling through their records). Then on our own part, we can view the vaccines that are about to expire at a glance (based on what you have enter anyway) and the VVM status, thus enabling us to prioritize vaccine distribution based on these conditions (i.e. the expiry status and the VVM status)

4. How easy is OpenLMIS to use in your daily work?

- OpenLMIS is easy to use in our daily work but not always easy, especially when we have to deal with network. There are sometimes you want to immediately input and the network will not be friendly and that will cause delay, but when the network is ok, it's very easy; (as we are distributing, we are updating the OpenLMIS and sometimes we usually need to open the system for us to see those vaccines that have earlier expiration date before we actually do the distribution, so it is very very useful.
- I am not certain if there is any task that is difficult to do the OpenLMIS because I am only tasked with checking the information, and using it to make decision while my officers (the ones that handle the OpenLMIS) do the inputting of the data.
- It blends well into my day to day activities; as we find it easy to use for the confirmation of vaccine expiration date, because once you open the pages, it shows you the vaccines that are about to expire, and then you have to take those ones out first, but if you are not using the OpenLMIS and you rely on the paper work, what if there is a mistake in the paper work, you will still miss out, but the OpenLMIS gives us exactly what is on the vaccine, so we can match the batch number and the expiry date.

5. Are there any features of OpenLMIS you find particularly helpful or unhelpful?

- All the features are ok, they are all okay,
- There is none of the feature that is not helpful.
- Features to be included; I would have loved a situation where the vaccine that have been used are no more displayed in the list, because, this makes the page bulky and difficult to find the particular information you want to work with. Delete the batch numbers that are expired. Delete the vaccines that are no longer in use. So when you open a page, you see plenty zero zero (i.e., the stock level is zero), such should be wiped out of the system.
- There is another feature they promised us where when you are conducting physical stock, it will only show you the quantity available based on what your OpenLMIS is showing, you will now cross check if it is okay, you will tick it, but if it’s not ok, you will then know how to update those ones. Those things will make the OpenLMIS more user friendly.
- Reminder and alerts: I used to get a reminder every Friday; particularly once any antigen expiration date has been exhausted in the OpenLMIS, it will send me a message requiring me to go back to the system and check and then do the needful

6. What factors have enabled you to effectively use OpenLMIS in your work?
   - Individually: The factors are the provision of device for it. A laptop used by my SLO and other devices for the people helping us with the work.
   - At our level here, the interruption in the network is not as much as for my subordinates at the LGAs. They are in the bush, so they usually encounter issues. But for us here, except when the weather is not friendly or when the network provider has issues. But overall, once you have data, it loads.
   - Organizational; the VSL is always supporting, once you contact him, he helps. The SLO told me that she raised a memo to the ES for the board to support with data even up to the LGA level.
   - Training: during the initial onboarding, (that is when OpenLMIS was meant for Covid), I was trained with the State Logistic Officer (SLO), but later when it was converted to RI Module, the SLO was the one that went in for the long training package. Then after that, there are some refresher courses I partook of.
   - The trainings are not regular, it was one-off. But there is the OpenLMIS platform in the FCT; they usually schedule some days, like yesterday and today, they will open zoom to offer assistance to anyone who is facing any problem using the system.
   - The training is from NPHCDA (Loretta)
   - I don’t think there are enough resources directed towards the OpenLMIS, we need to be supported, if you want somebody to work very well, it’s good you give the person what s/he need to be effective at work. There was a time they paid us for data from national, about 2021, but after that, I have not seen any other payment for data, each time I complain, they will say I should take from my personal purse. Which is even the main reason why I don’t want to put myself in… because for my volunteer, if I ask, they will quickly support her, but for you that is a worker, they expect that you use your personal funds, but you also have other personal responsibilities, how much then do you take home at the end of the day. If they can map out a certain… even my LGA CCOs, once you call them, they will complain about data, because network is not too friendly with them; some of them will tell me that they will input data, the thing will continue to roll (I also experience this, if I put 1.5gb, I cannot get anything, the money will finish and I will not get what I want) before it will show you the page that you want, and if you ever make a mistake that requires you to go back, the process will repeat itself. For example, the CCO at Kwali, He has to leave Kwali to either Gwagwalada for him to use the OpenLMIS; there is no network in Kwali,and when He gets to Gwagwalada and the

system starts to roll and roll, He will be disappointed. Sometimes He has to go to Abaji, and it's not that it is answering immediately.
- I think the people who are in charge value it because they know the benefit of the app.
- Equipment is provided by NPHCDA, Data is provided by the state,
- UNICEF usually supports
- I have not seen any equipment maintained.

7. What challenges have you experienced with using OpenLMIS?

   - Challenges; Network, funding for data, the application takes time to load, it is slow, out-of-stock batches and expired vaccines remain on the page making it difficult to access necessary information
   - we need more refresher training, because this is having to do with technology, and you know most of us are not trained to use technology.

C. Recommendations and improvement

8. What improvements would you suggest to make OpenLMIS more effective for future mass vaccination campaigns as well as routine immunization?

- I would have loved a situation where the vaccine that have been used are no more displayed in the list, because, this makes the page bulky and difficult to find the particular information you want to work with. Delete the batch numbers that are expired. Delete the vaccines that are no longer in use. So when you open a page, you see plenty zero zero (i.e., the stock level is zero), such should be wiped out of the system.
- There is another feature they promised us where when you are conducting physical stock, it will only show you the quantity available based on what your OpenLMIS is showing, you will now cross check if it is okay, you will tick it, but if it's not ok, you will then know how to update those ones. Those things will make the OpenLMIS more user friendly.
- Like in NISC-MIS, we see vaccines that will soon expires denoted with red color, also, it usually arranges vaccines batch by batch, the one that will expire soon will be at the top while the one that will expire last will be down. It sorts according to expiration date.

FCT (21)

Gender: Male

Introduction

I am an assistant CCO, Appo resettlement, I receive vaccine, check and remove broken ones, and prepare report.

A. The stock management system and flow of data from paper-based to OpenLMIS

1. **Describe your role and the stock management system you currently use for vaccine**

stocks

- I receive vaccines and issue them to the appropriate facilities for campaign vaccination and immunization. I use the OpenLMIS and the paper-based system for vaccine, data and assets management.

2. **Describe the flow of data from health facilities to higher levels of the supply management system.**

- I receive stock from the state and distribute to the communities' facilities, using both OpenLMIS and the paper-base. Yes, sometimes there are discrepancies in the recording, but, before the report is sent, I ratify it using both the OpenLMIS and the paper-based before sending the report out. The problem has always been in stock balances.

B. OpenLMIS user experience, benefits, enablers and barriers

3. **What are the main benefits of using OpenLMIS, compared to the paper-based system?**

- In using the OpenLMIS, I have to record all the stock received and return to the paper-base to repeat the same thing, the work is just too much for one person. OpenLMIS is good, but sometimes network disturbs it so much and that leads to using the paper-based system alone. In fact, since this year, the Tablet given to me has not been opening and I don't use it again, I'd have to go to business centres to do the data entry and that is just not convenient. It has not improved my work at all, rather, it gives me more work because I'm alone doing the work. If they can give me a laptop, I can even go home with it to complete my work.

4. **How easy is OpenLMIS to use in your daily work?**

- I use the OpenLMIS weekly because I do my data entry weekly. Although, sometimes it may take more than a week, like, right now I have not done the one of last week, because I had a training somewhere. The OpenLMIS has not been easy to use in my

work, rather, made it more difficult for me. The OpenLMIS has integrated well in my workflow, but has been hindered by things like data, electricity supply, availability of good computer system and network. It has helped us to be accurate. Above all the problems it has for now, if they can make it to be easier, it will be good.

5. **Are there any features of OpenLMIS you find particularly helpful or unhelpful?**

- Like I said before, in the issuance, if there is a way the OpenLMIS can be redesigned that one can just enter the items once, instead of entry them one after the other, it will be better. Infact, the main problem is just that I do not have enough hands. For now, I have not seen any feature not helpful. The feature that I find interesting was when I entered a wrong amount of vaccine I counted and the OpenLMIS indicated it and I had to recount again and discovered the problem.

6. **What factors have enabled you to effectively use OpenLMIS in your work?**

- Some of the factors that have helped me in the use of OpenLMIS is just the training I have received from the state level and UNICEF. The training was once from the state level and once from the UNICEF. I have never received any support apart from the initial moment we were given internet data and that was when they gave us the Tablet. There are not enough resources allocated to the OpenLmis. The OpenLMIS is valued, only that they need to improve in the connectivity and we need more hands. Well, I do not know if there are any organisation that provides funding to the use of OpenLMIS.

7. **What challenges have you experienced with using OpenLMIS?**

- Apart from the challenges I mentioned earlier, the OpenLMIS cannot be used with Chrome browser and even on Firefox it takes time to open, but if you do it on the computer system it will open easily. There are facilities that are not included in the OpenLMIS. The training is not enough and we need more skills.

C. Recommendations and improvement

8. **What improvements would you suggest to make OpenLMIS more effective for future mass vaccination campaigns as well as routine immunization?**

- They should improve the OpenLMIS so that its speed can increase. They should help us in the area of internet data, give us computer system.

Participant No. 013
Location: FCT
Respondent's Gender: Male

A. **The stock management system and flow of data from paper-based to OpenLMIS**

1. **Describe your role and the stock management system you currently use for vaccine stocks**

   - My role is to receive the stock distributed to this health facility, issue them to the appropriate personnel(s)/health facility as may be needed for vaccination and immunization campaign, I also take data management of all the vaccines and equipment distributed to this health facility.

2. **Describe the flow of data from health facilities to higher levels of the supply management system.**

   - As I said before, I am the one who takes charge/care of the issuance of the vaccines from this LGA council level to the communities' health facilities in this Kuje LGA. We have about 49 community health facilities in this Kuje area council. We also receive data report from the community health facilities, then prepare ours and send to the state level. Let me allow my colleague to come in here, because he is the one who enters this date in the OpenLMIS and forward to the state. The colleague (Second person), I enter the data collected from the community health facilities into the OPENLIMS, and ask the routine immunization officer to confirm and then send to the state level.

   - The colleague (Second person), yes, we have been having discrepancies between recording and the actual stock level. Sometimes when we check to confirm the actual levels, we will find out that from the community health facility level, when they want to record the quantity used, they make mistake (under/over quantity) and when they come

to us, we put them through. For now, they have been getting it right and they have also improved.

B. **OpenLMIS user experience, benefits, enablers and barriers**

3. **What are the main benefits of using OpenLMIS, compared to the paper-based system?**

- The main benefit is that it is better than the paper-base, when we just collect the data, we qui-in the data into the OenLMIS and then generate our report and send, we can quickly see our stock balances, unlike the paper-base, where you have to check and trace these balances (It consume time), the OPENLMIS is easier for me. The colleague (Second person), when we qui-in the data and there are discrepancies, it quickly shows immediately, we see it, and then correct the mistake before sending out. It has helped improve my work by saving me time, I can easily see my stock balances anywhere I am, and I can quickly and easily send my report from anywhere I am.

4. **How easy is OpenLMIS to use in your daily work?**

- We use the OPENLMIS weekly and not daily. It is easy for me now because initially I do not know how to use it, and I had to call someone from the state who taught me how to use it. Since then, it has been so easy for me to use. Even though the system is difficult to use, but, after the person I called to put me through came, it has been easy for me. The colleague (Second person), it is easy and fast to detect error(s) from the system than the paper-base. The hard or difficult aspect is the internet, most times network always disturbs the system so much, there are times it takes up to 24hrs for me to qui-in and upload the data into the system. Sometimes I don't have internet data, it becomes difficult to upload the data into the OpenLMIS. The OpenLMIS has integrated well into my workflow. Moreover, it does not show me expired vaccine or soon to expire vaccine.

5. **Are there any features of OpenLMIS you find particularly helpful or unhelpful?**

- Yes, there are features that has been so helpful to me. Colour green helps me know when my work is well done, and colour red helps me know when there are problems.

There are colours I think are unhelpful to me. Colour like black are not helpful to me and I don’t see the need for that colour. The balance/stock level and reorder feature help me place an order quickly in order not be stockout easily. There are many other features in the system that are not helpful to me, but I think it may be helpful to other officer, and I will not say it should be remove from the system. The system helps us keep adequate data and manage of vaccines.

6. What factors have enabled you to effectively use OpenLMIS in your work?

- When I came into this office, one of the officials from the national level came to touch me how to use the system. I have had nonofficial training ones, and one official training from Mrs. Amina (Online). I was giving this Tablet from the state and it was handed over to me by my predecessor who I just took over from. No enough resources are allocated to the use of this system. We need help in term of internet data. I value the OpenLMIS so mush. I value it because it helps me do my work effectively, easily and promptly. No any organization provides support to us for the use of this system. I value it because our superior officer can easily access information from the system without contacting us, unlike the paper-base, when they see we are not doing better, they can call our attention. The second colleague, I also value it because I don’t need to look for transport-fare to the town myself to carry vaccine, through the OpenLMIS, the state can see what I have in stock by various vaccine and before I know it, my vaccine stock is sent to me.

7. What challenges have you experienced with using OpenLMIS?

- For now, as an individual, I don’t have any challenges. The internet network is one of the challenges, finance is another challenge, other challenges like staffing is there, and the issue of availability of electricity light, and computer system. We do not have enough training and we do not have the enough skill to use the OpenLMIS. The support we need are more training, more staffing, we need more internet network and internet data.

C. Recommendations and improvement

8. What improvements would you suggest to make OpenLMIS more effective for future mass vaccination campaigns as well as routine immunization?

- It is important they include a feature that will indicate vaccines that are not useful for a specific purpose. It is important they improve in the colouring. Yes, there have been improvement in the system since it was launched, like before, it takes time to see the antigen you are looking for, but today, when you just click it will take you to the antigen you want to see.

Participant No. 014
Location: FCT
Respondent's Gender: – **Male**

A. The stock management system and flow of data from paper-based to OpenLMIS

1. Describe your role and the stock management system you currently use for vaccine stocks

- I am currently the FCT Vaccine Security & Logistics Consultant and was recently given a new position two weeks ago which is the Immunization Supply Chain Consultant for the National Strategic Cold Store. I visit the National Strategic Cold Store at least 3 times a week and the State Cold Store at least 2 times a week to supervise the flow of vaccines from the National Store down to the States, from the States to the LGAs and from the LGAs to the facilities.

2. Describe the flow of data from health facilities to higher levels of the supply management system.

- Vaccines are usually sent from the National Strategic Cold Store to the Zonal , to the State, to the LGAs which are then distributed to the facilities. All the 5 levels of the Immunization Supply Chain are suppose to use OpenLMIS except the facilities who usually send their stock balances through SMS. While the LGAs, State, Zone & National Strategic Cold Stores, transactions are posted on the OpenLMIS.
  -We do observe discrepancies in virtually all the Immunization Supply Chain levels which is a major problem. And because of this, our partner CHAI has been monitoring the OpenLMIS now in the last couple of months. CHAI tried to monitor and even a kind of score all Immunization Supply Chain levels most especially the National, Zonal & State on their performances towards things that has to do with the discrepancies between vaccines, diluents, expiry and so on. To ensure that people that are handling the tool are giving more concern on these areas because they are key indicators that are supposed to be monitored to ensure that things are going well according to plan.

B. OpenLMIS user experience, benefits, enablers and barriers

3. What are the main benefits of using OpenLMIS, compared to the paper-based system?

- Documenting it is web-based where everybody can assess it and harvest it for any purpose and this is better than the paper based, because in the paper based, you either make a call or go to the store yourself to see and make comparism before you get what you want to use the data for.
- OpenLMIS is very nice to use but their previous tool is more friendly than the OpenLMIS but had to be discarded because it was to expensive to maintain.

4. How easy is OpenLMIS to use in your daily work?

- It is very easy to use for example, if I want to find out LGAs that have recorded their data in a week, I will just go there and check the Immunization tracking to find out those that have uploaded their records. It also has more accuracy and visibility. But sometimes the inability of the LGAs to upload their data despite all the calls and reminders because of the lack of data has really caused a friction in the use of the tool.
- It is really additional work especially for those that have a large number of clients; it is time consuming to enter the total number of products or items released to each of the Immunization Supply Chain levels that come to pick up their vaccines.

5. Are there any features of OpenLMIS you find particularly helpful or unhelpful?

- The features in the OpenLMIS are all more than helpful, except the process, because it is web-based and every level you opens it delays because of the number of the Child Stores that are uploaded on it. However, because of this it takes time to give you what you are looking for.
- In taking and making decisions the OpenLMIS helps, but we have challenges from the higher level, for example, stock out is not something any one even those outside the Immunization Supply Chain Levels will want to have because it will lead to missed opportunities, your clients and customers will miss one thing or another. But because of the storage capacity and funding from the side of the Government, we naturally have stock out. For example: if we are suppose to get 1 million naira for the procurement of vaccines and the Government only releases 70% of the money, stock outs are unavoidable. The tool however, does not give any form of reminders or alerts, so one has to be checking it weekly to ensure the necessary information are uploaded to be able to avoid stock out from the Child Stores.

6. What factors have enabled you to effectively use OpenLMIS in your work?

- We are grateful to those who took their time to develop the tool for us to use, that is GAVI. Our sponsors CHAI also took their time to invite us when developing the tool to give our input on how the tool will look like which made us to have more interest in the use of the tool. The OpenLMIS is also helping the system solve a lot of problems.

- When CHAI was developing the tool, they invited us for training in which we gave our input and afterwards, we were also invited for a 5 days trainings in which we were taught on how to use the tool. After that, we had a step down training with the States. There is basically hardly any month that will go by without a training or two being done to train health workers on the use of the tool.

7. What challenges have you experienced with using OpenLMIS?

- Individual: It is cumbersome and considering the economic hardship we are going through right now, you expect people out of their merger salary to spend 1,000 naira a week to be send their stock balance, it is not visible. It is also less friendly compared to the former tool used.

- Technological/Environmental: The common one is network. Sometimes we will want to open the tool but because of the nature of the network we have in this country, you will find out that it will not open especially if it rains heavily. Also the number of people using it is more than the space provided for the tool so it leads to network congestion.

- I feel that I don't have the necessary skills and training to use the OpenLMIS because anything that has to do with ICT is a continuous learning process. I can not say that I am 100% perfect in the use of the OpenLMIS, that is why I learn from my pairs and supervisors in an opportunity I get. The additional support I need is still all the training that I can get to better use the tool.

C. Recommendations and improvement

8. What improvements would you suggest to make OpenLMIS more effective for future mass vaccination campaigns as well as routine immunization?

- There is supposed to be provision for put- away in the tool, there are no packing list with destinations or locations of where the vaccines are located, so if such can be added, it will be helpful.
  Data should be provided especially to the LGAs to enable the easier flow of data from the LGAs to the States.

- In the previous OpenLMIS we were using, the distribution of the vaccines terminated at the State level but now you can go down to the LGAs and find out the products that are available in the LGAs.

Participant No. 015
Location: FCT
Respondent's Gender: **Male**

A. The stock management system and flow of data from paper-based to OpenLMIS

1. Describe your role and the stock management system you currently use for vaccine stocks

- I am the LGA Cold Chain Officer who is in charge of overseeing the vaccine collection from the State to the LGA. I am responsible for ensuring that the vaccines are rightfully distributed to the facilities that come to collect them. I am also solely responsible for entering the vaccines data and records on the Open LMIS.
  The Stock Management System is done on a weekly basis. At the end of the week which

is every Friday, we do a physical count and then compare with the records in our registers the vaccines issued to the facilities and then enter the figures in the OpenLMIS.

2. Describe the flow of data from health facilities to higher levels of the supply management system.

    - Vaccines are received from the State level, after receiving the vaccines, we do a background check to cross check whether the vaccines provided are the same with the figures in the receipt. The vaccines are then stored. The various facilities then come for the collection. We give them according to their target population and most of the facilities we have here in our area council collect vaccines on a monthly basis. At the end of the month as they are coming back for the collection of another vaccines, they come with the report of the children immunized for the previous months based on the vaccines received.
      The discrepancies are just few, for example, when some facilities return vaccines, there are usually more of the physical than the ones recorded and this has not been a major problem because they always easily reconcile it.

B. OpenLMIS user experience, benefits, enablers and barriers

3. What are the main benefits of using OpenLMIS, compared to the paper-based system?

    - It is very easy to enter the data and send on the OpenLMIS compared to the paper based.. It also helps me to know my previous stock balance in comparism with the present stock balance at hand online.

4. How easy is OpenLMIS to use in your daily work?

    - I use the OpenLMIS on a weekly basis not on a daily basis. It is a form of phone, it is tablet which makes it more potable and easier to carry around and fill the necessary data. It blends well with my overall work flow but sometimes, when there is no light and the tablet is down. It causes delay in entering the data which causes friction in my work flow.

5. Are there any features of OpenLMIS you find particularly helpful or unhelpful?

    - I find all the features helpful except the features that captures the recording of the Cold Chain equipment which I am finding it difficult to do because I need more training, it is an important feature that I need to use but I am finding it hard to use it which is why I hardly use it.
      The OpenLMIS does not give any reminders or alerts. I only get reminders from the State Cold Chain Officer through calls or SMS which affects decision making.

6. What factors have enabled you to effectively use OpenLMIS in your work?

    - Individual: I find it much more easier to enter the data on the OpenLMIS.
      Organizational: It is part of my commitment and job description so I have to do it.
      A UNICEF consultant came for the training twice this year to train us on the use of the OpenLMIS but I still need more training.

There are not enough resources channeled towards the OpenLMIS and I think the tool is valued by those in charge because if it was not valued by them the State Cold Chain Officer will not be calling me every week to update the records.
No organization has provided funding in any way to the system implementation and maintenance.

7. What challenges have you experienced with using OpenLMIS?

- Individually: I am not motivated to use the OpenLMIS because I am the one funding from my pocket the cost of data I use to upload vaccines records. I have had some trainings on the use of the tool but still need more trainings to help me in some other areas of the tool.
  Organizational: I am the only one using the tool and need more hands which makes the work much.
  Technological: If there is no light, I have no way of charging the tablet that I am suppose to use for the OpenLMIS.

C. Recommendations and improvement

8. What improvements would you suggest to make OpenLMIS more effective for future mass vaccination campaigns as well as routine immunization?

- If the OpenLMIS can be extended to the facility level because currently, it is just in the LGAs and has not reached the various facilities yet so as to be able to monitor vaccine input and output.
  Before we had network issues in regards to the OpenLMIS, when we upload data and submit , it hardly goes but now it does not have such issues, when submitted it goes immediately. And before after submitting the second time, the data will clear off which will make one start all over again.

Participant No. 016
Location: FCT
Respondent's Gender: Male

A. The stock management system and flow of data from paper-based to OpenLMIS

1. Describe your role and the stock management system you currently use for vaccine stocks

- Presently we use both electronic and paper-based, we use to do arrivals, stock distributions for stock management. On the paper based, we use the ledger and we also use bin cards.
- In the stock management (by design I am supposed to be an admin manager but the product manager is not disposed at the moment) I am responsible for the arrival and distribution for now.

2. Describe the flow of data from health facilities to higher levels of the supply management system.

- At the health facility level, we don't have any stock management for now, what we have is DHIS which is service delivery tool. From DHIS, we get their consumption, because I have national access to DHIS, so if I want to do anything pertaining to consumption, I go to DHIS; from there we go to the Lga, then the state and the National. (i.e., using DHIS)
- But for OpenLMIS we get data on stock management; we go from the Lga, to the state, and then the national. We don't have OpenLMIS in health facilities
- The service data proceeds from health facilities, but stock management (their supply chain data) starts from the Lga upwards.
- There are discrepancies; supply chain data is very broad now, for example in OpenLMIS, for us here, there might be discrepancies, because of the little challenges we are having, the challenges of space, shortage of human resources, so we really have, for example, now we are packing vaccines, if you go to our cold room, its somewhere you cannot even move freely through (space for movement in our cold room is very small,)we have not be able to really provide enough space, so that if there is any discrepancies, you can easily go and check, the whole place is chocked up, but we are working on it for now. Though not in every area, there are still some antigens we are easily correct discrepancies for.
- The discrepancies do not really create any problem; we can easily solve the problems if they come up.

B. OpenLMIS user experience, benefits, enablers and barriers

3. What are the main benefits of using OpenLMIS, compared to the paper-based system?

- OpenLMIS is more accurate, when it comes to summing up, (arithmetic aspect of it), you can always get the correct answer, unlike paper based, you can even use the calculator, but sometimes, it gives wrong answers. So the accuracy of what you put in is always is always there, but its garbage in, garbage out, if you put the correct figure, you get the correct figure, if you put the wrong figure, it will give you what you put in. So it is far batter; if you want to solve a series of numbers, it can always give you the correct answer.
- And again it's a live site, you can always see what you have at any point in time.
- It has really improved us because before if you want to get stock balance, you have to call the lga, but now, from here I can get all the details and I can just call to confirm if the data for any lga (as contained in the OpenLMIS) is correct or not.

4. How easy is OpenLMIS to use in your daily work?

- Very easy, very easy, the only problem is if you don't have internet; I have told them at the backend that they need to work on the internet. Our internet is bad, it can frustrate you, somebody called me yesterday that he wanted to do some, I said, relax, it might be your internet.
- It needs to have enough data for you to access the platform. But overall, it is very easy to use.
- No, it does not make the work harder except when there is internet problem. If you have internet, I can capture these data in a few minutes.
- It has blended well, because when it comes to the reporting aspect, you can use it to make decisions, as a data manager, I really rely on it to do my work. The dashboard, the charts, it makes the work easy, I can pick the data immediately, screenshot it, use it for

presentation and the rest.
- It creates frictions at times, when somebody is not doing the right thing form his/her own end, we may be getting different outputs.

5. Are there any features of OpenLMIS you find particularly helpful or unhelpful?

- All of the features are helpful. There is none of the features that is not helpful for now. It's about the use, the other aspect of OpenLMIS that we have not used is the bottom-top approach, but it is very very effective, however it is the use that makes it helpful.
- Yes, there are some of the features that I rarely use; like we have not started using the requisition module (that is the bottom approach). Also, we are not really using the equipment management feature; even though we have entered all our equipment into it, it has not really given us an idea of …. For example, when I was using the SMT, you enter all your equipment (your equipment is connected to the stocks thus at a glance you will see the on that equipment its space, net volume, etc.)
- The full use of the equipment and the temperature feature should be included in the OpenLMIS (though I heard they are working on the temperature feature.)
- There should be a scanner for entering stocks as they arrive, such that by scanning, the details of the product are captured into the application in real-time. (barcode scanning, and it is best that the barcode is on the carton, not on each vaccine so as to make the data capture process faster)
- We are given a small phone for scanning, but we are not using it because of the quantity of vaccines that we have., we have human resources issues.
- But they need to improve on the alert systems of the app, the APP should keep track of the vaccine stages, expiry dates. (Because presently we have many stores with vaccines that are near expiration, but they don't even know.)
- Yes, there are decision support systems within the OpenLMIS; they have been helpful to prevent stock out, alerts like the physical inventory alerts comes in like Fridays (from the app); nudging me to go and do physical count. The alert goes down to the Lga. On our own, we don't really do that because we are always using the system on daily basis.

6. What factors have enabled you to effectively use OpenLMIS in your work?

- My personal interest, dedication and on the job training has enabled me to use the system. CHAI is always available to assist.
- But the major thing is interest, you can't use any system without your interest in it
- Organizationally; provision of internet has helped us, CHAI always provide for us at the national level for now.
- Environmental: Network has been favorable to us most times because NPHCDA has contract with Galaxy back bone (GBB), so, like now, that is what I am using, immediately you come to the facility, you connect to it; though there are downtimes, so at such we resort to our person device network
- Technologically; GBB provides the internet connection to the facility.
- Yes, for Nigeria, we have had internal trainings at the national level, zonal, state, even Lga level.
- We also have zoom trainings (by CHAI) that we usually conduct for all levels.
- We have a WhatsApp platform where all the states and Lgas are connected. These platforms are there to help anybody that has issues
- We really need training from outside, there was a time Vital Alliance wanted to have a training for us, but it didn't hold.
- The training was only one (i.e, that on we had at the national level, state level and the

Lga level) last year) We have not had any training this year. The ones we have had this year are zoom. But the normal national training was last year.

- The training form the national is stepped down to the state and then Lga.
- The training is implemented by CHAI in collaboration with NPHCDA

7. What challenges have you experienced with using OpenLMIS?

- Individual challenge is when I am having network down time,
- also, there are challenges with viewing the history of a particular batch, when the application at times will throw an error
- the login stays for a long time
- sometimes it does not work on several browsers,
- to download reports takes time
- financial resources; people at the lga have network issues,
- getting data is also a challenge; CHAI gives us data at the national level, but it does not get to the Lga, zonal; though it's not their fault, because there funding is also limited. Lgas, state and zonal are using their personal money to get data, so they are discouraged, coupled with downtime issue.
- also the tablet is another challenge, they get spoiled easily. The quality of the equipment should be improved.
- Necessary skills: I would say 50%, because I still need mores skill, the administrative skill and the technical skills. My downlines really need training every time. Because, when someone is replaced, the next person should be able to take full responsibility.
- Everybody should have this training
- it takes a long time to add a user.
- Download content of stock status is different from what is on the system stock management. There is no proper syncing. The data that is viewed is different from the content of what is downloaded from the same report
- when you want to view the history, sometime you get an error message and you won't be able to view the history, and the history is very important.

C. Recommendations and improvement

8. What improvements would you suggest to make OpenLMIS more effective for future mass vaccination campaigns as well as routine immunization?

-
- They should improve the speed of the application
- Make it user friendly
- Improve on the equipment module
- Include the temperature module
- Improve the system notification
- Improve the aspect of adding users; due to changing man-power in the system.
- Improve the administrative features.
- Any improvements: It has improved, before, the coloring was different from what we have, it was blue, now green. They have also improved on the physical inventory. The reporting aspect has also improved.

Participant No. 017
Location: Lagos
Respondent's Gender: Female

A. OpenLMIS and its role within the EPI Programme and beyond

1. What was the rationale for the rollout process of OpenLMIS for COVID-19 vaccination and subsequently Routine Immunization (RI) implementation?
   - *To the best of my knowledge, openLMIS was designed for data visibility on vaccine transaction and distribution across the states. In the decision office, there are many forms, and we all have our own prescriptions, but when it comes to the inputting data into the openLMIS, I don't do it. They do it, but I supervise, and I ensure that any vaccine transaction that is done, whether we push it out or the CCOs from the local government come to us, I make sure that it is inputted into the openLMIS and I also go through it to see that the data is correct.*

2. Describe the role OpenLMIS currently plays within the EPI program.
   - *OpenLMIS plays the role of data storage for managers to work with by the use of vaccine data availability to plan effectively and efficiently and allocate needed vaccine to the program.*

3. How has OpenLMIS impacted your decision-making and program management?
   - *OpenLMIS has impacted my decision making capacity positively, in the sense that if any Local Government CCO do not perform well, I can easily see and call them to order. If there are data they have not been inputted, I can call such CCO to make correction and input it. It helps to know where and who needs training and it helps to know where to direct resources.*

4. To what extent is the OpenLMIS interoperable with other information systems (DHIS2)?
   - *I will say openLMIS and DHIS2 are different soft tools. Eighty percent (80%) of what we get on DHIS2 are also on the OpenLMIS. Although, you can't do some task on the DHIS2 but on the openLMIS because openLMIS is majorly for vaccine transaction across the state*.

5. Is the OpenLMIS already being used in other health areas (i.e. for other health commodities)
   - *OpenLMIS like I said earlier, has nothing to do with other health commodity expect vaccine distribution and transaction. It helps to know the areas that need vaccine more and the area that has more than enough.*

B. Barriers and facilitators to adoption, data quality, and use of OpenLMIS

6. What are the main barriers to the adoption, use, and data quality within OpenLMIS?
   - *I don't see any issues, maybe because I have more experience. I was the state immunization officer for six years, and I know that when WHO or any international bodies want to do assessments in Lagos, they ask for four to six years paper-based data which is most time unavailable as a result of misplacement*. *I have mentioned some of the benefits of using openLMIS, I stay in my office here and I look at the number of doses of each of the vaccines that these LGAs have. I get to know the last time that a cold chain*

officer utilized the OpenLMIS, and then I also know that a transaction just took place now or yesterday or the day before. So those are the benefits. Because before, if it is paper-based, I will have to call them.

- Also, data subscription. Many of them are ready to work but they don't have money to buy data, it can be sent directly to their registered lines and once that is done nobody will have any excuse why not to deliver. Considering the economic situation of this country, they need motivation. Lately, I think UNICEF is giving something.

Probe: Any user groups within the program or supply chain who have difficulty adopting or using openLMIS effectively and why?

- *I think we have more women in the field than men, because many of the women are nurses or from other medical background and you know that nursing profession is still female domiated but we can't not say that women are not doing well in that regard as we know women are very inquisitive, they want to know more on whatever they are doing, though we do see the lazy ones.*

7. What factors have facilitated the successful implementation and use of OpenLMIS?
   - *OpenLMIS is now useful because it helps to create awareness. It's will tell you that the zonal store has issued certain amount of doses of vaccine to the states. Even when you are not around and the officers are yet to get to you in terms of reports, you have that first-hand information that the vaccine transaction has taken place.*

Probe: What strategies have been successful in overcoming barriers to openLMIS adoption and improving data quality?

- *We ensure that the cold chain officers get adequate training that will help them perform their tasks on the openLMIS. In the area of monitoring and evaluation, we ensure we follow every of their activities on the openLMIS.* If we notice that it is more than 24 hours of the openLMIS utilization, then we call them to know how they are doing and ask if they have any issue around the use of openLMIS. This has help to create caution for those who may want to be lazy with their job.
-

Probe: Which organizations are providing funding to system implementation and maintenance?

- *UNICEF and WHO*

Probe: How often are training activities around LMIS conducted and who implement the training?

- *The cold chain officers meet regularly either at the cluster or at the LGA levels because they have mentors assign to each officers. This was done to ensure officers have access to human resource whenever the need arises because errors while working on the openLMIS need to be avoided so as to have a reliable data, and I can say that it has been so impactful.*

Probe: Do you think there are enough resources directed towards openLMIS?

- *Yes, UNICEF and WHO are trying their best but Lagos state government needs to support especially in the areas of data subscription provision.*

C. Perceptions on the link between OpenLMIS data use and immunization coverage

8. Have you noted any links between OpenLMIS data use and immunization coverage,

completion, and timeliness?

- *There is a strong link to some extent because openLMIS is use for vaccine transaction, and the data generated helps to know the number of vaccines in stock for a timely distribution for immunization program across the state.*

Probe: Describe specific correlations between data use and improved program outcomes (e.g., higher coverage rates in higher LMIS usage locations).

- *To the best of my knowledge, openLMIS has helped in knowing the adequate number of vaccines for a particular location based on the number of people that come for vaccination in such area. For instance, the data submitted by the LGAs to the state will help the state to prepare for the targeted population for the future immunization program.*

9. Has the use of the OpenLMIS led to any unexpected effects on vaccine delivery or staff roles?
   - *Yes, this is because the paper based system has not been totally eradicated. After registering their data on their ledger, they still input it in the openLMIS which to me increases their tasks. There is only one cold chain officer in every LGAs who may not have an assistant, how do you expect such person to meet up with deadlines, so it has really put more load on them. However, the cold chain officers are trying their best.*

D. Learnings and recommendations from OpenLMIS utilization for COVID-19 vaccination

10. What unique lessons were learned (positive and negative) from using OpenLMIS for COVID-19 vaccination, and what recommendations do you have based on these lessons that can be applied to future large-scale campaigns and outbreak response in Nigeria or other countries?

    - *For the equipment management on OpenLMIS, they ask the cold chain officers to input all the equipment: passive, co-training, working group room, freezer, and then somebody woke up again and brought IGAR, Event Trigger Analysis. All the equipment that they have put in one tool will still be put in the other tools which is not a challenge for me but it is a big challenge for the people that I'm working with. The same thing OpenLMIS is asking on equipment management is what IGAR which another tool requesting too.* If IGAR is replacing IRP, then they should quickly harmonize these tools rather than making the people using them to have three or four different tools.

    - *Another recommendation is the provision of personal computer because majority of go to business centers in order to get their work done, this will delay report. Let the state and NPHCDA take full responsibility because they are not supporting this people as much as I know.*

    - *Again, data subscription should be provided. Let them know that a certain amount of money will be given to cover for their subscription as this will encourage the officers.*

Participant No. 018
Location: Lagos
Respondent's Gender: Female

A. **The stock management system and flow of data from paper-based to OpenLMIS**

1. Describe the flow of data from health facilities to higher levels of the supply management system.
   - *From the first facility which is the health facilities, the data that is generated are basically service delivery data. When they immunize, tally sheet must first be filled which is their primary source of immunization data. They also have their register and vaccines management tools (VM tools). LGAs also use DHIS tools but they primarily depend on VM tools. The LGAs report their immunization data including the number immunized and vaccines doses used. The health facilities submit their data to the LGAs, the LGAs use VM and DHIS tools to report data to the state, while the sate collate the data and send to the national using the openLMIS.*

Probe: How often were there any discrepancy between the recorded and actual stock level?
- *Yes, there are oftentimes discrepancies that occur but let's leave it at that.*

Probe: That means it happens often, like frequently?
- *Depending on where it happens, some are so conscientious, they ensure errors are avoided, but generally, I would say like 50-60% of the time, you will have discrepancies.*

Probe: Can you share a specific example, just one example?
- *There is one thing about vaccines depending on the quantity you have, at the lower level, it may be easier to count physically. As you go higher there are more doses in stock and it becomes more difficult to count. As a result of this, if I count now and an audit comes in to recount, there is every possibility that we get different figures. So if I have input my figure on the openLMIS which is different from figure the audit got that means discrepancy has occur.*

Probe: Can you describe this as a major problem?
- *Well, depending on how meticulous we are and also how much of human resource we have.*

B. **OpenLMIS user experience, benefits, enablers and barriers**

2. What are the main benefits of using OpenLMIS, compared to the paper-based system?
   - It's fast to access, anyone can see what am inputting as long as you can access the openLMIS. It helps with accountability far more than paper-based system which has more errors.

Probe: Can you give an example of how OpenLMIS has really improved your work?
- *Before the OpenLMIS was launched, we were using some other electronic stock management tools, but openLMIS it's real-time because everybody sees what am doing*

as long as they can access it. It's really helpful to me because those that are above me can see whatever am doing and I can see those under me do too.

3. How easy is OpenLMIS to use in your daily work?
    - *This is a zonal store and it peculiarity is that it also serves as the national hub for devices like syringes. I don't have issues entering vaccines on the openLMIS but my major problem is with the devices because we are getting more than 20 containers of devices weekly. Let's say Mina for instant, this is the shipment document which is about 4 pages with their batch number written on them, sitting down to enter all of this in a day is not easy knowing well that I have other things to do in the office.*

Probe: How you now saying the openLMIS is doesn't make your work easier?
- *For the vaccines, no issue at all even with the other electronic stock management tools we use.*

Probe: How well can you say the openLMIS integrate with your existing workflow?
- *It has integrated well for me, although there are challenges like I said. The person that used to work on the openLMIS is no longer around, so we are short-staff, I have to combine the work with other tasks which has been challenging for me.*

4. Are there any features of OpenLMIS you find particularly helpful or unhelpful?
    - *What I like most is the adjustment*

Probe: Is there anyone you feel it's not helpful?
- *The physical inventory, I have used it many times but I stopped because it was giving me headache. Maybe it can be made simpler in the sense that we would be able to count what is there physically. For instance If I want to do the physical inventory of my BCG vaccine, I should be able to take BCG vaccines and do the physical inventory. Rather than doing the physical inventory of my entire stock.*
- *Another one is the requisition module, we rarely use.*

Probe: Is there any functionality that you want them to add in order to make your work easier?
- *The arrangement on the openLMIS is not good. For instance, if I have BCG which has many products and different manufacturers like VCG serum and there are different manufacturers then you have to go again or maybe even turn pages before you see another BCG like Maybe BCG Japan or BCG Interfax. It doesn't make sense. There should be a place to click that will give me the total of all my BCG vaccines but it's not on the openLMIS.*

Probe: Looking at the decision-support algorithms like alerts and reminders on the openLMIS, have they helped you in preventing stock out?
- *It doesn't give me any alerts until I open it, but when am working on the tool it will tell me what and what am not doing right. There should be an alert that a vaccine is expiring in three weeks' time so that the careless type who does not open it can know what to do and also to know one doesn't go out of stock, maybe by sending mail.*

Probe: Can give an example where it has helped you to make a decision?

- *When I'm issuing on low stock for example, and the stock is depleting it will send email telling me that I have new stock but it will only send such message because that particular batch is low so in actual sense the stock total of that antigen that I have may not be new. Like I have VCG Serum Institute now, I have several batches there if I issue and exhaust batch it will send me a low stock alert but that does not mean that the other batches they are not more than that.*

6. What factors have enabled you to effectively use OpenLMIS in your work?
   - ***Individual factor:*** *Determination, one has to be very determined in order to get work done on the openLMIS.*

   - ***Organizational factor:*** *Because both national and CHAI are on it and do not give any break on the use of openLMIS and they are really supporting us from time to time in terms of training and score-card.*

   - ***Environmental factor:*** *I can say the tools are available for use*

   - ***Technological factor****: The availability of tools to us, just that they do not make provision for data unlike the LGA officers who get data.*

Probe: What training and support have you received that enabled you to work effectively?
- *We were trained by CHAI, but that doesn't mean it's only CHAI but they are the one facilitating it.*

Probe: What type of support have you received and who are the sponsors?
- *I don't know the level of support they are bringing to the table but in terms of technical support I think we are getting that, UNICEF, CHAI and others are giving their support in terms of training and not incentives.*

Probe: How often do they organize such training?
- *I don't attend every time but I think it's weekly*

Probe: Do you think there are enough resources directed towards openLMIS?
- *They directed a lot on data though am not receiving any but those at the LGAs do get data from them. Presently the only problem is that we can see the LGAs but we can't see their facilities on the openLMIS.*

Probe: Do you think they are valued by those in charge?
- *They are the one that can answer that, I can't speak for them.*

Probe: Which organizations are providing funding to system implementation and maintenance?
- *I'm not sure and I don't want to say what I don't know*

7. What challenges have you experienced with using OpenLMIS?
   - *In time past it used to be network. And again, the devices first come to me and I register the batches, if a make a mistake I can't correct it and that mistake is what it will be seen on the openLMIS. For instance, was making some entries last week Thursday, I made a mistake with the numbers and needed to delete but openLMIS says I can't delete it. I*

wanted to delete it because some people will just click this wrong batch as what they have received, but openLMIS says I can only update and I updated

Probe: Do you feel you have the necessary skills and training to use openLMIS effectively?

- *Yes, however, nobody is an island of knowledge, training is needed from time to time.*

Probe: What additional support do you need?

- *Like I just said, timely training is needed.*

C. Recommendations and improvement

8. What improvements would you suggest to make OpenLMIS more effective for future mass vaccination campaigns as well as routine immunization?
   - ***Future mass vaccination campaigns:*** *When you go out of Nigeria and see how things are done, the question you will ask yourself is, when are we going to get here? If we really determined to do it we can do it. I look forward to a time that as we give the jabs physically, electronically the update is done. This way we can't lose any device because it's automatic and it's already linked to the person. We did something close to this during covid-19.*

   - **Routine immunization:** For routine immunization is always the same thing
   - *The openLMIS desk officer in Abuja should try from time to time to be able to make corrections since we can't delete our mistakes by ourselves.*

Probe: Have there been any improvements into the openLMIS since it was launched?

- *Yes, there is a lot of improvement. When we started how many people were using it?*

Participant No. 019
Location: Lagos
Respondent's Gender: Female

A. The stock management system and flow of data from paper-based to OpenLMIS

1. Describe your role and the stock management system you currently use for vaccine stocks
   - *I'm the assistant cold chain officer southwest zone, I have a senior officer, which I assist in this cold chain work. Our role is to issue vaccine and receive vaccine, and to also keep our data. And, also, it's not only vaccine we deal with. We also deal with devices, like syringes, which we also input in the openLMIS. Majorly, we do vaccine security by making sure our vaccine is well stored. It doesn't change VVM, and we issue to other Facilities from here.*

2. Describe the flow of data from health facilities to higher levels of the supply management system.
   - *Since the introduction of the openLMIS, it has made it easy for us to see how our data flows from the primary level to the higher stores. OpenaLMIS makes it easy for upper stores to view our data, to see our vaccines are being distributed in the lower facilities.*

Probe: How often were there any discrepancies between recorded and actual store levels?

- *We have sometimes, we also use a bin card for recording our data and sometimes we might count some particular doses in our bin card and another person count again and*

see different figure. This usually happen because counting of vaccine is not an easy thing.

Probe: What do you think is responsible for such discrepancy and how often?

- *Maybe we miscalculated while taking our stock balance, and it* happens sometimes.

Probe: What can we do in solving this problem?

- *When counting we must give it our full concentration and also distribute ourselves so that when somebody is counting a particular antigen another person will be counting too.*

B. OpenLMIS user experience, benefits, enablers and barriers

3. What are the main benefits of using OpenLMIS, compared to the paper-based system?
   - *OpenLMIS is more better than the paper based just that there is no way you will not have the paper based that you can refer to. OpenLMIS has helped in vaccine accountability and make our work easier. It has a lot of benefits because you can go back to it, and it makes the weekly stock taking easy. It brings a lot of changing into data. The only problem I have with it is that when you are entering a data in batches and you enter it wrongly, you can't go back to edit it yourself, the error will be on the system and you will not get that exact doses that you said remains in that batch.*

Probe: In a situation where this occur, how do we correct it?

- *It has to be corrected from the top, at the national level.*

Probe: Why do you think the openLMIS is programmed in such way?

- *To checkmate us and ensure we are been careful while counting the vaccines or inputting data on the openLMIS*.

Probe: Can you give us examples of specific tasks openLMIS has helped you improved on?

- *It helps taking stock more easily*

4. How easy is OpenLMIS to use in your daily work?
   - *It helps taking stock more easily for me, you can just be called to give update on the available vaccines you have in stock and since you do it every day so you are able to give accurate information so make me think faster.*

Probe: What specific tasks openLMIS has made easier for you while performing them daily?

- *When updating the vaccine stocks, openLMIS has made it easier for me and helped in taking the right decisions.*

Probe: How well the openLMIS integrates with your existing workflow?

- *It integrates well, and it's a great experience for me because what we have in the registered vaccine ledger corresponds with the stock on openLMIS.*

Probe: Can you give an examples of how it integrates well with your workflow?

- *Like updating the stock on the openLMIS has to be done with the paper based I mean the data on the registered ledger which has help in getting accurate vaccines available.*

5. Are there any features of OpenLMIS you find particularly helpful or unhelpful?
   - *No, for me all the features are helpful.*

Probe: You mean there is no particular tasks that you perform on openLMIS that you think is not necessary?

- *Yes, there's no part that is not necessary and there is no part that is hard.*

Probe: Do you think the use of openLMIS has helped in your decision making while performing your tasks?

- *It has helped a lot because it makes you know what you have issued to the other stores, by just opening your openLMIS, you know what they have used, what they have at hand. You don't need to start calling Just tap on the on the LGAs store, PHC stores, state store and you get the information you need.*

6. What factors have enabled you to effectively use OpenLMIS in your work?
   - *Many times there is loss of data I mean giving wrong data, this openLMIS helps me to rectify some data errors while taking stocks.*

Probe: Specific factors

- ***Individually:*** *It helps me to rectify some data errors while taking stocks.*
- ***Organizationally:*** *As a zonal store, if we are stock out we can know through the openLMIS and during these stock outs one can easily connect to the national store unlike before that you will need to send mails. This openLMIS makes the vaccine flow easy.*
- *Environmentally and technologically everything is fine here.*

Probe: Have you undergone any training on the openLMIS and who organized it?

- *Yes, I have gone for about 2 trainings on openLMIS since the inception. It was sponsored by NPHCDA, some of our senior staff at NPHCDA facilitated the training with other private organizations that were contracted to.*

Probe: How often is the training?

- *I think the ones have attended is yearly, although they don't invite everybody at once but don't know how they arrange those that would be attending but I have attended twice, year 2021 but can't remember the second year.*

Probe: Do you think enough resources was directed towards openLMIS and can you think it's valued by those in charge?

- *I can't really say*

Probe: Do you think it's valued by those in charge?

- *Yes, they value and cherish it if not they won't put much money on training.*

Probe: Which organization is providing funding to system implementation and maintenance?

- I *know it is my office, anytime they have any training, my office will invite us but don't know how it's planned and implemented.*

7. What challenges have you experienced with using OpenLMIS?
   - *Sometimes it takes time to open, when you try to open the app, it will just be rolling.*

Probe: What could be individual, organizational/environmental or technological challenges regarding the use of openLMIS?

- ***Individual challenge:*** *Has to do with the time it takes for the openLMIS to open while trying to access the tools.* ***Organizationally****: There is no provision for data to work on the openLMIS.*
- ***Environmentally:*** *I don't think so because the challenge you might say maybe bad*

network, but we don't have that. We have good network.
- ***Technological challenge:*** *This may be associated to large file on the openLMIS.*

Probe: Do you feel you have the necessary skills and training to use openLMIS effectively?
- *Yes. I do, but I will prefer more in order to be perfect at it.*

C. Recommendations and improvement

8. What improvements would you suggest to make OpenLMIS more effective for future mass vaccination campaigns as well as routine immunization?
   - *Training of more personnel on the system because if they have more people trained it will make the work easier. My boss, myself and one other lady were trained, the lady has left, and I will be retiring soon, so more people need to be train for continuity on the job.*

Probe: Have there been any improvements into openLMIS since it was launched?
- *Yes, there is improvement because before getting data from the primary facility is not an easy task*

Participant No. 020
Location: Lagos
Respondent's Gender: Female

A. The stock management system and flow of data from paper-based to OpenLMIS

[redacted]
- *We have online tools, but the openLMIS remains the number one because it's an everyday trip because it's used at both state and LGA levels so as I'm doing my own assignment on it here, the LGA levels too are also being done.*

2. Describe the flow of data from health facilities to higher levels of the supply management system.
   - *It's actually a two way system, it's from the higher to the lower and also from the lower to the higher. From the higher to the lower, we send down vaccines and commodities, pass information and to the LGA store, to the health facilities and they receive and send their vaccines.*
   - *We are using the tool to also issue, monitor our transactions. We have CCOs down to the lower level, which is the LGA level, and our facility relates with the LGA. There are times that they relate directly with the state, so they are reporting in line with the LGA, and the LGA will collect all the health facilities and relate to the states, same way at the state level, we also relate with the national.*

Probe: How often were there any discrepancies between recorded and actual stock levels?
- *I wouldn't use the word discrepancy but it is normal that there will be difference and the difference will be found when you are very sound in terms of stock management.*
- *It does occur, but if you do your physical inventory well it may not occur. There are times due to so many challenges including expiry, damaging of products, human errors this happen.*

Probe: In that situation what can you do?

- *You must first of all understand what happened. Sometimes if you are able to track it on time, you will be able to realize what happened, maybe there is a facility that you have not issued and if it takes a little bit longer, you may not remember, and for those that has to do with damaging of products and the rest, you may not be able to do more because sometimes you receive vaccines or other commodity that are been short dated for so many reasons. It's not anybody's fault, we know that there's a tendency that a viral may break, so these are the data actually do not tally.*

Probe: How frequent does it occur?

- *It's something that occurs frequently, because it's an everyday tool.*

Probe: Can you say these are major issues people have while using the openLMIS?

- *For me, it's not an issue because it's an everyday tool, so we use it every day and the more you use it the better you become that is why I said it's not an issue.*

B. OpenLMIS user experience, benefits, enablers and barriers

3. What are the main benefits of using OpenLMIS, compared to the paper-based system?
   - *One of the major benefits is receiving it at all levels, as I am now, have been on the tool since Morning, the same way at the national or any level, anybody can check.*
   - *Again, I can work on this tool anywhere. As a matter of fact, most times I work on it in the midnight doing transactions, doing the vaccine collection because it allows me to multitask. If I'm unable to finish that transaction in the office, it's not a big deal.*
   - *The ease of work for myself because I have been familiar with the tool. I can see my transaction since we have been imputing about 2 years now. I can go to the bin card and look at what I have done. I know I can see what is also happening at the LGA level.*
   - *There are some challenges at the lower level, but it's easy for me because I have my data online, the openLMIS is like an online ledger.*

Probe: Can you give me some specific examples how openLMIS has helped improved your work?

- *I will talk about visibility, am able to work anytime and anywhere. It's also universal.*
- *As I'm using it, all of my colleagues are using it. I can also see what is happening at both the zonal and national levels.*
- *Again, OpenLMIS is a good data collection system because I can track what I have done two (2) years ago.*

4. How easy is OpenLMIS to use in your daily work?
   - *It's been very helpful in performing my daily tasks especially because I can work remotely.*

Probe: Is there any specific task on your daily work that openLMIS makes easier or harder?

- *It's both, the openLMIS is an added work to what we do because it does not take out the other tools which is our ledger, so it is another work that is beneficial to us. The benefit i's still there even though it has given us more work but I don't see it that way because of the visibility.*

Probe: How well openLMIS integrates with your existing workflow?

- *Yes, our workflow harmonized and also added work to it.*

Probe: Can you give an example of how it integrate with your workflow?

- *We use it for data comprehension, for instance at the end of the month and weekly as well. We do our own week report and share the report at the National level, even before then, we also do our stock transaction. The LGAs will send out their balance, we compare with what they should have on the openLMIS and ensure that what we have on the openLMIS is what is on the ledger, and it's also what we have in our physical stock counts.*

5. Are there any features of OpenLMIS you find particularly helpful or unhelpful?
   - *On the stock management, actually 90% of what we do on the openLMIS or even more is around the stock management, that is where we do our issuing, inventory, receiving.*

Probe: Are there any features or functionality that you wish they have added to help perform you daily tasks effectively?

- *There are actually many of such, the truth is that the program is still work in progress, and I believe that this assessment is one of the things too that is going to serve as a feedback to strengthen the job. So there are things that are not where we want them to be. For instance, on the stock management, if you are going to check what is happening with an antigen for instance, you have to check on each of the bin card. So we do not have one place where we can just go and check everything that has been done.*

- *I also have to follow-up with the LGA, and sometimes the summary we get will give us what they have but if we want to do some deep diving to see what transaction they have done we still need to go through the process, but it will be good if we have another tools where we can see the summary of everything in one place. For instance, where we can see the summary of all the antigens in one place.*

- *When we are into the health facility, and it doesn't give you this receipt pass. There is another way you can get your voucher quite alright. But once you send it, and make issuance, you cannot go back on the openLMIS to find the receipts. For instance, if I'm going to receive vaccine from the donor store, they are going to issue a receipt to me, and I'm to issue a receipt back to them that this is what I am receiving. Everything is in one place. Now if I want to issue subscapular receipts in case you need to sort something. But the openLMIC, if I want that receipt, I won't find it. Not only do I have to check antigen by antigen, I have to check batch by batch to get that work done. So it's a whole lot of work. We have actually called their attention to it, and it's good that we are having this assessment so that will be reminded that this is giving us a lot of stress, and they will do something about it.*

Probe: Can you say that the decision support algorithms, alerts, and reminders on the OpenLMIS has helped in improving on your decision making either in stock our or stock in?

- *It has helped, although for Lagos, before the openLMIS, we have the tools that we use and it's, color coded. It shows a maximum, a minimum, and all that just like the openLMIS, but it's not an online tool. And that tool is a weekly one and we don't use it to issue rather, we use it to report our stock balance at the end of the week. So we are able to track what our balance is and make decisions with it. But the openLMIS is better because it's online. It is, a very awesome tool. We are using it on a daily basis.*

6. What factors have enabled you to effectively use OpenLMIS in your work?
   - *The first thing is that it's not an option since it's a national directive. That directive supersede how you feel or any other options you think you have. The second factor I will say is the fact that for Lagos, we use this other tools. So whether there is openLMIS or not, we still need to work on our own tools called weekly dashboard.*

- *Again, you must be very good in the management of your stock at any point in time. One can be in a meeting and the PS call to ask for an important information and you would not like to be found wanting.*

Probe: What are the individual, organizational, environmental, and technological factors?

- ***Individually:*** *I'm used to the computer and a bit sound on it. Although when we started using it, we have some challenges here and there, but we have good support from the national and CHAI. CHAI is always calling on a weekly basis to help us with any challenge while using OpenLMIS.*

- ***Organizationally:*** *As far as Lagos is concerned, it is something that national has mandated, you must do it since its part of our job deliverables. At the national level, after training us they gave us laptops, they train the CCOs at the LGA level and gave them tablets, the PSCs have been informed about it and they continue to monitor and do follow-up while we submit reports weekly.*

- ***Environmentally****: The environmental factor that I see is that you have been conditioned to do your work. You already know that these are your GDP that's been listed and cleared, and this is the environment you work in. You have already been made to understand that once this is your duty papers, as far as you are working under the setting, you must deliver. So you have been conditioned to do what you are supposed to do without giving any excuses.*

- ***Technologically:*** *In terms of technical know-how, that is not an issue for because I can do better on computer.*

Probe: What training and support have you received?

- *We have received support, and we need more support. We need support in terms of capacity building. If you need a training for openLMIS, when you call you will get responses. So in terms of the capacity building I would say there are no lapses because it's been attended to by the national and their partners.*
- *In terms of support for data, that has been a very big deal, that is why at the LGA level, there are challenges. Because when it was introduced, we had data support for 2 to 3 months by CHAI though it wasn't really adequate. We expected that the state will take ownership of the tools, it became a challenge because although the state actually did take ownership of the tools, but when it comes to providing data, that was not directly trashed. In some LGAs, they got data support from their own leadership, but I wouldn't be able to say that was universal. At a point, UNICEF stepped in for 3 months and during that period, the utilization was almost at its maximum, but when the support stopped everything was reversed. In terms of data support even at the state level, it's the same thing.*

Probe: Apart from this online training for individual officers, is there any organized training been conducted over the years?

- *The training has actually been done. For the state's officers, we were trained twice. There was a brief training early 2022, and the main training was done November to December 2022 based on the states, while we also train the CCOs at the LGA level. The training was supported by the National. When we noticed that we're having 1 or 2 challenges, we use our meetings to sort out for data and train our people, and the CCO that have been trained train their people as well, so we train the trainers.*

Probe: How often is this training at the state level?

- *I will say its every day, like whenever they call to report issues they encounter on the openLMIS, train has started already, we don't fix the training because openLMIS is an everyday tool.*

Probe: Do you think there are enough resources directed towards openLMIS?

- *It's a plain no, even as little as data provision because OpenLMIS do not work without data and if we are having challenges with data supply, you can now imagine if any other support is coming. We are not having adequate support, not from state but from the national. Now UNICEF has also come in again they have done three (3) month supply support for us, and I believe it should continue. So UNICEF are trying for us. We are very happy because we now have the strength, the courage, and the grounds to be able to follow-up aggressively with the CCOs. Nobody would give excuse that I don't have data.*

Probe: Can you say its value by those in charge?

- *Honestly, I cannot answer to that. I can only speak for myself. I do not know why it is so. I believe that at every level, we have our different challenges, but I know that the support is not adequate, and I know it's not too late, we can begin to get support today. Thank God that this assessment is going to be helpful.*

Probe: Which organizations are providing funding to system implementation and maintenance?

- *For the funding, I wouldn't know. For training, we have been trained and if they are funding other things, I wouldn't know, mine is to get data stipends because I don't directly get funding for anything.*

7. What challenges have you experienced with using OpenLMIS?
   - ***Individual Challenge:*** *It is better we finish and transaction that day and if by any chance one has to finish it the following day its better one put the right date because that has been the major reason people are having one problem or the other while using the tools, but that is not a challenge for me.*
   - ***Organizational Challenge:*** *The only challenge I have here is data, let them give our people data.*
   - ***Technological Challenge:*** *There is no challenge because we have help anytime one come up, CHAI is always on that tools and anytime we face any challenge they are always available. There is no issue for me when it comes to technical know-how, although some LGAs do have some challenges but they reach out to us and we solve them.*

Probe: Do you feel you have the needed skills and training to use openLMIS effectively?

- *Yes, after I was trained as the state cold chain officer, we trained two (2) of my officers, and since one of my colleagues has other things she attend to in the office, I already have it in mind that I'm going to bring in another officer. The only support we need at this moment is data supply.*

C. Recommendations and improvement

8. What improvements would you suggest to make OpenLMIS more effective for future mass vaccination campaigns as well as routine immunization?
   - *That mass vaccination is a challenge, but for Lagos state is not a challenge because the way it works for us whether you like it or not you must be involved and irrespective of the campaign I always use openLMIS to issue but it's a bit tougher during campaign. What I think can be done is to give as much support as possible, both the support we are*

requesting and the support that the national believe we need.

- *They need to review some of our data tools, some are not necessary and needs to be taken out. For instance, monthly at the LGA level we have VVM3, ledgers, openLMIS, and other tools too all these need to be reviewed.*

- *There should also be a support in the form of incentives because OpenLMIS is not a small work, like yesterday we attended to eleven (11) LGAs, and more activities are been added on the OpenLMIS therefore compensation and yearly award is very necessary to motivate our people especially at the LGA level. Last year after a long training some officers were awarded while they were calling their names the expressions on people showed they really like it and this practice must be replicated at all levels.*

- *Adequate training though this is not an issue for us in Lagos*

Probe: *Have there been any improvement into openLMIS since it was launched?*
- *Yes, the openLMIS is a big deal before the use of openLMIS we send report weekly, when it was launched it become more of the task everybody must use though we still have some data differences at the LGA level but there is always an improvement especially on the capacity building.*

Participant No. 021
Location: Lagos
Respondent's Gender: Female

A. The stock management system and flow of data from paper-based to OpenLMIS

1. Describe your role and the stock management system you currently use for vaccine stocks
   - *I have been in the system as the clinical nurse and have been involved in immunization. I have also worked as the monitoring and evaluation (M&E) officer, so all this work hand-in-hand. My role is* [redacted] *to know our stock balance, when we need to order vaccine, how vaccine are been distributed to the health facilities.* [redacted] *We also monitor the health facility, how vaccine is being used up to the end users, which are the clients. We take stock balance and RISS that is, supporting supervision for both old and new health workers, both for the public and the private facilities. We do refresher training once in a while if we have new information coming on board concerning immunization. We do routine immunization working group meeting and data immunization meeting every month so that we will know how well we are doing in our coverages and, how the vaccines are being put, and the uptake of the vaccine within the community and the health facility.*

2. Describe the flow of data from health facilities to higher levels of the supply management system.
   - *We collect data monthly after their immunization sessions, and the data generated at the health facility level for both public and private facilities are written in their registers and they submit to us every month and it's been uploaded monthly on the template called DQC. I and the M&E compare our data because before it is being uploaded to the DHIS, DQC. All this data are put together and we forward it to the state.*

Probe: In carrying out this tasks don't you use the openLMIS?

- *OpenLMIS is being used for vaccines, but the one that comes to my table is the number of children immunized, then we compare it with the number of vaccine used and it's uploaded on the openLMIS after which we conduct vaccine stock balance for both health facility and the LG.*

Probe: How often were there any discrepancies between recorded and actual stock levels?

- *It's often, that is why we always compare data. The cold chain officer, the monitoring and evaluation officer and I check the data submitted to us by the health facility to know if there is any discrepancies and if there is any, we will go back to the source to compare their data.*

Probe: What do you think can be responsible for such discrepancies?

- *It can be items over reporting or under reporting, but such problem is solve by comparing the number of vaccines being used and what they submitted to us before we now forward the data to the state.*

Probe: Can you now say this is the major challenge you face while taking your stocks?

- *Yes, you know we are human but it's not enormous, we only have pockets of it around and it's usually from both private and public health facilities.*

Probe: can you share any specific examples?

- Yes, we do have day –in-day -out but it's usually resolved before the data is forwarded to the state.

B. OpenLMIS user experience, benefits, enablers and barriers

3. What are the main benefits of using OpenLMIS, compared to the paper-based system?
   - *The openLMIS is a programmed template that is being used and once you login your data you can easily check whether what you input is right or wrong. It's also faster, once you update your data everyone can see it immediately. It's also better because paper can be lost in transit maybe during facility renovation.*

Probe: Can you say give few examples on how the openLMIS has improved your work easy for you to use?

- *It has been helping us to actually know when we are supposed to reorder our vaccine to get more vaccine. It helps us to know when we are having wastages that is too high, It Help us to balance our data that doesn't correspond maybe with the one on the ledger because we still work along with the paper based system.*

4. How easy is openLMIS to us in your daily work?
   - *Based on my interactions with the cold chain officer, it's Very easy. It has made my work easier because I don't have to be in a particular place before I can work on it, once you know your stock balance the template can work on the go.*

Probe: Is there any specific task on your daily work that openLMIS makes easier or harder?

- *OpenLMIS is basically for vaccines stock, it helps to know our vaccine balance at the vaccine store and the LGA level.*

Probe: How well openLMIS integrates with your existing workflow?

- *When it came, it didn't really disrupt our activities, though it was cumbersome combining it with the paper based but it makes our work easier and at some point we got used to it.*

Probe: Can you give an example of such integration?

- *It is basically on our report writing because we do weekly vaccine so it is easier to do our physical counting on the collected vaccines and compare with what we have in the openLMIS.*

5. Are there any features of OpenLMIS you find particularly helpful or unhelpful?
   - *As the LIO, I have not really had a hands on activity on the openLMIS, but I do monitor or speak with the cold chain officer. We have not been trained on handling that template, but we see it when it is being displayed at the meetings, and through some of the CCOs.*

Probe: Can you say that the use of OpenLMIS has helped in improving on your decision making either in stock out or stock in?

- *Yes, especially in terms of our vaccine usages, it prevents vaccine wastages, and also safe us from poor forecasting and improve our forecasting ability. We can be able to project accurately the vaccines needed for immunization activities for the next six (6) month.*

Probe: What is the value of the decision-support algorithms, alerts and reminders has helped with you in stock out?

- *Like I said before, I have not used it but I do watch my CCO while inputting data on it. I know it ease our work.*

6. What factors have enabled you to effectively use OpenLMIS in your work? What are the individual, organizational, environmental, and technological factors?
   - **Individually:** Having a prior knowledge on how to handle the templates, because we use templates a lot. So the prior knowledge about it makes us to work better on the openLMIS.
   - **Organizationally:** It helps us to have a better understanding of what we need and to projects the future vaccine needs.
   - ***Environmentally:*** *The environment doesn't have much effect on the openLMIS because tasks can be perform anywhere.*
   - ***Technologically:*** *our network is still erratic, and based on the complaints, was told that the templates incurred a lot of data to run, and if data is not supplied, it may discourage people that use it. And again, when the network go off any data uploaded at this time will not be showing.*

Probe: How often is the training on the openLMIS and who sponsor it?

- *I don't know, we the LIOs have not undergone any training on that, but the cold chain officers were trained on the use, that is why I said earlier that I have not had a hands on practices on the openLMIS.*

Probe: Do you think there are enough resources directed towards openLMIS?

- *Yes, because the people that are meant to use that package are well trained and they attached mentors to each LGA so that they can be of help whenever there is problem and in case they need more training. In terms of capacity building, it's very robust.*

Probe: Can you say its value by those in charge?

- *Yes they did, because they trained the CCOs on how to use it, and gave out iPads to be used for the work.*
- *They also mentor them, they were providing data for them but it stopped at some points, I don't know if that has been reviewed.*

Probe: Which organizations are providing funding to system implementation and maintenance?

- *I think UNICEF and WHO*

7. What challenges do people experienced while using OpenLMIS?

- *I don't know, maybe the CCO should be able to answer that because I have not used it before. But the complaint I heard is that when data are uploaded, sometimes you open it again and discover that the data you have inputted is not even showing, so you have to go over it again, and also the issue of network.*

Probe: Are you saying by your office, you are not meant to use openLMIS?

- *We have units, the immunization office is a unit which comprises of both the cold chain and the administrative aspect and I believe as the LIO you should be in the known of all these activities in the facility.*

Probe: What can you say is responsible for not engaging the LIOs in the use of openLMIS?

- I don't know the modalities around the openLMIS, I only know that the CCO were trained and LIOs are not trained but if they decide to train us that will be fine.

C. Recommendations and improvement

8. What improvements would you suggest to make OpenLMIS more effective for future mass vaccination campaigns as well as routine immunization?
   - *I want to recommend that the training should be stepped down to the LIOs, M&E and other officers in the facilities so that when there is any problem, we can come together, brainstorm on how to rectify it as this will make the work easier.*
   - *I also want to recommend that data provision should be regular so as to encourage the people using the openLMIS to upload their data as at when due.*

Participant No. 022
Location: Lagos
Respondent's Gender: Female

A. The stock management system and flow of data from paper-based to OpenLMIS

1. Describe your role and the stock management system you currently use for vaccine stocks

   My role as the [redacted]*. I ensure that vaccines are in good condition with the normal temperature range. I also ensure that we use the right carrier to convey the vaccines from the cold store at the NPI unit with needed ice packs. I supervise the cold chain officer and make sure that the vaccines are potent, and have not expired. I distribute vaccines across my*

local government facilities according to their targeted population.

2. Describe the flow of data from health facilities to higher levels of the supply management system.
    - *They always submit to us here at the MPI unit of the local government, and when they submit their data to us, we document it, and input it into the computer, then we send it to the State and from the State to the national.*

Probe: How often were there any discrepancies between recorded and actual stock levels?
- *Once in a while we do have issues where some don't do their stock balance, or maybe they don't bring it on time. The discrepancy maybe as a result of some issues at their facility, but it's not often, it's just occasionally.*
- *This is how we manage our stock, we use their stock balance, the one at our facility and the one they have to calculate what is on ground and to know the amount of vaccines they need when they to our facility.*

Probe: So can you share any specific example?
- *I have just been posted to this LGA, and I can say maybe once but it did not affect anything because we were able to correct it before the end of the month.*
- *We went for the RISS and on checking the vaccines, we saw some vaccines were not recorded, maybe they didn't input them in the stock balance of the previous one, but we were able to make adjustments.*

Probe: You said it occurs once in a while, can you say it's daily, weekly, monthly occurrences?
- The one that I just mention is general view because I just got to this LGA about three (3) months ago, but such occurrences are occasionally.

B. OpenLMIS user experience, benefits, enablers and barriers

3. What are the main benefits of using OpenLMIS, compared to the paper-based system?
    - *I don't know much about the openLMIS because I just became LIO, I have been an assistant so am new in this position.*

Probe: Where you were posted from are they not using openLMIS?
- *They are using it, I think it is easier and faster, It is always with the CCOs, and anytime we need information they can easily get it on the openLMIS.*

4. How easy is OpenLMIS to use in your daily work?
    - *NOT APPLICABLE*

5. Are there any features of OpenLMIS you find particularly helpful or unhelpful?
    - *NOT APPLICABLE*

6. What factors have enabled you to effectively use OpenLMIS in your work?
    - *NOT APPLICABLE*

7. What challenges have you experienced with using OpenLMIS?

    - *NOT APPLICABLE*

C. **Recommendations and improvement**

8. What improvements would you suggest to make OpenLMIS more effective for future mass vaccination campaigns as well as routine immunization?
    - *I think they should carry the local immunization officers along so that we know how to operate it. They should give us maybe like a training on it, just like the CCOs, so it will actually have positive impact on the immunization.*

Participant No. 023
Location: Lagos
Respondent's Gender: Female

A. **The stock management system and flow of data from paper-based to OpenLMIS**

1. Describe your role and the stock management system you currently use for vaccine stocks
    My role is to receive the vaccines from the state, input it into the ledger, then go to the openLMIS to check maybe the state has sent the ID number to openLMIS, if the ID number is not there, I will call so that they can give me the ID number. The ID number app will lead me to the stock balance where I will be able view received vaccines from the state. On the stock management system I currently use, after receiving, I input it into the openLMIS and on Thursday I do physical count after which input it into my openLMIS making use of the issuing voucher.

2. Describe the flow of data from health facilities to higher levels of the supply management system.
    - *After receiving the vaccine, both the private and public health facilities in the LGA will come and receive on a daily basis and by the end of the month we will collate the data. After collation of the data, we use push outside the ending balance, the opening balance, and add everything together and submit to my LIO who then submit the harmonized data to the state.*

Probe: Is there any discrepancy between recorded and actual stock level?
- *If there is any discrepancy which can occur during counting, you have to go to the stock adjustments to adjust and ensure what is on the ledger correspond with what you have on the openLMIS.*

Probe: Is this problem you just mentioned a major one?
- I don't think it is a major problem, there has not been any discrepancies since we do our stock every Thursday.

B. **OpenLMIS user experience, benefits, enablers and barriers**

3. What are the main benefits of using OpenLMIS, compared to the paper-based system?
    - *There are a lot of benefits since we are now in the global system, openLMIS helps us to know the excite number of vaccines that we should have at hand. Another benefit is that its gives accuracy, what we input on it is what we will see.*

Probe: can you give us specific examples of how openLMIS has improved your work?
- *After doing my physical count either every week or every month, I will open the openLMIS to verify what I have there, if it doesn't tally we will recount again, then I will openLMIS and it will direct me to where I should go.*

4. **How easy is OpenLMIS to use in your daily work?**
   - *It is easier, but I can't be using it every day because it consumes a lot of data that is why I do it every Thursday and Friday. We only have some challenges in terms of data.*

Probe: How does openLMIS integrate with your existing workflow?
   - *It didn't disturb me, it is just like a normal procedure. I am not having any issue on it.*

5. **Are there any features of OpenLMIS you find particularly helpful or unhelpful?**
   - *They designed it in a way that is very simple and clear, and if there is any pending issue I will call the state and it will be attended to.*

Probe: Have you called the state store on it before?
   - *Yes, at Amuwo I assigned the work to my assistant so that she can gain experience and she is also computer literate. On getting here, I discovered that my assistant doesn't know much on it so I have to take it as my duty and whenever I have issues I do call the person that left here tin order to assist, that is how I gradually become better on it.*

Probe: Are familiar with all the modules or features on the openLMIS?
   - *Yes, I can say like eighty percent (80%). Since we are not doing program so I can't touch it.*

Probe: Is there any feature that you don't use or that you really use?
   - *That is what am saying, we don't work on the program zone. There is a program feature I don't use like the SIA, I have not touched it before since we are not performing any tasks on it.*

Probe: How has the value of the decision-support algorithms, alerts, and reminders helped you prevent stockouts or make better decisions?
   - *Yes, like if am issuing now and there is a column that I need to 1 but I don't put, it will draw me back if I want to submit until I put the right thing there.*
   - *And again, if I you are doing issuing and you go back to the stock balance that you want to do adjustment it may not go. It will now be asking me if I want to leave the present thing you are doing.*
   - *It also helps me to prevent stock out because anything I click that the batch number has finish it will draw me back.*

6. **What factors have enabled you to effectively use OpenLMIS in your work?**
   - ***Individually:*** *I have to use it since everybody is using it.*
   - ***Organizationally:*** *Because it's mandatory that you must use OpenLMIS.*
   - ***Technologically:*** *It's easier to access but we do experience data issue*

Probe: Can you tell us the training that you have received on the openLMIS?
   - *The one they did was two years ago when they introduced the use of openLMIS, it was physical training but after that they used to give us the training online, we even had one yesterday. There is also a cluster meeting we used to have every month where they draw us back on it so that we can be familiar with it.*

Probe: How often do they call out the training?
   - *Every month, when we go to the state store,* [redacted] *do talk about the openLMIS and also run some training on it.* [redacted] *used to give us an update every weekend. This is where we are, this is where we are going. This is the facility, this is the LGA that have low utilization on OpenLMIS, and this is the LGA that*

has high utilization on OpenLMIS.

Probe: What about online training, who organize it and how frequent is it?

- *The online training is facilitated by* [redacted] *who is on our platform. Like this week, I think they have two days training on it. If you have any complaints, you want to know more about it, they are online.*

Probe: Do you receive support on openLMIS apart from training?

- *They used to send five thousand naira (#5,000), but they said it is for the OpenLMIS data, we received for March.*
-

Probe: It is monthly?

- *Supposed to be monthly but the one they paid us for is March, they have not even paid the previous months. They paid us #15,000 in September and the recent they paid after September was for March which was paid just last month.*

Probe: Do you think the resources directed towards openLMIS is enough?

- *I'm not a computer person so anything you say is welcome*

Probe: Do you think other people value all these resources?

- *It's only the cold chain officer that knows the value of openLMIS*

Probe: Which organization is providing funding to this implementation?

- *For now I know of UNICEF, and they promised to support and I know they will do it. And aside that, I believe that the cluster meeting we are doing every month is been sponsored by same UNICEF.*

7. **What challenges have you experienced with using OpenLMIS?**
    - ***Individually:*** *I don't think there is any issue on it except data, if there is enough data one will be willing to access the tool every day and become guru on it.*
    - ***Environmentally:*** *There is no issue*
    - ***Technology:*** *Yes, there was a time my device was faulty I had to call them and they replaced it for me. And again, openLMIS is consuming a lot of data*

Probe: Do you feel you have the necessary skills and training to use OpenLMIS effectively?

- *They have to retrain us again, we have some new CCOs and ACCOs now, I want them to call us back on it because retraining is always good. They should make the training a residential and they should support us with data because if there is data their will not be any problem.*

C **Recommendations and improvement**

8. What improvements would you suggest to make OpenLMIS more effective for future mass vaccination campaigns as well as routine immunization?
    - *Like I said before more training is needed. We have new CCOs and ACCOs who are to be trained so that we can work together. I will soon be going on leave but I will still be coming to work because of OpenLMIS, but when they train our ACCOs too they will be able to work when the CCOs are not available.*
    - *There should be provision of data, I do buy #4,000 just to be able to access the openLMIS*
    - *On the future mass vaccination campaign there is no barrier once they put us through the needed training*

Participant No. 024
Location: Lagos
Respondent's Gender: Female

A. **The stock management system and flow of data from paper-based to OpenLMIS**

1. Describe your role and the stock management system you currently use for vaccine stocks
   - *As the [redacted] the system has made my work easier to some extent, for instance when I go to the cold store to pick my vaccines, there is a voucher we fill manually but we noticed that after a few months, it fade away but with the use of the openLMIS I can see what I have received and don't have to carry paper about and I can access it once I download it on my phone and can easily refer to it when anyone ask for my stock either for last month*.

2. Describe the flow of data from health facilities to higher levels of the supply management system.
   - *I wouldn't say its excellent because for instance the vaccines that have been issued to me from the state store have their transaction ID, I will receive them and after few days when you want the receive them it will say duplicate which has been our major challenge I will have to report to the state CCO and she will just tell me to ignore.*

Probe: Can we now say that the system was configured in such a way that you can't correct errors from your own end without calling anybody?

- *It has adjustment area, like when you want to write 3,000 received and you type 300,000 there is a place for adjustment you can always go there and give reason why you are making the adjustment.*

Probe: Were there any discrepancies between recorded and actual stock levels?

- *Actually if I don't upload the stock at hand on the system it can't upload itself, so I will be the one to say this is what I have on my stock and I will have to enter the data for the openLMIS to help store it but if I don't do it on a weekly basis I may have stock at hand more than the one on the openLMIS or what is on the openLMIS more than what I actually have. It's a day-to-day activity so that I can have the same figure.*

Probe: Can we now say that these discreteness are human or system error?

- *Yes, they are human error. For instance like me now I don't have an assistant and the work here is a bit overloading for me so I will prefer to have my data at hand ready and start updating it on the openLMIS when am chanced.*

Probe: How often does this happened?

- *It doesn't just happen anytime because it's something you do by yourself, if you don't input wrong data the system can't give you wrong data.*

B. **OpenLMIS user experience, benefits, enablers and barriers**

3. What are the main benefits of using OpenLMIS, compared to the paper-based system?
   - *The benefits are much for instance without the paperwork, I can easily see what I received from the state and what I issue out to the facilities. Without stressing my brain I*

can be able to verify what I have on the ledger and the one on the openLMIS which has given me personal accountability and accuracy.

- *It keeps my record for me because I don't have to carry papers about since everything is already on the data.*

Probe: Can you give specific example how openLMIS has improved your work?

- *It helps me to compare or cross check if the information on the ledger correspond with the one on the openLMIS which has improved my record keeping and accountability skills.*

4. How easy is openLMIS to use in your daily work? Can you say it eases your work on a daily basis?
   - *Yes, it's easy.*

Probe: Do you perform your tasks on openLMIS daily, weekly, or monthly?

- *It's supposed to be on a daily basis as people come to receive the vaccines, as you write it on the ledger you also put it input it in the openLMIS, so it's a daily routine.*

Probe: Can you give me some specific examples how openLMIS has helped improved your work or make it harder?

- *When people come to receive vaccines, it will be registered on the ledger and then input it on the openLMIS and if I am not chanced to do that as a result of workload in the office, I can take it home to input at my convenience, this has made my work to be more flexible.*
- *It also help me to give accurate data information when my superior request for vital information my stock management.*

PROBE

How well can you say that the openLMIS integrate with your existing workflow?

RESPONSE

- It integrates well, even if we do most of our stock management on the openLMIS that doesn't rule out the use of our ledge because the two work hand-in-hand to have the required stock balance.

Probe: Can you give examples of such integration?

- *If I give out vaccines to one of the facilities in my LGA, it will be on the openLMIS and also recorded on the ledge, but if the receiver can't accurately account for it as a result of wrong counting or poor stock management practice, I will check my ledger to confirm if the figure is the same with what I have on the openLMIS.*

5. Are there any features of OpenLMIS you find particularly helpful or unhelpful?

- *All of them have been helpful, because it's designed in such a way that you have your batch number, the expiring dates. So instead of me to start typing, I will just click the one that matches what you receive. However, if there's a way they can help us remove products that have expired or notify us before sending them on the openLMIS. There was a time I received this safety box, while I tried to write the batch number on the safety*

box, it showed me expire products and the safety box was received the previous day. I called the state cold chain officer and she said maybe the batch number I input has expired. They can remove the expired products from it so that we won't start collecting vaccine and it's deactivated or expired.

Probe: Are there any features or functionality that you wish they have added or removed to help perform you daily tasks effectively?

- *It's this issue of duplicates, once a vaccine has been given to your LGA, it's already in your transaction ID and If the same vaccine is being pushed again, I will appreciate it if the application does not accept it. Because you received a vaccine yesterday, and the same vaccine is being pushed to you again and there is nothing showing that the vaccine was received and the system will keep notifying you that you have an outstanding vaccine that has not been received. They should help us work on the issue of duplicate.*

Probe: Can you say that the decision-support algorithms like alert and reminders helped prevent stock out?

- *Yes to some extent, for example, some of my facilities when they come to collect vaccines and they tell me what they have in their stock balance which is higher than what they have received at the end of the month, so I can quickly verify this on the openLMIS and screenshot to them, this has really helped me to make right decision. The reminder on the openLMIS is very helpful, they use to inform me of the sources of the errors I encounter while using openLMIS especially when taking stock.*

6. What factors have enabled you to effectively use OpenLMIS in your work?

- ***Individual:*** *It's not time bound that I must work in the Office, so makes my work easier.*
- ***Organizationally:*** *I would say it's also good because once I receive my vaccine and any facility tell me they are stock out of vaccine I can easily verify it so it gives us clear view of what we have in our MPI store.*
- ***Environmentally****: This environment is also good*
- ***Technologically:*** *it's also good because when it comes to data It's what you input that you will see.*

Probe: What training and support have you received?

- *Yes, I have training on this job, though it's a 1-on-1 training with one of my boss, because I wasn't part of the major training. I used to be an assistant LCCO but when my boss left I was made the LCCO so the training was stepped down for me. And subsequently, if I have any question, I would call him and he will put me through.*

Probe: How often is the training on the openLMIS and who sponsor it?

- *I can't really say anything about it since am new in the system*

Probe: Do you think there are enough resources directed towards openLMIS?

- *I will say yes because my MOH here actually bought this airtel 5G router because the issue of data has been the most challenging. When we told him that data is one of our*

challenges here, he bought the and he's been funding it, so even if I don't have cash on me, I know that when I get to the office, I can actually open my tab and do my work.

Probe: Can you say its value by those in charge?
- *I know they will be supporting, but I can't speak for them in terms of value.*

Probe: Which organizations are providing funding to system implementation and maintenance?
- *I know of CHAI, NPHCDA, WHO and also heard of Emirate.*

7. What challenges have you experienced with using OpenLMIS?
   - ***Individual Challenge:*** *My major challenge is that the tool is too long, especially when you are to do physical stock. Most of the one that is written deactivated should have been removed by now. And sometimes, you already have a zero stock on some particular antigen, they should remove them instead of giving us very long work to do.*
   - ***Organizational Challenge:*** *The only challenge I have here is that I don't have an assistant because I'm new as the LCCO. So they are still trying to work out an assistant for me and the tasks before me is much*
   - ***Environmental Challenge:*** *I don't have any*
   - ***Technological Challenge:*** *I will say no because data is available and he phone is working well.*

Probe: Do you feel you have the needed skills and training to use openLMIS effectively?
- To the best of knowledge, I will rate myself 85% on the openLMIS.

C. Recommendations and improvement

8. What improvements would you suggest to make OpenLMIS more effective for future mass vaccination campaigns as well as routine immunization?
   - *They should organize trainings for the new CCOs that newly joined*
   - *In terms of distribution, there are some health facilities in some wards with large TP because of their population, if there is a way the openLMIS can put their TP during campaign so that we can know the expected or target population.*
   - *The openLMIS should also be stepped down to the health facilities because they don't need to enter their own into digitally. They have this nonchalant attitude, Instead of them coming down to collect vaccines they will say they want to manage the ones they have and the ones they think are available may not be enough. So it should be design in such a way that I can also monitor what they have in stock.*

Probe: Have there been any improvement and investments into the openLMIS since it was launched?
- *I can't really say much on this because I don't know the policy guiding the investments, but if am to talk about the usage of the App I can say there has been an improvement*

Participant No. 025
Location: Lagos
Respondent's Gender: Male

A. The stock management system and flow of data from paper-based to OpenLMIS

1. Describe your role and the stock management system you currently use for vaccine stocks
    - [redacted] I use OpenLMIS for Vaccine accountability and there are other tools we use, we also rely on hard copies which is the ledger. When people come for vaccination, we register their information in the ledger and then input into the openLMIS. CHAI first introduced the openLMIS seven (7) years ago but was later dropped because of data related issues and Lagos state was the first to experience it, although it was later stopped before its re-launched three (3) years ago and was adopted by other states.

2. Describe the flow of data from health facilities to higher levels of the supply management system.
    - *When we receive vaccines from the state store, people come to collect them in our LGA facility. We record it in our ledger before it's been transfer to the openLMIS. And in cases whereby the vaccines received do not match the figure we give out, we do the adjustment on the openLMIS which can been seeing by everyone at local and national levels.*

Probe: How often were there any discrepancies between recorded and actual stock levels?
- *It used to happen and it usually on stock balancing, but it's not a regular occurrence. You can't whether you have discrepancy or not until you open the openLMIS.*

Probe: Can you give any specific examples?
- *Yes, it's usually when taking stock. The variation in figure on stock in hand and that on the openLMIS could be as a result of wrong counting or internet problem while updating data on the openLMIS. This is not a regular thing but can happen anytime.*

B. OpenLMIS user experience, benefits, enablers and barriers

3. What are the main benefits of using OpenLMIS, compared to the paper-based system?
    - *It has a lot of benefits because it helps us save our data, as you can see now, our office leaks anytime rain falls; it can destroy our ledger thereby lose vital information on it. However, it also added to our problem because we don't have supporting hands. We will treat ledger and the openLMIS at the same time, so openLMIS added to our workload.*

Probe: Can you give specific examples of how openLMIS has improved your work?
- *It helps me keep my data and also improved my accountability.*

4. How easy is OpenLMIS to use in your daily work?
    - *It's easy to use as long as there is no internet issue, as you can see that have been unable to login since you came in*

Probe: What specific tasks in your daily work that openLMIS makes easier of harder?
- *While taking our stock balance, openLMIS gives us directives on what to do especially when there is an error while working on physical inventory.*

Probe: How well does the openLMIS integrate with your existing workflow?
- *It doesn't integrate because it gives us multiple data entry task to perform, we will work on the paper and on the openLMIS, so we keep repeating everything every time even while writing our monthly report, and everything is going to the same place.*

5. Are there any features of OpenLMIS you find particularly helpful or unhelpful?

- *Everything is useful and helpful because when you have any issue you must visit openLMIS.*

Probe: Are there any features or functionalities you wish openLMIS had for you to perform your daily tasks?

- *No, everything is fine for me.*

Probe: What value of the decision-support algorithms, alerts and reminders during stock-out while using openLMIS?

- *It helps me to know where the error is and to make adjustment*

6. What factors have enabled you to effectively use OpenLMIS in your work?
    - ***Individual:*** *It makes my work easier for me and keep me record because I can't be carrying my ledger everywhere I go, but with the openLMIS I can access information anywhere.*
    - ***Organizationally:*** *It links us with other LGAs and even the state, and it's easy to access.*
    - ***Environmentally:*** *OpenLMIS is safe, like I said before, this place is leaking whenever it rains and that can lead to damage of data on the ledger.*
    - ***Technologically:*** *The tablet is easy to carry about and information on it is accessible*

Probe: Describe the training and support have you received to enable the implementation of openLMIS?

- *I have done training on it once, but there is always an individual meeting on zoom and one can attend it as many times as he or she wants. we attend like two or three times.*

Probe: Is there any joint training for the cold chain officers like you?

- *No joint training, at times when we go for our technical training or meeting, the state cold chain officer attend to issues around the openLMIS.*

Probe: Who implement the training?

- *WHO, UNICEF, CHAI, NPHCDA and also the state.*

Probe: Do you think there are enough resources directed towards openLMIS?

- *No, because openLMIS is consuming a lot of data and they are only giving us #5,000 which is not even regular often times we use our money to buy data.*

Probe: Can you say its value by those in charge?

- *They value it just that they are not doing well on the aspect of data supply*

Probe: Which organizations are providing funding to system implementation and maintenance?

- *NPHCDA UNICEF, WHO, CHAI.*

7. What challenges have you experienced with using OpenLMIS?
    - ***Individual Challenge:*** *It increases my workload, I will prefer using paper-based system.*
    - ***Organizational Challenge:*** *They do not give us money for data regularly, we are only left to our faith.*
    - ***Environmental Challenge:*** *There is no challenge that is environmental for me.*
    - ***Technological Challenge:*** *This is a regular issue, you witnessed how long it took me to openLMIS, but it could be as a result of our location.*

Probe: Do you feel you have the needed skills and training to use openLMIS effectively?

- *Yes, but they can still train us more on it.*

C. Recommendations and improvement

8. What improvements would you suggest to make OpenLMIS more effective for future mass

vaccination campaigns as well as routine immunization?

- *Adequate training for all personnel and also recruit more people to do the job because the workload is too much on us. They should also provide funding for data regularly*

Probe: Do you think there has been an improvement and investments into the openLMIS since it was launched?

- *Yes, and we hope for better investment so that we also can be compensated.*

Participant No. 026
Location: Lagos
Respondent's Gender: Male

A. OpenLMIS and its role within the EPI Programme and beyond

1. What was the rationale for the rollout process of OpenLMIS for COVID-19 vaccination and subsequently Routine Immunization (RI) implementation?

- In public health, data is key; the OpenLMIS has made the data collection, harmonization and use for action easier for everyone. Rather than relying on paper-based and word of mouth mechanisms; It provides the data and dashboards that helps to make informed decisions; It's a step in the right direction.
- It's more robust now and encompassing than it was for just COVID; but it's the same principle being used in operations.

2. Describe the role OpenLMIS currently plays within the EPI program.

- The platform provides information that helps to make informed decisions without having to make several calls; it's been helping to monitor vaccine push/logistics and accountability. It's more or less a decision-making platform for me regarding vaccine safety and logistics.
- The CCOs that do more on the platform; but I act on information that is pulled from it. From the dashboard it's clear what needs to be done in terms of stock levels and needs. It guides on asking the right questions to prevent stockouts. Ideally I use on a monthly basis; but I do have random check-ins based on what I've seen previously

3. How has OpenLMIS impacted your decision-making and program management?
   - Asides having real-time information for decision-making; it has helped to identify my officers at the lower levels that need more capacity building on electronic system reporting.

4. To what extent is the OpenLMIS interoperable with other information systems (DHIS2)?

-

5. Is the OpenLMIS already being used in other health areas (i.e. for other health commodities)
   - To the best of my knowledge; its just within the immunization program.

B. Barriers and facilitators to adoption, data quality, and use of OpenLMIS

6. What are the main barriers to the adoption, use, and data quality within OpenLMIS?
   - Capacity building was a real issue for adoption; it took a lot of training and retraining to understand how the system works.

- Data; the system requires a lot of data; there was no allocation for funding this at the beginning and we had to rely on donors; it's still an issue now.
- The devices (laptops and tablets) are beginning to breakdown and has led to gaps in reporting.
- **Also, there have been cases of downtime of the platform, that has affected reporting timeliness**
- As an organization; we've embraced the OpenLMIS; the challenges are more operational challenges.
- The issues have not been gender specific; although there are more females in the system in Lagos so absolute numbers may give a bias for females; The bulk of the capacity issues are more related to staff turnover where trained officers move out of the system.
- Everyone is held to the same standards, there are no traditional roles influencing the staff responsibilities.

7. What factors have facilitated the successful implementation and use of OpenLMIS?

- Willing and receptive CCOs have been essential to the success of the OpenLMIS implementation.
- Technical support from the partners and NPHCDA has been very crucial.
- No other organizations are supporting asides CHAI and UNICEF.
- Resources are never enough; capacity building needs to be more frequent and the supply of funds for data needs to be improved on. The platform tends to consume a lot of data.
- We did get some support from UNICEF for monthly meetings with the CCOs; and what we do is to integrate the trainings in the agenda of those meetings; CHAI is also a part of those meetings with other technical officers.

C. Perceptions on the link between OpenLMIS data use and immunization coverage

8. Have you noted any links between OpenLMIS data use and immunization coverage, completion, and timeliness?
   - What I pick out easily is to use the OpenLMIS data to validate data that I get from the service delivery; I use it more for data validation. I also look at wastage. I believe it has contributed to improved coverage overtime. It helps to have a deep-dive to the immunization program; where we are able to improve processes and eventually should improve coverage. it's part of a chain of events.
   -

9. Has the use of the OpenLMIS led to any unexpected effects on vaccine delivery or staff roles? Describe these changes.
   - There's been none.

D. Learnings and recommendations from OpenLMIS utilization for COVID-19 vaccination

10. What unique lessons were learned (positive and negative) from using OpenLMIS for COVID-19 vaccination, and what recommendations do you have based on these lessons that can be applied to future large-scale campaigns and outbreak response in Nigeria or other countries?
    - One of the major challenges that kept popping up was the OpenLMIS downtime and that affected real-time data during the COVID-19 Vaccination; but now this is not a frequent

event.
- OpenLMIS provides adequate visualizations that can be used for any kinds of campaigns to manage the supply chain.
- Downtime and challenges with data should be resolved
- Access should be a bit broader; The program managers should be given access to create visualizations.
- Upgrading the system now might not be a good idea; unless it is been simplified.
- The system should consume less data.

Participant No. 027
Location: Lagos
Respondent's Gender: Female

A. OpenLMIS and its role within the EPI Programme and beyond

1. What was the rationale for the rollout process of OpenLMIS for COVID-19 vaccination and subsequently Routine Immunization (RI) implementation?
Using it for COVID first, was to see how it will be utilized at the state or LGA levels, generally speaking and how the app will be effective or not; to see if data upload will be seamless, and also to see if the objective will be met, to have real-time data.
I think it's the same – It's just daily utilization and having the stock balance(s).

0. Describe the role OpenLMIS currently plays within the EPI program.

The role is well spelt out; however the challenge is around the upload of data real-time by the officers; there are still challenges here and there especially with the new officers as CCOs who are still trying to grasp how to use the app. For RI, it helps to follow up with the stock real-time and if there's any error; it is quickly picked up on and there's an alert to the state to balance what's been reported vs the physical stock-count.

We see the stock levels at the LGA levels and it helps to see the stock situation (minimum, maximum and overstock) and helps to follow up with the LGAs to address the situation; sometimes, it might be a data error, but then it helps to query why the situation is the way it is; especially as the LGAs operate a pull mechanism from the state store.
For SIAs, we have not had to use the OpenLMIS.

0. How has OpenLMIS impacted your decision-making and program management?

The app itself is a good one; but because of the challenge of data uploads; we have not really being using it for decision-making; although it helps to identify stock level situations that need to be addressed. We use DHIS2 for also stock level and utilization to support decision making.

B. Barriers and facilitators to adoption, data quality, and use of OpenLMIS

0. What are the main barriers to the adoption, use, and data quality within OpenLMIS?

- *Probe for any specific user groups (Cold Chain officer/Program manager, male or female) within the program/supply chain who have difficulty adopting or using OpenLMIS effectively, and why*

The LCCOs are the ones with the challenges; at the State level its great but at the LGA level; some of the officers need to be trained and retrained; and sometimes it's the network issue and when they re-enter the data it leads to double-counting and is seen as overstock; availability of data (for internet connectivity) is also another issue as the CCOs don't usually have data. The OpenLMIS is data consuming as well. The program officers have a lot of data tools to enter, and becomes overwhelming at the end of a work week. There's no support for data at the state level; and data is being provided out-of-pocket by the officers. Although, there's advocacy from the district and LGA for support; but the challenge still persists.
There's been no gender differences noted; usually, where there's support, you'd see more productivity.
Some of the tabs given were not functional at some point and were returned to the national; especially in Eti-osa.

As regards gender equity, Lagos provides equal opportunity; there's been no gender equity issues; It's been all about the dedication to the work that matters.

- *Probe for any traditional gender roles and stereotypes within organizational facilities, and how these impacts can be mitigated*

It's been less about gender and more about years of experience and competency; most officers are females now and some of the older officers have taken more senior roles and the younger hands are doing quite well.

7. What factors have facilitated the successful implementation and use of OpenLMIS?
There have been sanctions and several notifications to the districts; where they sign and undertaken and were required to come to the state store to then enter the data before collecting their vaccines; and they were reported if they were not done. This is been done less now that reporting has improved.

CHAI supported at the beginning, UNICEF also supported at the beginning, but no other organizations are supporting.

It was just a one-off training and then the state technical CCO meeting happens every week and the state CCO is available for one-on-one interaction on the use of the app.

IGA(Inventory Gap analysis) is another app that's being used at the State and LGA level. This app helps for CCE tracking and monitoring.

C. Perceptions on the link between OpenLMIS data use and immunization coverage

0. Have you noted any links between OpenLMIS data use and immunization coverage, completion, and timeliness?

I haven't looked into that, but I think it's better with monitoring as we are able to manage vaccine wastage better. I think there's been a decline in the coverage, although it has nothing to do with the OpenLMIS. Although, it has helped at LGA level for reporting as the LIO and LCCO can also compare vaccine utilization and children immunized.

0. Has the use of the OpenLMIS led to any unexpected effects on vaccine delivery or staff roles? Describe these changes.
I think that it has affected the CCOs, in the sense that they have a lot of data tools to be used and it's quite challenging for them to also then work with the OpenLMIS; and because they don't have enough support; you don't see many people motivated to do the work as there's now more

work to do. There's always just one officer and then an assistant that may be new and may not know what the app is all about.

D. Learnings and recommendations from OpenLMIS utilization for COVID-19 vaccination

0. What unique lessons were learned (positive and negative) from using OpenLMIS for COVID-19 vaccination, and what recommendations do you have based on these lessons that can be applied to future large-scale campaigns and outbreak response in Nigeria or other countries?

It wasn't easy to adopt. And there is no much political buy-in, nor policies that facilitated the adoption; the trainings helped (one-off from the national and the frequent on the job support from the State CCO). We just have dedicated personnel that are also willing to get the work done even with OOP expenses. There are exceptions where the MoH provided modems in Mushin and would be provided data, monthly, although it's not clear where the funds come in from.

If the app can be simplified; it would help. The app can be redesigned to a form where it is not consuming so much data. refresher training are also needed asides the support that CHAI provides;

The tabs provided are not durable; there have been cases where they had to fix them. However, laptops will be better.

Participant No. 028
Location: Lagos
Respondent's Gender: Female

A. The stock management system and flow of data from paper-based to OpenLMIS

1. Describe your role and the stock management system you currently use for vaccine stocks
   - [redacted] *We both determine our monthly stock before we make requisition, and ensure that the vaccines are well stored, and also distribute vaccines to the facilities within our target population. We monitor to ensure that vaccines are potent till they get to the last consumer. We do physical stock count at both LG and facilities to ensure accountability.*

2. Describe the flow of data from health facilities to higher levels of the supply management system.
   - *We still have some itches. It's not yet smooth because we still have to do manual counting before the data is being updated on the openLMIS.*

Probe: How often were there any discrepancy between the recorded and the actual stock level?
- *There is no discrepancy, what I'm saying is that sometimes there are itches and we ensure that we rule out discrepancy which is as a result of knowledge gap at the facility level that is PHC.*

B. OpenLMIS user experience, benefits, enablers and barriers

3. What are the main benefits of using OpenLMIS, compared to the paper-based system?
    - *OpenLMIS makes accountability very easy, though I do immunization and I don't work on openLMIS. The main person that can really talk about our challenges when it comes to OpenLMIS is CCO because we only oversee activities and share our report.*

Probe: You mean there is no area that OpenLMIS can improve your own work?
- *It will improve my work because my own reporting template captures everything. So if OpenLMIS has made our work easy, it validates what I'm reporting for the whole LGA.*

4. How easy is OpenLMIS to use in your daily work?
    - *I don't report on a daily basis, I report on a monthly basis and since she has validated what she has with OpenLMIS, she confirms with me. It makes my work easy and I submit my report on time.*

Probe: How well the openLMIS integrate with your existing workflow?
- From what I have seen, OpenLMIS is like added responsibility to what is existing. It takes time because they have to work with data, they have to spend more time on the system and all that. So from the reports I get from them, it's like it has increased their tasks because we have not totally migrated to OpenLMIS.

5. Are there any features of OpenLMIS you think can be helpful or unhelpful?
    - *I don't work on it I only visualize when my CCO is on it, we are not even trained on it as LIOs.*

6. What factors have enabled you to effectively use OpenLMIS in your work?
    - *Not Applicable*

Probe: How do you think OpenLMIS will help you perform your tasks apart from visualizing?
- *It will help my stock, I have the vaccine, the VVM, and the number of doses, with OpenLMIS I know that my patient is getting the right thing. .*

7. What challenges have you experienced with using OpenLMIS?
    - Not really because my role is to ask her how far she has gone on the openLMIS

C. Recommendations and improvement

8. Do you think openLMIS would help improve the future mass vaccination campaigns as well as routine immunization?
    - *Of course, you know every program has its logistic system. And for the pharmacy unit, they have their RH. And looking at the results, the way it has improved their work, I believe that OpenLMIS will improve our work.*

    - *There should be more training cascading maybe to heads of facilities and those that are seeing stock at the facility level, and should not be limited to the CCOs alone just like DHIS for immunization reporting which is not limited to the M & E officers since some officers can as well send their repot. In this way, the senior officers will just verify and validate which will make the work easy.*

- *I believe that OpenLMIS can also enhance service delivery and future mass immunization campaigns if the right people are trained for the job..*

Participant No. 029
Location: Niger
Respondent's Gender: Male

A. OpenLMIS and its role within the EPI Programme and beyond

1. What was the rationale for the rollout process of OpenLMIS for COVID-19 vaccination and subsequently Routine Immunization (RI) implementation?

   Well, the openLMIS as a software, was a child of necessity within the Nigerian immunization system, because at a point we were using an LMIS system before, which was online, and it provided visibility, of vaccine stocks across several storage levels. However, there was a time of transition where we had to move back to Excel based documents, which had some limitations. So, the nation, as a whole thought to bring something that is more user friendly, that will, restore that visibility. So openLMIS is here to provide us with visibility of the vaccine situations in every storage location, show evidence of transaction, the movement of vaccines from the National all the way to the facilities, the accountability, the reporting aspect of it.

2. Describe the role OpenLMIS currently plays within the EPI program.

   OpenLMIS plays the role of data storage for managers to work with using vaccine data availability to plan effectively and efficiently and allocate needed vaccine to the program.

3. How has OpenLMIS impacted your decision-making and program management?

   Well, it makes everything easier. Like I said, the key word there is the visibility and in terms of planning, you know, when we look at the dashboards, when we look at the feedback, we know where we have stockouts for social antigens and how that may affect the routine immunization and the EPI in general, in terms of, missed opportunities and what not. So, those reports that are coming in play a very key role in influencing, decisions, movement of vaccines from one location to another.

4. To what extent is the OpenLMIS interoperable with other information systems (DHIS2)?

   I haven't been in vaccine management for a while, but I was aware there was a training sometime. I think it was earlier this year, where there was a talk of integration between the OpenLMIS and the DHIS2, talking about try360 where there will be, a kind of triangulation, because even on the DHIS2 there's a vaccine management aspect, where utilization reports are being sent. So, the national, I believe, thought to sort of integrate the two platforms, so the data coming from the DHIS2 and the stock data on the OpenLMIS can be harmonized. So that is basically what I know of that.

5. Is the OpenLMIS already being used in other health areas (i.e. for other health commodities)

   The OpenLMIS can handle the vaccines, both routine and supplementary, including the covid 19 vaccines. It has an aspect for the cold chain equipment, where stores can enter their cold chain inventories and it also has a component for other commodities, such as consumables, nutrition products. those are also a component within the OpenLMIS

B. Barriers and facilitators to adoption, data quality, and use of OpenLMIS

6. What are the main barriers to the adoption, use, and data quality within OpenLMIS?

   Well, some of the major barriers are. In some places, it's network access. You know, we know the situation. We know how the network coverage is within the country and sometimes even within the metropolis. Sometimes you have network outages. So that is one major barrier in terms of reporting. And then there's also capacity, because we have, EPI Managers coming and going. you have experienced people and then, due to transfers, retirement and what have you, you have new people coming in. Those transition periods where those people do not have the adequate capacity to fully operate the system then in some cases, there's also, what we call health worker attitude, the person is capable, but you know the attitude people just need extra push sometimes to do what they are supposed to do. So, these are some of the major barriers.

7. What factors have facilitated the successful implementation and use of OpenLMIS?

   The technology aspect of it has been taken care of, by the system. Because you know, when you introduce a system such as this, which is web based, you know you may have complaints here and there that I do not have. the technological equipment, such as a laptop, such as tablet or a phone, but that has been taken care of every LG CCO, that is the local government coaching officers, all 774, we issued with tablets, new tablets. The state coaching officers were given tablets as well. At this level, we have laptops. We use, everybody is equipped. In terms of technology, the knowledge, is there? The know, how is there? We've been trained properly.

C. Perceptions on the link between OpenLMIS data use and immunization coverage

8. Have you noted any links between OpenLMIS data use and immunization coverage, completion, and timeliness?

   Yes. I think there's a direct link there. I keep mentioning the visibility now because prior to the implementation of the OpenLMIS, that visibility wasn't there. So, if there's a stock out, in state A before that is being flagged up at the higher level where decision can be taken to replenish that state with the required antigens, it takes a bit of time. But now the visibility is there, even if the reporting is not happening, which is on a weekly basis, you know, is a very short time frame that we know. Okay, on each this week, we know where you have. We are

tracking where so we had, we have had significant reduction in the rate of expiry of vaccines. That is one because we are closely tracking the VVM status, the expiries, and then, more importantly, the stock out aspect of it, which is the part that affects coverage now, because when you have stock out, you have missed opportunities. And by the time that opportunity is missed, in some cases, those people never come back, but that is closely being guarded, as long as soon as the stock out is being noticed, as long as there is vaccines, that storage location is quickly replenished, so I believe that will have an impact in terms of immunization coverage at various levels.

9. Has the use of the OpenLMIS led to any unexpected effects on vaccine delivery or staff roles? Describe these changes.

   No, nothing to be honest, because the utilization aspect of it, whether request is formally made through the platform or manually. The system has that flexibility. If I'm requesting vaccines from you, the system provides an Avenue for me to do that within the system, and if I fail to do so and I bring a paper-based request, the system also allows me to manually enter that request and then there will still be evidence. So, no matter how you look at it, the system is flexible that whether anything unexpected or course we could still do shipments without notification sometimes because due to the maybe urgency of it, there's no time to follow the due process so, the system is flexible to accommodate.

D. Learnings and recommendations from OpenLMIS utilization for COVID-19 vaccination

10. What unique lessons were learned (positive and negative) from using OpenLMIS for COVID-19 vaccination, and what recommendations do you have based on these lessons that can be applied to future large-scale campaigns and outbreak response in Nigeria or other countries?

    Well, I believe during the during the COVID era, which was you know the when the OpenLMIS was pioneered one of the major lessons that we saw or happen is during that time was people are not really used to the system at that point. So, there were a lot of challenges in terms of updating in terms of reporting. For the for the OpenLMIS, but I believe now with the transition into the routine immunization and the adjustment of the reporting rates because then I believe the reporting was a lot shorter than the week during the COVID during the COVID module, I believe it was almost daily and that was a bit stressful for those people given the workload and other responsibilities that they had. But now you know with the transition and the life, relaxation of the reporting period things is a lot smoother now.

Participant No. 030
Location: Niger
Respondent's Gender: Male

A. The stock management system and flow of data from paper-based to OpenLMIS

1. Describe your role and the stock management system you currently use for vaccine stocks

- [redacted] ensuring that vaccines are stored and handled under normal temperature controls to maintain their efficacy. This involves coordinating with various stakeholders to ensure timely and secure delivery from these zonal cold chain storage facilities to local healthcare centers with equipped facilities and also using the Logistics management system (the OpenLMIS) to monitor real-time inventory

2. Describe the flow of data from health facilities to higher levels of the supply management system.

- Well, from what we have been practicing here, I can clearly say that the flow of data from health facilities to higher levels of the supply management system begins at the point of shipment delivery, where health workers collect and record data on vaccine stocks, usage, and wastage using standardized tools such as the OpenLMIS. This data is then collected at the facility level and submitted for the intended purpose for example, sometimes I request will be sent from the ministry for use provided the vaccine utilization records typically on a monthly basis which we solely rely on the OpenLMIS for such information but you are aware the frontline officer don't have access to OpenLMIS, so we reach out to LGAs officers compile the paper-based data and do physical stock count, identify potential shortages, and track vaccine utilization and update their OpenLMIS. This consolidated data through the OepnLMIS is what we present to the management, where further aggregation and analysis occur,
- Cold chain officers and Managers review the data to ensure accurate forecasting, planning, and distribution of vaccines. Finally, the data reaches the national level, where it is used to inform policy decisions, optimize the national vaccine supply chain, and ensure alignment with global targets. Throughout this process, the OpenLMIS plays a crucial role in facilitating real-time data reporting, analysis, and decision-making.

B. OpenLMIS user experience, benefits, enablers and barriers

3. What are the main benefits of using OpenLMIS, compared to the paper-based system?

- To be honest, the benefits it not measurable because OpenLMIS compared to the paper-based we were using is multifaceted, enhancing the overall efficiency and effectiveness of the supply chain. Firstly, OpenLMIS significantly improves data accuracy and timeliness by reducing human errors associated with manual data entry and enabling real-time reporting. Automated calculations and data validation ensure precise inventory tracking and facilitate more informed decision-making.
- Secondly, OpenLMIS offers enhanced supply chain visibility and management through the platform. This allows stakeholders at various levels to share and analyze data seamlessly, improving demand forecasting, resource allocation, and tracking of vaccine movement through the supply chain. Such visibility helps in identifying bottlenecks and addressing them promptly, thereby ensuring a steady supply of vaccines.
- Lastly, OpenLMIS increases efficiency and cost-effectiveness by streamlining processes and reducing the labor and time required for data collection, reporting, and analysis

unlike before when we get complaints about allowances. The system's ability to optimize resources minimizes wastage and prevents overstocking, leading to significant cost savings. Additionally, OpenLMIS is scalable and adaptable, capable of evolving with the needs of other health programs and integrating with other management systems, making it a sustainable and future-proof solution, I am personally encouraging some of the programs I know are still operating paper-based should adopt OpenLMIS model.

4. How easy is OpenLMIS to use in your daily work?

   - OpenLMIS platform has improved the management and distribution of our commodities, including vaccines. Its user interface is generally intuitive, which makes my daily tasks like tracking inventory levels, managing orders/stock, and generating reports straightforward
   - All I can say is that it has made my work simple and easier.

5. Are there any features of OpenLMIS you find particularly helpful or unhelpful?

   - Well all the features on the OpenMIS have been helpful most especially Inventory Management and easier to generate reports, as for the unhelpful feature is the Dependence on internet connectivity, which has been a challenge in remote areas we can incorporate the offline features

6. What factors have enabled you to effectively use OpenLMIS in your work?
   - When considering the factors that have enabled the effective use of OpenLMIS as the Zonal Cold Chain Officer in the vaccines space here in Niger state, I would say it has to do with the board features included on the management system which are User-Friendly Interface, online based Data Access and other support

7. What challenges have you experienced with using OpenLMIS?

   - *We don't have many challenges here but we can encourage continue data allowance support and training for new staff*

C. Recommendations and improvement

8. What improvements would you suggest to make OpenLMIS more effective for future mass vaccination campaigns as well as routine immunization?
   - The platform should be like other MIS platforms. When it comes to Quick Access. Somebody you can just log in and access it and any data that you have, as you click it, user-friendly, and the dashboard should be able to summarize data and information. Eg, expired vaccines, facilities with Low stock, and stock rate. Generally, the dashboard should pop up anything that needs attention as against what we have now. A blank dashboard and lastly, provision for data for internet access since the OpenLMIS required real-time monitoring

Participant No. 031
Location: Niger

Respondent's Gender: Male

A. **OpenLMIS and its role within the EPI Programme and beyond**

1. What was the rationale for the rollout process of OpenLMIS for COVID-19 vaccination and subsequently Routine Immunization (RI) implementation?

   - OpenLMIs is a logistics management tool that was designed and approved by the National Primary Health Care Agency, and this was disseminated to the states. And the rationale, basically around it was because of the expansion of the vaccine space and also proper vaccine management, because we have what is a lot of vaccines are procured or donated to Nigeria annually by different funders, basically, and these vaccines are also transported to states, LG and also facilities. If you want to look at the facility, essentially primary care facilities. In Nigeria, we have over 40,000 primary care facilities. So look at the quantum of vaccines. And these vaccines are also expensive. So essentially, the rationale behind it was for proper vaccine accountability, management, and also the logistics for it

2. Describe the role OpenLMIS currently plays within the EPI program.

   - So, the EPI (Expanded Program for Immunization). Some people call it the NPI national program for immunization. However, this year is 2024. Is 50 years since EPI was launched in Nigeria, so essentially, this open LMIS plays a very, very vital role because it is simple, We have a principle or one of the commandments of vaccination that when there is no vaccine, there is no immunization. So essentially, the open LMIS has to come into play, because it is a tool that is electronic computer based that was utilized in the quantification and the distribution and also. Uh monitoring the utilization of the vaccines in the front line, and feedback is provided from that front line back to the state and up to the national level, which is also used for decision making. The data generated.

3. How has OpenLMIS impacted your decision-making and program management?

   - OpenLMIS has significantly impacted our decision-making and program management by providing real-time visibility into inventory levels, stock-outs, and order statuses. This allows the possibility to make informed decisions quickly, ensure the continuous availability of essential Vaccines, and coverage, and reduce wastage through better forecasting and planning.

4. To what extent is the OpenLMIS interoperable with other information systems (DHIS2)?

   - OpenLMIS is been validated by DHIS2. This interoperability enables seamless data exchange and integration, facilitating a more comprehensive view of Vaccination space performance and supporting coordinated efforts across. With both the DHIS2 and OpenLMIS, we can have clear coverage of how vaccines where distributed and utilized

5. Is the OpenLMIS already being used in other health areas (i.e. for other health commodities)
   - Yes, OpenLMIS was being used for managing other health commodities beyond Routine immunization that was during Covid but for other health commodities aside RI and Covid it will be a welcome idea so that the health space can have on central data center

B. Barriers and facilitators to adoption, data quality, and use of OpenLMIS

6. What are the main barriers to the adoption, use, and data quality within OpenLMIS?
   - Technical Challenges: Unreliable internet connectivity in some areas and lack of data allowance.
   - Training and Capacity: Insufficient training and support for new staff on using OpenLMIS effectively.
   - Data Quality Issues: Inconsistent data entry and reporting practices leading to data inaccuracies.
   - Resistance to Change: Reluctance from staff to adopt the systems and workflows due to poor logistics and allowances.

7. What factors have facilitated the successful implementation and use of OpenLMIS?

   - Well, I believe the Strong Leadership and Support with Commitment from government and health program leaders.
   - Frequent engagement meetings with officers (during the technical working group) to understand their challenges and address them
   - Identify competent personnel that are reliable to do the job effectively

C. Perceptions on the link between OpenLMIS data use and immunization coverage

8. Have you noted any links between OpenLMIS data use and immunization coverage, completion, and timeliness?
   - Many users [redacted] have noted positive correlations between the use of OpenLMIS data and improvements in immunization coverage, completion rates, and timeliness compare to paper based version.
   - They are now providing accurate and timely data, OpenLMIS has helps ensure vaccines are available when and where they are needed, reducing missed opportunities for immunization.

9. Has the use of the OpenLMIS led to any unexpected effects on vaccine delivery or staff roles? Describe these changes.
   - Streamlined processes and reduced paperwork, allowing staff to focus more on vaccination utilization.
   - Enhanced Accountability Better tracking of stock and usage of the OpenLMIS has increased accountability and transparency.
   - Shift in Roles: Some staff roles have shifted to include more data management and analysis tasks.

D. Learnings and recommendations from OpenLMIS utilization for COVID-19 vaccination

10. What unique lessons were learned (positive and negative) from using OpenLMIS for COVID-19 vaccination, and what recommendations do you have based on these lessons that can be applied to future large-scale campaigns and outbreak response in Nigeria or other countries?
    - **Positive Lessons**:
      - ✔ OpenLMIS can handle large-scale vaccination campaigns, Real-time tracking of vaccine distribution and administration was crucial for timely decision-making and Adaptability of the system to different contexts and needs.
    - **Negative Lessons**:
      - ✔ Challenges in quickly scaling the system to meet the unprecedented demand.
      - ✔ Training Gaps: Initial underestimation of the training required for all users.

- **Recommendations:**
    - ✔ Strengthen the logistics and devices to ensure reliability. Ensuring extensive and ongoing training for all users. Continuously improve the system based on user feedback and emerging needs. Enhance interoperability with other health information systems to provide a holistic view of health data not just for vaccinations

Participant No. 032
Location: Niger
Respondent's Gender: Male

A. **OpenLMIS and its role within the EPI Programme and beyond**

1. What was the rationale for the rollout process of OpenLMIS for COVID-19 vaccination and subsequently Routine Immunization (RI) implementation?

- I said earlier, I have limited knowledge about it, but the openLMIS, comes to serve as a kind of tracking mechanism for vaccine utilization and availability, like a kind of accountability framework in terms of making sure our vaccines are safe at all levels. So I think that is the rationale, so that we can be able to monitor our vaccine across all levels at the LGA/State and also at the national level.

Probe: *So for my clarity, I would want you to make me understand. Was there any difference in how this application is used in RI compared to that of Covid 19? Because I understand that it was rolled out during the Covid-19 implementation*.

- Yeah. It was rolled out during the Covid 19 phase in Niger State here. And basically, it was actually, well utilized and it's actually improved the system in terms of vaccine availability and also monitoring our vaccine utilization at all levels. So the only thing I can say about COVID-19 and also the normal routine immunization when you look at COVID-19 vaccination, it's just one vaccine that we are talking about during the Covid 19. But when we are looking at RI now, we have like almost like nine antigens at the same time that we are tracking at the same time. So even at that time, COVID-19 is a bit simpler because you are only dealing with just a single vaccine. And one component of this OpenLMIS, is that you are going to populate at the LGA level. But for this routine immunization, all aspects of vaccines have to be included/inputted. Then back to the memory. When we started during the Covid 19, there were a lot of knowledge gaps in terms of how the sub-national level that is at the LGA level to populate these things because you know, just at the state level, we just train the LGA CCO once. And in the state, we have been having a kind of shift in the sense that some of these people or these guys that were trained at that level, some have moved to another position at the LGA level, then bringing another person who is not well trained on these devices or these applications on how to populate these.
- So the Covid era is more a kind of easier and less complex than the normal routine

2. Describe the role OpenLMIS currently plays within the EPI program.

- So the openLMIs play a very vital role and as I see it, help in terms of monitoring our vaccines at all levels, especially at our LG stores, in terms of knowing, so that it helps us to avoid overstocking of vaccines in a particular LG or under stockings because it tell us

the level of which a particular LG is within different vaccines, so that if we are now looking at this LGA require restocking, we quickly alert the LGA. Despite in the state, we are not practicing the push method. What we practice in the state currently is pull. That is, the LGA guys come over to the state to pick up the vaccine. So looking at this OpenLMIS, we alert us to inform the LGA. Look, these vaccines are at this level. So you need to forward your request for restocking or re-ordering at the same time. Okay.

Probe: We look at this more, more, more, especially daily, to track the utilization across each LGA. Routine immunization is an activity that is ongoing on a daily basis in most across our LGA. So we don't give the gap to say until the end of the week or till the end of the month doing that. We cannot be able to visualize the utilization and availability of the Vaccine at the LGA.

3. How has OpenLMIS impacted your decision-making and program management?

- Its significance is that when you look at it, like the vaccine supply, we mostly use currently in the state, from the national is projected population, which we mostly believe that it's underestimated. So OpenLMIS guides us to know within this particular period or weekly, how many doses will this particular LGA consume. So not looking at their projected population whatsoever, the OpenLMIS. servers as a guide

Probe - To what extent do you rely on the openLMIS in terms of planning, immunization campaigns, allocating resources, and monitoring program performance? I would want you to be specific about how important is this in the aspect of your planning. How do you rely much on the platform?

- So we rely on the OpenLMIS in planning because the state logistic working group is also saddled with the responsibility of looking at the commodity utilization at the LGA level. And also identify gaps
- We use the OpenLMIS to also plan for our campaigns and other activities in the LGA level or at the state level.

4. To what extent is the OpenLMIS interoperable with other information systems (DHIS2)?

- So, when you look at openLMIS, Both the DHIS and OpenLMIS are all real-time Data Driven documents. So they are all real-time, data-driven applications that help in terms of looking at coverages and other activities that taking place in the various vaccine space. So, but when we look at the OpenLMIS, it guides us in terms of service delivery because we need to know so that we don't kind of send resources because, like, in Niger, we have support from Gavi, that is HSS (health system strengthening) support. So there is a specific fund that is also allocated for each facility to conduct outreaches, fixed sections, and mobile sections. So with these openLMIS, it has helped us to know In a particular LGA. We are planning to conduct this number of outreach sessions. Do we have vaccines available at that LGA, if we don't have this OpenLMIS it very difficult to understand what is available at the LGA level in terms of resource allocation
- From what I've just explained, OpenLMIS validates information about coverage on DHIS2

5. Is the OpenLMIS already being used in other health areas (i.e. for other health commodities)

- From the knowledge I have about the utilization of OpenLMIS Yes, OpenLMIS is being used during Covid19, RI was beyond the initial scope, including the management of

vaccines.

B. Barriers and facilitators to adoption, data quality, and use of OpenLMIS

6. What are the main barriers to the adoption, use, and data quality within OpenLMIS?
   - I believe you might have had this from my other colleague, most factors have been Inadequate or unreliable internet connectivity that has hindered the effective use of OpenLMIS, especially in remote areas.
   - I was also noted that Limited financial support such as data allowance and human resources which we have recommended the possibility have trained assistant officers at the cold-chain facilities has impeded the full implementation OpenLMIS because in the absence of training staff, we lack replacements.

7. What factors have facilitated the successful implementation and use of OpenLMIS?

   - I believe part of the factors that supported and facilitated the successful implementation has to do with the Active involvement and collaboration of stakeholders at all levels to ensure full compliance and address context-specific needs at the state level for encouragement

C. Perceptions on the link between OpenLMIS data use and immunization coverage

8. Have you noted any links between OpenLMIS data use and immunization coverage, completion, and timeliness?
   - Yes, there are notable links between OpenLMIS, the Platform enhances the management of vaccine supply chains by providing real-time data on vaccine stock levels, distribution, and usage. This improved data visibility allows us workers and our senior management to make informed decisions about vaccine utilization across all levels within the state, ensuring that vaccines are available at the right time and facilities. So that immunization can reach the intended community and this has led to higher coverage rates. Additionally, the system helps in monitoring the timely administration of vaccines in a timely manner, ensuring that facilities receive their doses as scheduled, which is crucial for the effectiveness of immunization vaccines. Therefore, the use of OpenLMIS data is instrumental in optimizing vaccine logistics, which directly contributes to better immunization outcomes in the state.

9. Has the use of the OpenLMIS led to any unexpected effects on vaccine delivery or staff roles? Describe these changes.
   - Yes, the use of OpenLMIS has led to several unexpected effects on vaccine delivery, the Platform ability to provide real-time data has improved not only vaccine availability but also streamlined the entire logistics process
   - For example you will find some staff referring to the OpenLMIS as a tool that has made work earlier within the vaccine space and are requesting other programs to emulate that

D. Learnings and recommendations from OpenLMIS utilization for COVID-19 vaccination

10. What unique lessons were learned (positive and negative) from using OpenLMIS for COVID-19 vaccination, and what recommendations do you have based on these lessons that can be applied to future large-scale campaigns and outbreak response in Nigeria or other countries?

- As I had earlier informed from the start, I don't have much knowledge on OpenLMIS so for the challenges. I also need to improve on the utilization of the openLMIS. Looking at the features, how can I navigate through most of it, But for now, sincerely, I can say this is the feature and this is what I can recommend by going forward. I'll take my time and look at it.
- But we all are witness that the use of OpenLMIS's adaptability to the rapidly changing needs during the COVID-19 vaccination campaign demonstrated its scalability for large-scale health programs which I'm highly recommending not just within the vaccine space but across the board, everything is moving digital, the health sector should be included too.

Participant Nos. 033 / 034
Location: Niger
Respondent's Gender: Male /Female

A. The stock management system and flow of data from paper-based to OpenLMIS

1. Describe your role and the stock management system you currently use for vaccine stocks

- Our role as [redacted] varies, she oversees and supervises all the activities that are going on in the immunization and supply chain cycle. I serve as [redacted]
- The OpenLMIS serves as an electronic logistics management information system that helps streamline the management of vaccine stocks by transitioning from paper-based records to the OpenLMIS system contributing to better vaccine availability and administration.

2. Describe the flow of data from health facilities to higher levels of the supply management system.

- The flow of data in a supply management system typically follows these steps to our understanding, this data is often collected through electronic systems the OpenLMIS or through paper-based records which is most common from the frontline due to the lack of availability of monitoring devices to does utilizing the vaccine during campaigns/sensitization but for core distribution at the state level and LGA level were adequate facilities are in place are mandated to effectively use the OpenLMIS to update their records on daily and weekly.
- Reports are shared with higher levels of the supply chain, including national/state supply managers and health ministry officials, which I also believe they also have access to the platform for visibility

B. OpenLMIS user experience, benefits, enablers and barriers

3. What are the main benefits of using OpenLMIS, compared to the paper-based system?

- Since the induction of OpenLMIS, it has been able to Reduce human error associated with manual data entry, Provides up-to-date information on stock levels and consumption rates and we now have a clear view of the supply chain, making it easier to identify and address issues Streamlines data collection and reporting processes, saving time for health workers and Ensures that data is securely stored and easily accessible when needed but OpenLMIS unlike DHIS2 when you can view data from years ago, this platform lacks such features

4. How easy is OpenLMIS to use in your daily work?

- Honestly, for us, OpenLMIS is designed with user-friendly interfaces that simplify data entry and navigation but they can do better by enhancing the dashboard, and just like we had earlier said it has streamlined data reporting processes, saving time and efficient utilization within the vaccine space.

5. Are there any features of OpenLMIS you find particularly helpful or unhelpful?

- The OpenLMIS has made it easy to generate reports for various stakeholders with the state promptly compared to when we were using the paper version and also helps in detailed tracking of inventory movements and usage.
- It will be better if we can get Notifications for low stock levels or upcoming expirations via SMS or email to serve as the remainder, and Occasional technical issues or downtime has hindered usability which could be attributed to network service at times.

6. What factors have enabled you to effectively use OpenLMIS in your work?
   - Well, we usually hold technical working group meetings, where we discuss and support each other with a bit of refresher training sessions for all users, this fosters Encouragement and support even though we have our challenges that have not been addressed
   - Mandatory compliance from all levels for the utilization of the OpenLMIS

7. What challenges have you experienced with using OpenLMIS?

- Most of the reports coming to us are related to technical issues which are downtime and Problems with internet connectivity
- Inadequate hardware because some of the report devices for accessing the OpenLMIS are damaged and have been replaced
- The usual encouragement for data allowance has been suspended, staff has to devices other means to get data and sometimes use their personal resources to do the job
- Trainings. Insufficient training for all users which I recommend action should be taken because we have new staff onboard due to transfer or promotion

C. Recommendations and improvement

8. What improvements would you suggest to make OpenLMIS more effective for future mass vaccination campaigns as well as routine immunization?
   - Enhanced User Training Regular and updated training programs for all users.
   - Offline Capabilities: our understanding is that most information systems can work office so OpenLMIS should have offline functionalities to mitigate issues with internet connectivity.

- User Feedback Mechanisms: Implementing ways to gather and act on user feedback to continuously improve the system.
- Mobile Accessibility: Enhancing mobile app features to make the system more accessible in remote areas.
- Scalability: Ensuring the system can handle increased data load during mass vaccination campaigns.
- Real-Time Analytics: Incorporating advanced analytics to provide real-time insights and forecasts for top management because most officers are training to carry out simple analysis.

Participant No. 035
Location: Niger
Respondent's Gender: Male

A. The stock management system and flow of data from paper-based to OpenLMIS

1. Describe your role and the stock management system you currently use for vaccine stocks

- Thank you very much for the opportunity given to me. You know, the vaccine, I think, if you understand, it's using the OpenLMIS and paperwork data, So, we are using both at the LGA, because the health facility are collected vaccine from the cold store. We have the ledger books for storing while at the state also. Then at the end of the month, we now use the e-copy in documenting all the vaccine. It's the CCO that is handling the OpenLMIS data platform.

2. Describe the flow of data from health facilities to higher levels of the supply management system.
- There is no issue with the data flow from the health facility or this from LG or to the state. everything, though, is not 100% but the flow of data, think I can say, is okay.
- The data flow is from the health facility to the LG or the LGA to the state, to the national

B. OpenLMIS user experience, benefits, enablers and barriers

3. What are the main benefits of using OpenLMIS, compared to the paper-based system?

- The paper, the paperwork and OpenLMIS, is just to avoid data missing it. you understand paper can be missing but the OpenLMIS is there in the system. And it's easier in terms of computing. Once you compute or input numbers like that, it will now calculate itself.

4. How easy is OpenLMIS to use in your daily work?

- I think is not difficult, according to the CCO that they use it and is fast in reporting. Once you have your data, once you import in at least and send it, will not be difficult in sending it to the appropriate channel, and you have so it makes work easier.
-

5. Are there any features of OpenLMIS you find particularly helpful or unhelpful?

   - I think a lot, because we even sat down after they came back from the training. I sat down with the CCO. I asked him, how is it? Is there any area that you have assigned? He said, No.

6. What factors have enabled you to effectively use OpenLMIS in your work?

7. What challenges have you experienced with using OpenLMIS?

I can only say, number one, the computer is using is having challenges with that. In terms of reporting, though they gave them that tablet and even that one, it got broken. They even asked them to return to the national, to the state, so that they will replace them with new once because the OpenLMIS. Even that one too, you don't have it now, his system too, you don't have it. Then support, also data support. I don't think the data support is always.

C. Recommendations and improvement

8. What improvements would you suggest to make OpenLMIS more effective for future mass vaccination campaigns as well as routine immunization?

Number one, they should train CCO. They should add number of people like the LIOs for the next training of the OpenLMIS so that everyone will be in picture of all what is going on. Number two, we still have issue with the gadgets. Also, they should provide support data steepens.

Participant No. 036
Location: Niger
Respondent's Gender: Male

A. OpenLMIS and its role within the EPI Programme and beyond

1. What was the rationale for the rollout process of OpenLMIS for COVID-19 vaccination and subsequently Routine Immunization (RI) implementation?

   - *OpenLMIS as a software, was a child of necessity within the Nigerian immunization system, because at a point we were using an LMIS system before, which was online, and it provided visibility, of vaccine stocks across several storage levels. However, there was a time of transition where we had to move back to Excel based documents, which had some limitations. So, the nation, as a whole thought to bring something that is more user friendly, that will, restore that visibility. So openLMIS is here to provide us with visibility of the vaccine situations in every storage location, show evidence of transaction, the movement of vaccines from the National all the way to the facilities, the accountability, the reporting aspect of it.*

2. Describe the role OpenLMIS currently plays within the EPI program.

   - [redacted] *we're working hand in hand with my* [redacted] *on vaccine management. Definitely, whatever we are doing, we do it collectively to see that our vaccines are intact is in our culture, and first of all, to look at the balance of vaccine on weekly basis, the*

one we receive and the ones that went out, so that at every end of the week, my [redacted] *will enter it in OpenLMIS and send it to state on weekly basis, and the delivery that is receiving from state. When she come back, she used to do it again, both in OpenLMIS and hard copy that we are keeping seeing that what is going out and what we are receiving.*

Probe: So, I think what I wanted to say is that do you now as the [redacted] and your team use the OpenLMIS to make your decision, and how do you do that?

- *Yeah, definitely. The decision I'm making, the OpenLMIS help me compare what we have used check if there is any discrepancies between those who are simply receiving, and ask why do you have these discrepancies? We proffer solution.*

3. How has OpenLMIS impacted your decision-making and program management?

- *It is very fast in sending a report. Formally you must travel to the state capital Minna but now within your office you will just send your report, and you can see from the system when you are stock out and can make decision*

4. To what extent is the OpenLMIS interoperable with other information systems (DHIS2)?

- *The only thing I know is that this will be integrated and when it happens it will make sense*

5. Is the OpenLMIS already being used in other health areas (i.e. for other health commodities)
   - *The OpenLMIS can handle the vaccines, both routine and supplementary, including the covid 19 vaccines. It has an aspect for the cold chain equipment, where stores can enter their cold chain inventories and it also has a component for other commodities, such as consumables, nutrition products. those are also a component within the OpenLMIS*

B. Barriers and facilitators to adoption, data quality, and use of OpenLMIS

6. What are the main barriers to the adoption, use, and data quality within OpenLMIS?

- *You know whatever you are doing electrically, you have it immediately, and you send it to server, this hard copy, takes a month or some even before sending it to server, even because it undergoes different you process send it to the state, from state to national. But in OpenLMIS is immediately, the OpenLMIS help us to send directly to server. so you know everything now is going immediately, when you send your report, it will just go up to server to know what we are having here a fridge.*

Probe: So, how does open LMIs improve your work as a person presently?

- [redacted] *the only thing we are having now is that it was CCO that they train on the OpenLMIS, LIO have not been trained. But when she come back, she*

explained it to me, and honestly, it really assisting us here, in terms of keeping in the data of the vaccines.

Probe: So how easy is OpenLMIS is used in your daily work daily work routine?

- *Yes, but only thing is that expenses on data that it used to cost me a lot of data (internet). And whatever you're doing today, if you are not financially buoyant is not going to be easy for you to carry out the assignments, but on daily basis, at times, CCO will use 2000-naira internet within a week it will be finished. So, it's not easy.*

7. What factors have facilitated the successful implementation and use of OpenLMIS?
   - *It is easy at work with, so it saves times than writing hardcopy. But the only challenge is this network issue, at times she will send thinking that the message has gone, and at the end it she will discover that it did not go, and at time it will go multiple time.*

Probe: We have individual factors, we have organization, environmental factors, and then we have technological factors. Do you have any factor affecting the use of OpenLMIS.

- *I'm just appealing to the organization that you come from, you know, it's only CCO that was train on this OpenLMIS, like we as [redacted], we supposed to be train also, so that we can work as a team, if there is any error we can be able to correct one another. We find it very difficult at times, because whenever, you attended training, and you know what is going on, and you know the fundamental things here, it will be hard for you to forgotten something, but somebody go to training and come back and tell you how. Okay, my suggestion is that we're hoping the organization can be able to call LIOs also, so that they should train us.*

C. Perceptions on the link between OpenLMIS data use and immunization coverage

8. Have you noted any links between OpenLMIS data use and immunization coverage, completion, and timeliness?
   - *I think there's a direct link there. I keep mentioning the visibility now because prior to the implementation of the OpenLMIS, that visibility wasn't there. So, if there's a stock out, in state A before that is being flagged up at the higher level where decision can be taken to replenish that state with the required antigens, it takes a bit of time.*

9. Has the use of the OpenLMIS led to any unexpected effects on vaccine delivery or staff roles? Describe these changes.
   - *Nothing to be honest, because the utilization aspect of it, whether request is formally made through the platform or manually. The system has that flexibility*

D. Learnings and recommendations from OpenLMIS utilization for COVID-19 vaccination

10. What unique lessons were learned (positive and negative) from using OpenLMIS for COVID-19 vaccination, and what recommendations do you have based on these lessons that can be applied to future large-scale campaigns and outbreak response in Nigeria or other countries?

- *First thing is providing more training and increase number of participants to included LIOs.*
- *Second, finance because issue of buying these bundles of data for this network issues It's not easy at times, using personal salary and buying this data bundles, at times, they use to send money to the us but is not enough and the money don’t come always. So, we used to face a problem financially, honestly speaking, if the organization can assist in providing all those things for us, money for data should not be a problem and network issue.*

Participant No. 037
Location: Niger

Respondent’s Gender: Male

A. The stock management system and flow of data from paper-based to OpenLMIS

1. Describe your role and the stock management system you currently use for vaccine stocks

- [redacted] My major role is to collect vaccines from the state, bring them to the LGA and take it into my ledger and stored the vaccine within close to two degrees centigrade in my Iceland fridge. So, from Monday to Friday, every facility within the LGA comes to collect vaccine based on target required for vaccination. I distribute these vaccines to them daily to these facilities.
-

2. Describe the flow of data from health facilities to higher levels of the supply management system.

- The basic mode of operation is, we place order to the State Cold Store based on requests made from the Ward health facilities, and when supply is made, we distribute to the various facilities. We afterwards, collect reports from the various health facilities and document it in the ledger and also enter such data with our stock balance in the LMIS. This is usually done every Thursday of the week after conducting Physical Counting.

B. OpenLMIS user experience, benefits, enablers and barriers

3. What are the main benefits of using OpenLMIS, compared to the paper-based system?

- Actually, I can say the best practice of using this open LMIS is with the help of this open LMIS we can be able to gather our report and send to the States and it is also available for everyone to monitor the movement of vaccines. It has also reduced the burden of supervisors from the National office coming all the way to supervise and verify our activities every time. With the management system, they are informed about current

stock level of vaccines. The system has however, minimized challenges encountered in transmitting data in comparison to the actual record in our ledger.

-

4. How easy is OpenLMIS to use in your daily work?

- Even though I have not been trained on the use of the platform, with my experience with other platforms, I would say it is easy to use.
-

5. Are there any features of OpenLMIS you find particularly helpful or unhelpful?

- Actually, there is very essential improvement in using the Open LMIS, because it provides quick access to anyone willing to find out the stock balance of the facility without having to wait for the routine reporting period or traveling all the way here before getting such information. By accessing the Open LMIS, such information is readily available.
-

6. What factors have enabled you to effectively use OpenLMIS in your work?
    - I wouldn't say I am much familiar with the platform as I have not received any training on it, like I said, my familiarity with other platforms made it easy to work with it. The Cold Chain Officer is primarily the person using the platform, but due to our collaboration, I get to understand the various operations of the system.
    - I think the most important thing, you must be sure that you have data on your phone, then you can now be able to click it to start whatever you want to do. Provided there is data and good network.

7. What challenges have you experienced with using OpenLMIS?

I think the major challenge has to do with the availability of data and network. Most at times, the data may be available, but the network will be poor to access the platform. For instance, we conduct our physical counting and send also send report of our stock level. When it happens the network is poor, this will be delayed. This is a usual challenge.

C. Recommendations and improvement

8. What improvements would you suggest to make OpenLMIS more effective for future mass vaccination campaigns as well as routine immunization?
   As I have stated earlier, I was not familiar with the platform from the beginning when it was introduced and was not trained on how to use it. From the way I see it, it is a good tool to use in monitoring the use of vaccines and I think it is perfect as it is.
    - I think, based on what I see him doing, I don't see the need for any modification to the system because it has covered all the necessary task that has to do with the movement of vaccines and reporting.

Participant No. 038

Location: Niger
Respondent's Gender: Male

A. **The stock management system and flow of data from paper-based to OpenLMIS**

1. Describe your role and the stock management system you currently use for vaccine stocks

- [redacted], I received vaccines and push to health facility and on a weekly bases I ger report back from the health facility for my records and send to the state. Normally we use the WhatsApp platform by Send weekly report to state CCO but with the introduction of the OpenLMIS, it has made the work easier for us because it is now simpler than before.
-

2. Describe the flow of data from health facilities to higher levels of the supply management system.

The flow of data start from the grassroots facility. The facility at the grassroot sent us the data using hardcopy or Sending through The WhatsApp Platform and we use this information to update the OpenLMIS.

B. **OpenLMIS user experience, benefits, enablers and barriers**

3. What are the main benefits of using OpenLMIS, compared to the paper-based system?

- Yes, the OpenLMIS has been instrumental to my work. Because before where using paper for your record keeping and this has some resulted to loss of data from health facilities, But using OpenLMIS has make it more easier. Despite we were having problem at the initial stage with has been resolved. So, it's very important to me it's easier with OpenLMIS. You know what is going on, you know your balance, and you know, your Physical balance. So OpenLMIS is very important to me.
-

4. How easy is OpenLMIS to use in your daily work?

- Yes. As I said it has been easy for me. Because with the introduction of using electronic means of sending information through the OpenLMIS and devises giving to us. Sending information from health facilities to LGAs has been a good initiative, with just a click now I can understand what is happen in the LGAs immunization space because with OpenLMIS, I can see what balance are and status of all vaccine as against the paper based version.
-

5. Are there any features of OpenLMIS you find particularly helpful or unhelpful?

- Yes, as I said earlier, the OpenLMIS simplifies the work for us here and shows you, your data. So it's very important but currently my device is faulty due to my involvement in an

accident, I can only use some else phone to update my data on weekly bases but I've reported the issue and I was told to wait.

-

6. What factors have enabled you to effectively use OpenLMIS in your work?
   - Yes, the state has mandatory made the use of OpenLMIS across board to ensure vaccination are utilized effectively and at the onset we were provided with data.

7. What challenges have you experienced with using OpenLMIS?

The only challenge I have with it is data. Data should be provided for us as they were used to doing.

C. Recommendations and improvement

8. What improvements would you suggest to make OpenLMIS more effective for future mass vaccination campaigns as well as routine immunization?

I think the Open LMIS is working well and is serving me. I can't think of anything that should be added to it.

Participant No. 039
Location: Niger
Respondent's Gender: Female

A. The stock management system and flow of data from paper-based to OpenLMIS

1. Describe your role and the stock management system you currently use for vaccine stocks

   - *I am the [redacted] and we are using all the solar refrigerator to store all our vaccines accordingly because we have the vaccine that is Live Attenuated Vaccine and the attenuated vaccine those powders ones so like OPV, BCG, and yellow fever, we store them in down baskets. While IPV, rota and other ones who stood in them at centre of the basket, then the top of the basket to put like painter, PCV and TD on top. We also have thermometer inside that those fridges we're monitoring the temperature morning and evening. In the morning, eight o'clock, temperature, then four o'clock close temperature.*

2. Describe the flow of data from health facilities to higher levels of the supply management system.

   - *We used to supply RI to health facilities, which is 2 part which are Lapai which covers 5 wards and the Anza part with 5 wards. Meanwhile in Lapai Health facility running RI is almost 20 and in the Anza part we have 25 health facility in the Anza Glow part there is cold store who is supply SDD Sula, also, DC the blue 200 as the 2000 SDD, so I normally supply them with vaccine monthly when I go to Minna collected my own vaccine from the state, then will supply them from here also. I used to collect the reports at the end of the month, how the vaccine is being supplied to the VM, one vaccine*

utilization, then all those things that we're collecting, we always archived according to the months, like this month that we're in now, if the RI report is ready, and everything is ready, I'll tie them up and write the date of that month's on top and archive. Is also the same here. In OpenLMIS we do collect the stock balance I used to have in my fridge, I'll go to my fridge and count my physical vaccine that I have at hand. that physical account is the one that I used to enter the OpenLMIS is both my dry stock and the vaccine. So, I'm carrying out every Thursday.

***Probe**:* have you ever experienced or had an experience where what you report on your opponent is different from the actual stock at hand? after doing your physical counting?

- *We do have that problem, even this morning, because last week, I assigned my assistants to count for me and I enter it because I was Minna then I told him to count and sent to me the way he counts main A was not complete like one I Count yesterday, there's additional nine vial. So, this morning, as I counted that mine, show up and tie it into my OpenLMIS you will find out that my OpenLMIS now have accreditation instead of transfer out i credits the remaining nine*

Probe*:* So, was this a major problem? How did you tend to correct that problem?

- *No, it's not a major problem. No, we are used to it, if you have any additional vaccine that is left out. You add it up that is an adjustment to give us actual that we have. Because what do you have your in OpenLMIS will be the same as what in your ledger and it will be the same as what you have in your fridge. So, maintain that level*

B. OpenLMIS user experience, benefits, enablers and barriers

3. What are the main benefits of using OpenLMIS, compared to the paper-based system?

- *it has many benefits since this OpenLMIS is open although since they thaught us how to use this OpenLMIS I don't have another problem because I enjoy the OpenLMIS than the paper one that we're doing before and sending ODK because in that one that we're doing, sometimes as we correct, this one even that one you can correct it but this one is more perfect to me than once and my challenge is network problems sometimes. Because sometimes you are looking for network there you will enter some, it will not even go you have to enter multiple times and sometimes I will have to leave here to outside here to access good network. And our problem again we need assistance internet issues.*

4. How easy is OpenLMIS to use in your daily work?

- *All the vaccine supply are done from the states, instead of us to enter it, they will enter it. If you just go to your OpenLMIS you just go through it for validation. Meanwhile, previously, we were the ones receiving and after receiving at the end of the day, you will now do physical account. Unlike now that we do only physical accounts to validate. So, it is very easy now.*

5. Are there any features of OpenLMIS you find particularly helpful or unhelpful?

- *For me I like all the features of the OpenLMIS and I am comfortable with it there is no one that I have issues with because the OpenLMIS made work easy and fast. But like I say the area of stock management is one part that I enjoy more.*

6. What factors have enabled you to effectively use OpenLMIS in your work?
- *For me is technology in this case internet is technology. Because even when we are given a lecture, we use technology so technology for me is the major factor.*

Probe: Can you describe the training and support you have received to enable the implementation of OpenLMIS

- *The first training we receive was in the States around 2022 about 2 years now. First one training was by one Dr. Owen train us with our state coordinator, and all the state team using OpenLMIS*

7. What challenges have you experienced with using OpenLMIS?

- *Apart from the once I mentioned earlier. That of my tablet that got broken. Because I was living here on Thursday evening to my house, and I took bike and I had accident both my phone was broken with even my laptop because I fell inside pits (gutter) as we got that accident, the phone beyond repair so I had to get a new phone again I quickly inform the state what happened to me and my phone. But along the way I took it to fix screen. They told me that the screen is not yet in the market. I have to bring it out. So, what I did, I try using my phone and it worked. And I can effectively use my phone.*

C. Recommendations and improvement

8. What improvements would you suggest to make OpenLMIS more effective for future mass vaccination campaigns as well as routine immunization?
- *For me if we can be using it offline so we can use it even where there is no network connection for effectiveness and then if we reach the point of sending each time, we get to a network connection area we will just send it. Because without data you cannot open it and is really frustrating at times*

Probe: So, since you started using the OpenLMIS has there been any room for improvement.

- *Yes, very well even. Formally we started with health facility doing counting that does not always tally but letter they thought us physical count and it help a lot. So physical count gives us opportunity to count everything.*

Participant No. 040
Location: Niger

Respondent's Gender: Male

A. **The stock management system and flow of data from paper-based to OpenLMIS**

1. Describe the flow of data from health facilities to higher levels of the supply management system.

- Actually, the data received from these facilities are brought in on monthly basis to the LGA. The data are received and compiled according to Wards and according to health facility, and send a soft copy to the State. We keep the hardcopy with us here.
-

B. **OpenLMIS user experience, benefits, enablers and barriers**

2. What are the main benefits of using OpenLMIS, compared to the paper-based system?

- Actually, I can say the best practice of using this open LMIS is with the help of this open LMIS we can be able to gather our report and send to the States and it is also available for everyone to monitor the movement of vaccines. It has also reduced the burden of supervisors from the National office coming all the way to supervise and verify our activities every time. With the management system, they are informed about current stock level of vaccines. The system has however, minimized challenges encountered in transmitting data in comparison to the actual record in our ledger.
-

3. How easy is OpenLMIS to use in your daily work?

- Actually, it is easy. It simplifies the entire process of reporting our weekly activity. As reporting is done weekly, we manually document all daily processes and then have the records inputted into the Open LMIS. The reporting is done every Wednesday of the week. Before this is done, we would have collected all data from the ward facilities. It also gives us track of all that has been distributed and what is left in stock including the life span of the vaccines.
- Actually, before now, it turned out to be difficult to use due to its complexity especially in terms of. But along the way, I now see it to be more compatible to my work in terms of reporting. Today, I enjoy using it.
-

4. Are there any features of OpenLMIS you find particularly helpful or unhelpful?

- The particular function I find very helpful about the platform has to do with identifying a physical inventory by inputting its specific code that will take you directly to it instead or searching through a numerous list of other tasks.
-

6. What factors have enabled you to effectively use OpenLMIS in your work?

- If you can provide us with power bank because of power failure times because there is a high level of risk involved if we are to take the device to road side charging vendors to have the device charged. These risks could be theft or accidental damage of the device by the vendors.
-

7. What challenges have you experienced with using OpenLMIS?

As I said earlier, internet data is needed. Initially, we were provided with data subscriptions directly to our designated lines. This stopped after about two months. Also, the need to provide us with power bank to power the device when power supply is not available.

C. Recommendations and improvement

8. What improvements would you suggest to make OpenLMIS more effective for future mass vaccination campaigns as well as routine immunization?
   - Yes. My first suggestion is, like I said earlier, the organization should try and be providing us with data. The second issue provision of Power bank to support us where there is power failure and the inclusion of Wards within the LGA where these facilities are located to improve the credibility of documentation in the system.
   -

Participant No. 041 / 042
Location: Niger

Respondent's Gender: Male / Male

A. The stock management system and flow of data from paper-based to OpenLMIS

1. Describe your role and the stock management system you currently use for vaccine stocks
   - *At the [redacted] with the coming of this software, it has been helpful to us, because the time that we start using the software before we were using VAN visibility analytic. So, the differences between the van and OpenLMIS is that you see, for the van, they impute the minimum and maximum of each local government, but in the open LMIs, the issue of minimum and maximum is not in there. So, at times, under the population, they use in building the open LMIs, they use that of HMIS as population, which NHIS population is a bit is lower than the real target population. So, I handle all cold chain activities here at the zonal store.*

2. Describe the flow of data from health facilities to higher levels of the supply management system.
   - *Well, is simply from the health facilities to the LGA Cold chain, then to the state from the state to the Zonal level in ideal case but sometimes the state relates with the national*

B. OpenLMIS user experience, benefits, enablers and barriers

3. What are the main benefits of using OpenLMIS, compared to the paper-based system?

- *Is stress free and very helpful, because now, if you go to my office, any transaction that took place the Moment, I don't have to suffer myself writing. I'll just after I issue out, I have a printer out for documentation.*

4. How easy is OpenLMIS to use in your daily work?
    - *Very easy in fact it makes the work very easy and is given us the require expected result*

5. Are there any features of OpenLMIS you find particularly helpful or unhelpful?

- *If there's a way to make it easier and short so that you go for any program that you need. Also, before you get hired to reduce the procedural you take the program and be ready and under him there should be a computer or laptops that we have with us dedicated for device for the OpenLMIS work and not for personal use.*

Probe; So, you are equally recommended that a device should be dedicated for this specific, what device would you recommend laptop?
    - *A Higher version laptop will be nice.*

6. What factors have enabled you to effectively use OpenLMIS in your work?
- OpenLMIS is accessible and easy to navigate
- It doesn’t need too much data bandwidth
- And the fact that it makes the work very easy.

7. What challenges have you experienced with using OpenLMIS?
    - *The major challenge for us here is not data support most of the time we run this with our personal funds, and we are getting same report from our down liner*

C. Recommendations and improvement

8. What improvements would you suggest making OpenLMIS more effective for future mass vaccination campaigns as well as routine immunization?

- *If there's a way to make it easier and short so that you go for any program that you need. Also, before you get hired to reduce the procedural you take the program and be ready and under him there should be a computer or laptops that we have with us dedicated for device for the OpenLMIS work and not for personal use.*
