# Supplementary material for: Enablers and barriers to implementing a digital logistics system: A qualitative evaluation of OpenLMIS for Nigeria’s vaccine supply chain
Source: PLOS Glob Public Health. 2026 Sep 15;6(9):e0005581. doi: 10.1371/journal.pgph.0005581 (PMC13577329; doi:10.1371/journal.pgph.0005581)
Supplement: Supplementary file 2 [file pgph.0005581.s008.pdf]

S/No.	Create Physical Inventory	Make Issue - Issue an Order	Make Receive - Confirm Shipment	Make Adjustment - Rectify stock balance where there is an over-issue of a commodity	Transfer stock items from one program to another (Non-Polio SIA to RI)	Stock on Hand / Stock Status Report - Generate stock balance report	Generate report showing Stock expiry status of products	Generate stock distribution report	Manage Cold Chain Equipment
001	Yes	No	No	Yes	N/A	N/A	N/A	N/A	No
002	Yes	Yes	No	Yes	N/A	N/A	N/A	No	No
003	Yes	Yes	No	Yes	N/A	Yes	N/A	N/A	No
004	Yes	Yes	Yes	Yes	Yes	Yes	Yes	Yes	Yes
005	Yes	Yes	Yes	Yes	Yes	No	No	No	No
006	Yes	Yes	Yes	Yes	Yes	Yes	Yes	Yes	Yes
007	Yes	Yes	Yes	Yes	Yes	Yes	Yes	Yes	Yes
008	Yes	Yes	Yes	Yes	N/A	Yes	N/A	N/A	Yes
009	Yes	Yes	Yes	Yes	N/A	Yes	N/A	N/A	Yes
010	Yes	Yes	Yes	Yes	Yes	Yes	Yes	Yes	Yes
011	Yes	Yes	Yes	Yes	N/A	Yes	N/A	N/A	N/A
012	No	Yes	Yes	Yes	Yes	Yes	No	Yes	Yes
013	Yes	Yes	Yes	Yes	Yes	Yes	Yes	Yes	Yes
014	Yes	Yes	Yes	Yes	N/A	N/A	N/A	N/A	N/A
015	Yes	Yes	Yes	Yes	N/A	N/A	N/A	N/A	Yes
016	Yes	Yes	Yes	Yes	N/A	N/A	N/A	N/A	Yes
