# Supplementary material for: Enablers and barriers to implementing a digital logistics system: A qualitative evaluation of OpenLMIS for Nigeria’s vaccine supply chain
Source: PLOS Glob Public Health. 2026 Sep 15;6(9):e0005581. doi: 10.1371/journal.pgph.0005581 (PMC13577329; doi:10.1371/journal.pgph.0005581)
Supplement: Supplementary file 3 [file pgph.0005581.s001.docx]

**S1 Annexe: List of Documents**

1. electronic Logistics Management Information System - a Literature Review (referencing 46 publications)
2. Nigeria OpenLMIS Registered users
3. Nigeria OpenLMIS user guide
4. Nigeria OpenLMIS Utilization rates
   a. 2023 Utilization Reporting Rates - Lagos, FCT, Nasarawa, Niger
5. Nigeria OpenLMIS Covid 19 Grant Lessons Learnt
6. Nigeria OpenLMIS RI_SIA_COVID Grant Lessons Learnt
7. Nigeria OpenLMIS Theory of Change

Nigeria OpenLMIS Deployment Overview
