# Supplementary material for: Enablers and barriers to implementing a digital logistics system: A qualitative evaluation of OpenLMIS for Nigeria’s vaccine supply chain
Source: PLOS Glob Public Health. 2026 Sep 15;6(9):e0005581. doi: 10.1371/journal.pgph.0005581 (PMC13577329; doi:10.1371/journal.pgph.0005581)
Supplement: Supplementary file 5 [file pgph.0005581.s003.docx]

## S3 Annexe: Key Informant Interview Guide: Implementers and Program Managers

*CHAI, NPHCDA, Director Logistics and Health Commodities, Members of the National Logistics Working Group. National Strategic Store - Logistics Officers, State Director Immunisation/ Disease Control, and Vaccine Security and Logistics Officers - UNICEF*

**Respondent’s Gender**: Male Female

1. **OpenLMIS and its role within the EPI Programme and beyond**
   a. What was the rationale for the rollout process of OpenLMIS for COVID-19 vaccination and subsequently Routine Immunisation (RI) implementation?

*Are there any differences in how the application is used in RI vs. the COVID-19 vaccination*?

b. Describe the role OpenLMIS currently plays within the EPI program.

*How do you use the OpenLMIS on a daily, weekly, or monthly basis?*

*To what extent do you rely on OpenLMIS data for planning immunisation campaigns, allocating resources, and monitoring program performance? Please give examples of how you used OpenLMIS for any of these functions.*

c. How has OpenLMIS impacted your decision-making and program management?
d. To what extent is the OpenLMIS interoperable with other information systems (e.g.DHIS2)?
e. Is the OpenLMIS already being used in other health areas (i.e. for other health commodities)

2. **Barriers and facilitators to adoption, data quality, and use of OpenLMIS**
   a. What are the main barriers to the adoption, use, and data quality within OpenLMIS?

*Probe for any specific user groups (Cold Chain officer/Program manager, male or female) within the program/supply chain who have difficulty adopting or using OpenLMIS effectively, and why*

*Probe further for specific factors (individual, organisational, and/or technological)*

*Probe for variations in OpenLMIS usage in different States/LGAs and reasons for this.*

*Probe for barriers hindering gender equity in access to and utilisation of OpenLMIS, and what strategies can be employed to address these barriers effectively?*

*Probe for any traditional gender roles and stereotypes within organisational facilities, and how these impacts can be mitigated*

b. What factors have facilitated the successful implementation and use of OpenLMIS?

*What strategies have been successful in overcoming barriers to OpenLMIS adoption & improving data quality?*

*Which organisations are providing funding for system implementation and maintenance?*

*How often are training activities around LMIS conducted (who implements training, what is the frequency?)*

*Do you think there are enough resources directed towards openLMIS?*

3. **Perceptions on the link between OpenLMIS data use and immunisation coverage**
   a. Have you noted any links between OpenLMIS data use and immunisation coverage, completion, and timeliness?

*Describe specific correlations between data use and improved program outcomes (e.g., higher coverage rates in higher LMIS usage locations). Is there any evidence to support these findings?*

b. Has the use of the OpenLMIS led to any unexpected effects on vaccine delivery or staff roles? Describe these changes.

4. **Learnings and recommendations from OpenLMIS utilisation for COVID-19 vaccination**
   a. What unique lessons were learned (positive and negative) from using OpenLMIS for COVID-19 vaccination, and What recommendations do you have based on these lessons that can be applied to future large-scale campaigns and outbreak response in Nigeria or other countries?

*What factors facilitated such rapid national scaling of the OpenLMIS during COVID ((infrastructure to support OpenLMIS, policy changes, political buy-in)?*

*Are there any specific features or functionalities you would recommend adding to OpenLMIS to prepare for future outbreaks or mass vaccination needs?*
