# Supplementary material for: Enablers and barriers to implementing a digital logistics system: A qualitative evaluation of OpenLMIS for Nigeria’s vaccine supply chain
Source: PLOS Glob Public Health. 2026 Sep 15;6(9):e0005581. doi: 10.1371/journal.pgph.0005581 (PMC13577329; doi:10.1371/journal.pgph.0005581)
Supplement: Supplementary file 6 [file pgph.0005581.s004.docx]

## S4 Annexe: Key Informant Interview Guide: Cold Chain Officers

*Zonal Cold Chain Officers, State Cold Chain Officers, Vaccine Security and Logistics Officers - UNICEF, Satellite Cold Chain Officers, LGA Cold Chain Officers.*

**Respondent’s Gender**: Male Female

1. **The stock management system and flow of data from paper-based to OpenLMIS**
   a. Describe your role and the stock management system you currently use for vaccine stocks
   b. Describe the flow of data from health facilities to higher levels of the supply management system.

*How often were there any discrepancies between recorded and actual stock levels? Can you share any specific examples? Is this a major problem?*

2. **OpenLMIS user experience, benefits, enablers, and barriers**
   a. What are the main benefits of using OpenLMIS, compared to the paper-based system?

*Probe for specific examples of how OpenLMIS has improved their work.*

b. How easy is OpenLMIS to use in your daily work?

*Probe for specific tasks in their daily work that OpenLMIS makes easier or harder.*

*Probe for how well OpenLMIS integrates with their existing workflow.*

*If it integrates well, probe for an example of how OpenLMIS integrates well with their workflow. If not, probe for where it creates friction.*

c. Are there any features of OpenLMIS you find particularly helpful or unhelpful?

*Probe if there are any features or functionalities they wish OpenLMIS had that they need to perform their job effectively, or any existing features they rarely use, and why.*

*Probe the value of the decision-support algorithms, alerts, and reminders, and if this actually helped them prevent stockouts or make better decisions. Ask for an example.*

d. What factors have enabled you to use OpenLMIS in your work effectively?

*Probe further for specific factors (individual, organisational/environmental, and/or technological).*

*Ask them to describe the training and support they have received to enable the implementation of OpenLMIS. How often are training activities around LMIS conducted (who implements training, what is the frequency?)*

*Are there enough resources directed towards openLMIS? Do you think it’s valued by those in charge?*

*Which organisations are providing funding for system implementation and maintenance?*

e. What challenges have you experienced with using OpenLMIS?

*Probe further for specific factors (individual, organisational/environmental, and/or technological)*

*Ask if they feel they have the necessary skills and training to use OpenLMIS effectively. If not, ask what additional support they need.*

3. **Recommendations for improvement**
   a. What improvements would you suggest to make OpenLMIS more effective for future mass vaccination campaigns as well as routine immunisation?

*Have there been any improvements/investments into openLMIS since it was launched*
