# Supplementary material for: Enablers and barriers to implementing a digital logistics system: A qualitative evaluation of OpenLMIS for Nigeria’s vaccine supply chain
Source: PLOS Glob Public Health. 2026 Sep 15;6(9):e0005581. doi: 10.1371/journal.pgph.0005581 (PMC13577329; doi:10.1371/journal.pgph.0005581)
Supplement: Supplementary file 7 [file pgph.0005581.s005.docx]

## S5 Annexe: OpenLMIS Observation Checklist

**Date:** __________	**Location:** __________	**User Role:** ____________

General Instructions:

●	Observe users interacting with the OpenLMIS platform. If the user is not implementing specific tasks to be observed, ask the user to demonstrate at least 3 relevant tasks to their role.

●	Document any challenges, or suggestions expressed by the user regarding specific functionalities.

| **Function to Observe** | **Was the user able to complete the process?** | **Did they encounter any challenges?** | **Describe the challenges** | **Ask the user for Improvement suggestions** |
| --- | --- | --- | --- | --- |
| Create Inventory | Yes 🗸 No 🗸 N/A 🗸 | Yes 🗸 No 🗸 |  |  |
| Request Stock | Yes 🗸 No 🗸 N/A 🗸 | Yes 🗸 No 🗸 |  |  |
| Approve Requisition | Yes 🗸 No 🗸 N/A 🗸 | Yes 🗸 No 🗸 |  |  |
| Fulfil an Order | Yes 🗸 No 🗸 N/A 🗸 | Yes 🗸 No 🗸 |  |  |
| Confirm Shipment | Yes 🗸 No 🗸 N/A 🗸 | Yes 🗸 No 🗸 |  |  |
| Request emergency stock | Yes 🗸 No 🗸 N/A 🗸 | Yes 🗸 No 🗸 |  |  |
| Approve Emergency stock requisition | Yes 🗸 No 🗸 N/A 🗸 | Yes 🗸 No 🗸 |  |  |
| Convert Requisitions to Actual Physical Order | Yes 🗸 No 🗸 N/A 🗸 | Yes 🗸 No 🗸 |  |  |
| Manage Cold Chain Equipment with RTM | Yes 🗸 No 🗸 N/A 🗸 | Yes 🗸 No 🗸 |  |  |
| Analyse vaccine stock management and distribution | Yes 🗸 No 🗸 N/A 🗸 | Yes 🗸 No 🗸 |  |  |
