# Supplementary material for: Enablers and barriers to implementing a digital logistics system: A qualitative evaluation of OpenLMIS for Nigeria’s vaccine supply chain
Source: PLOS Glob Public Health. 2026 Sep 15;6(9):e0005581. doi: 10.1371/journal.pgph.0005581 (PMC13577329; doi:10.1371/journal.pgph.0005581)
Supplement: Supplementary file 9 [file pgph.0005581.s002.docx]

## S2 Annexe: Theory of Change: OpenLMIS implementation and use in Nigeria
